# Mapping Reported Modes of Transmission of Highly Pathogenic Avian Influenza A (H5N1) to Humans: A Scoping Review

**DOI:** 10.1016/j.onehlt.2026.101492

**Published:** 2026-06-26

**Authors:** Nicole Billias, Victoria D’Alessandro, Dimitra V. Pouliopoulou, Jessica J. Wong, Erin Miller, Jessica P. Hopkins, Eleni C. Boutsikari, Lauren E. Cipriano, Tiago da Veiga Pereira, Jennie Johnstone, Saverio Stranges, J. Scott Weese, Joy C. MacDermid, Kieran L. Quinn, David N. Fisman, Pavlos Bobos

**Affiliations:** a School of Physical Therapy, Western University, London, Ontario, Canada; b Western’s Bone and Joint Institute, Western University, Canada; c Lawson Research Institute, St. Joseph’s Health Care London, London, Ontario, Canada; d Dalla Lana School of Public Health, University of Toronto, Toronto, Ontario, Canada; e Public Health Ontario, Ontario, Canada; f Department of Health Research Methods, Evidence, and Impact, McMaster University, Hamilton, Ontario, Canada; g Department of Preventive Medicine, Epidemiology & Medical Statistics, National and Kapodistrian University of Athens Medical School, Athens, Greece; h Richard Ivey School of Business, Western University, London, Ontario, Canada; i Nuffield Department of Population Health, University of Oxford, Oxford, UK; j Department of Medicine, University of Toronto, Toronto, Ontario, Canada; k Department of Epidemiology and Biostatistics, Schulich School of Medicine & Dentistry, Western University, London, Ontario, Canada; l Department of Family Medicine, Schulich School of Medicine & Dentistry, Western University, London, Ontario, Canada; m Department of Medicine, Schulich School of Medicine & Dentistry, Western University, London, Ontario, Canada; n Department of Clinical Medicine and Surgery, Federico II University, Naples, Italy; o Ontario Veterinary College, University of Guelph, Guelph, Ontario, Canada; p Temmy Latner Center for Palliative Care, Department of Medicine, Sinai Health, University of Toronto, Toronto, Ontario, Canada

**Keywords:** Avian Influenza, Influenza A, H5N1, Transmission

## Abstract

**Background:**

Highly Pathogenic Avian Influenza A (subtype H5N1) poses a threat to human health, and its pandemic potential emphasizes the need to better understand detailed reported transmission pathways to humans. Existing literature is outdated or lacks detailed, comprehensive analysis of the range of transmission routes and how the virus may enter the human body.

**Objective:**

To comprehensively map all reported H5N1 transmission pathways to humans, as well as viral entry routes.

**Methods:**

CINAHL, Embase, MEDLINE, Scopus, PubMed, grey literature, and reference lists (of included studies) were searched up to October 29th, 2025, with no language restrictions. Observational studies and grey literature reporting H5N1 transmission evidence to humans were included. Two reviewers conducted duplicate screening independently (two of three reviewers per record). One reviewer completed data extraction, which was cross-verified for accuracy by a second. Findings were summarized narratively.

**Results:**

120 sources met inclusion criteria (70 studies, 50 grey literature). Reported H5N1 transmission pathways were classified into animal-to-human (109 of 120 sources, 90.8%; including poultry-to-human in 100 sources [83.3%] and cattle-to-human in nine sources [7.5%]), environment-to-human (32 of 120 sources, 26.7%), and human-to-human (14 of 120 sources, 11.7%). Reported transmission pathways were further classified as direct or indirect contact, synthesized, and linked to suspected routes of human entry, including mucosal entry (eyes, nose, mouth), inhalation of aerosols or droplets, ingestion, and percutaneous exposure. Entry routes are biologically plausible and do not imply relative likelihood or causal attribution.

**Conclusions:**

There are multiple reported pathways of H5N1 exposure, and a single pathway may involve multiple ways to infect humans. Further research is needed to determine causal mechanisms, identify specific risk factors and measures of association, and strengthen evidence-based prevention strategies.

## 1 Introduction

Highly Pathogenic Avian Influenza A (HPAI), subtype H5N1, is a strain of avian influenza that encompasses multiple phylogenetic clades and poses a significant health threat to poultry and mammals worldwide [1]. This specific subtype of HPAI H5N1 is characterized by high pathogenicity and pandemic potential [2]. Occasional human infections have been reported primarily after exposure to infected poultry and cattle [1]. Clinical syndromes in humans include encephalitis, acute respiratory distress syndrome (ARDS), and respiratory and multiorgan failure [3]. Clinical presentations may include conjunctivitis, fever, malaise, cough, and gastrointestinal symptoms [3]. After exposure to infected avian species, the estimated incubation period in humans is approximately 3-5 days (range: ∼2-7 days), with a wider range observed in cases involving limited and clustered human-to-human transmission [3], [4], [5]. Since 2003, 993 human cases of H5N1 have been reported to the World Health Organization (WHO) across 25 countries and current data on human infections indicate a case fatality rate of approximately 48% [6], [7].

A recent WHO public health assessment of H5 viruses reports that current H5N1 strains would require genetic changes to spread efficiently between humans; therefore, the present public health risk remains low [8]. However, increasing H5N1 infection rates among mammals and widespread outbreaks in bird populations have raised global concerns about the virus’s ability to adapt, increasing the potential risk of human infection and its potential for zoonotic and pandemic spread should the virus mutate and spread more easily human-to-human [9], [10], [11]. This zoonotic risk emphasizes the importance of understanding current transmission pathways to humans to predict and prevent future outbreaks. An H5N1 pandemic in humans could severely strain healthcare systems, disrupt economies, and cause global public health emergencies.

Avian influenza viruses are primarily transmitted from birds to humans through direct or indirect contact with infected birds, their feces, or contaminated environments [2], [12], [13], [14]. Infected poultry excrete high viral loads in their feces, respiratory secretions, and saliva, all of which may be potential sources of human infection [14]. Respiratory particles may contribute to the spread of the virus (as demonstrated in animal models) [15], [16], [17], and inhalation of contaminated dust and debris particles may be a potential pathway of H5N1 infection in humans [18]. Influenza A viruses, including H5N1, replicate in epithelial cells of the lower and upper human respiratory tract [19]. The presence of α(2,3) receptors in the human eye suggests a potential ocular route of transmission, supported by reports of conjunctivitis in human avian influenza cases [20], [21]. Other studies have found that the virus may reach the nasopharynx via ocular pathways [22].

Epidemiological data shows that H5N1 is endemic among poultry populations in Asia [23], and that the risk of human infection is higher in settings with close human-animal contact and high-density animal farming [24]. People in rural or occupational settings (e.g., poultry workers, veterinarians, live bird market workers) have a higher risk of infection due to frequent exposure to infected birds and their secretions [25]. HPAI (subtype H5N1) can infect bovine populations [26] and may become endemic in dairy cattle (as observed in the United States [10], [27]) and may lead to human infection [28]. H5N1 viral RNA has been detected on environmental samples from live bird markets in Indonesia, as well as in mud, soil, and water in Cambodia, which suggests a potential source of indirect, environmental, and poultry-to-human transmission, and reinforces the importance of biosecurity measures [29], [30].

Given the pandemic potential of H5N1, there is an important need to understand detailed reported modes of H5N1 transmission to humans. Mapping all potential reported modes of transmission through a scoping review is an important first step in identifying and understanding all the different ways that the virus may be transmitted to humans. These findings can help guide future research on H5N1 transmission to humans (e.g., systematic reviews, studies of association), to improve risk assessment in high-risk populations, which in turn will help support effective prevention and control measures within a One Health framework [31]. There is available evidence on the transmission of other respiratory viral infections (such as seasonal influenza, H1N1, and SARS-CoV-2) [19], [32], [33]; however, to our knowledge, existing evidence syntheses are either outdated or lack a comprehensive analysis of the range of reported ways by which the virus enters the human body (based on reported modes of transmission) [34], [35], [36]. To address these gaps, a comprehensive scoping review was conducted to map all reported modes of transmission of H5N1 to humans, as well as associated pathways and entry routes of the virus, in humans with confirmed or suspected H5N1 infection.

## 2 Methods

### 2.1 Protocol & Registration

This scoping review uses the Joanna Briggs Institute guidelines for evidence synthesis (scoping reviews) [37] and a protocol was registered on Open Science Framework a priori at https://osf.io/c6d8a/ (10.17605/OSF.IO/C6D8A). This scoping review is reported in adherence to the Preferred Reporting Items for Systematic Reviews and Meta-Analyses extension for scoping reviews (PRISMA-ScR) [38]. This study was funded by the Canadian Institutes of Health Research (CIHR)’s Centre for Research on Pandemic Preparedness and Health Emergencies (CRPPHE) as part of a Catalyzing One Health Research on Avian Influenza funding opportunity (FRN: 196787).

### 2.2 Eligibility Criteria

Studies were eligible if they involved both human and animal populations relevant to H5N1 transmission to humans, from database inception to October 29th, 2025. Eligible study designs included observational studies (specifically, case reports or series, case-control, cohort, and cross-sectional studies), and relevant grey literature reporting on evidence of H5N1 transmission to humans, such as animal-to-human, environment-to-human, fomite-to-human, and human-to-human pathways. Reference lists of review articles were screened for relevant primary observational studies. Any relevant exposure pathways leading to human infection, and H5N1 entry routes in humans were extracted, if available.

The population of interest included humans of any age or sex with suspected or confirmed H5N1 infection. Studies were required to report human cases or exposures but could include animal populations when relevant to understanding transmission pathways to humans. Human-only studies were included if they reported H5N1 transmission evidence and details. Regarding context, studies conducted in settings where H5N1 transmission was observed (e.g., occupational, farm, healthcare, environment/community) were included. Concepts of interest included any form of transmission or epidemiological information or evidence on identified factors associated with H5N1 transmission to humans (i.e., direct contact with sick animals). There were no language restrictions in the search strategy and translation software [39], [40] was used to translate potentially relevant articles. There were also no restrictions on H5N1 pathogenicity, geographic location or time frame of included studies.

Qualitative studies, interventional studies (e.g., studies assessing antivirals, vaccinations, testing, personal protective equipment [PPE] interventions), commentaries, modelling studies, and editorials were excluded. Studies were also excluded if they did not explicitly specify H5N1 (e.g., H5 variants), did not report transmission or epidemiological information, or identified the source of H5N1 infection as unknown or under investigation.

### 2.3 Information Sources & Search Strategy

An electronic search strategy for the following databases was created and reviewed by a librarian: CINAHL, Embase, MEDLINE, Scopus, and PubMed. The search strategy (from inception to October 29th, 2025) used a combination of subject headings and free text terms pertaining to avian influenza, H5N1, transmission to humans, and observational studies. Additionally, reference lists from included studies were manually searched at the title level to identify studies not captured in the electronic search. In terms of grey literature, the search was informed by the University of Toronto’s Guide to Comprehensive Searches in the Health Sciences [41]. A reference list from a recent report [8] from the World Health Organization (WHO) was searched, as well as individual reported summaries of case investigations via the WHO’s Disease Outbreak News (DONs) from 2004 to 2025 were manually searched. A general web search was conducted to identify government and news sources reporting transmission or epidemiological information for countries with confirmed human H5N1 cases reported to the WHO [42]. Case investigation news articles from the Center for Infectious Disease Research & Policy Research and Innovation Office (University of Minnesota), U.S. Centers for Disease Control and Prevention (CDC) Newsroom, Wisconsin Department of Health Services, Iowa Department of Health and Human Services, Central Nevada Health District, Ohio Department of Health, and the Pan American Health Organization were searched and included to find details on cases of H5N1 transmission to humans. The supplement file summarizes the search strategy, including the grey literature search.

### 2.4 Study Selection

All records obtained via the electronic search strategy were imported into Covidence [43]. After automatic removal of duplicate records, 25 randomly selected studies were pilot screened using the set study eligibility criteria, to ensure consistency among three independent reviewers (NB, VD, and DVP, two of three reviewers per record). After calculating percentage agreement, the reviewers discussed discrepancies to refine the screening approach, and then independently conducted title and abstract, and full-text screening in duplicate. Conflicts during screening were resolved through consensus via verbal discussion, and a senior team member (PB) was consulted for conflicts at the full-text level. All studies that met the eligibility criteria were then included for data extraction.

### 2.5 Data Charting Process & Data Items

One reviewer (NB) independently completed data extraction using a Microsoft Excel spreadsheet, which was cross-verified by another (VD) for accuracy. To guide extraction of relevant information from the included literature, a standardized form was developed and piloted. The following information was extracted: study identification information (author[s], year, title, journal/source, country/region), study characteristics (study design, overall study/literature setting [e.g., healthcare facility-based], duration, sample size), population (human subgroups, age, number of males and females), reported exposure type, setting(s) (specific exposure environment where participants encountered H5N1 (e.g., backyard poultry, live bird markets), outcomes (reported transmission mode, suspected detailed transmission, suspected viral entry route, testing/methods of laboratory confirmation, serological evidence, and risk and protective factors described), detection methods (case definition, pathogenicity type), and funding source(s). All information was extracted exactly as reported and missing data was marked as “Not Reported.” Unclear or ambiguous data were flagged for group discussion. See the supplement file for the standardized form.

### 2.6 Data Synthesis

Narrative analysis and synthesis were used to summarize suspected reported detailed transmission of H5N1 to humans (e.g., specific pathways that led to the virus infecting humans). Categorical variables were summarized as frequencies and percentages (e.g., country, study design, year of publication, study population, setting[s], study setting, methods of testing confirmation, serological evidence). Transmission pathways synthesized in this review are informed by evidence with varying levels of diagnostic and epidemiologic confirmation reported in the literature, such as polymerase chain reaction (PCR) or viral isolation/sequencing (confirming active infection), serological evidence (indicating prior exposure without a defined infection window), and detailed outbreak investigations or grey literature reports (that may rely on epidemiologic linkage without biological confirmation).

Modes of reported H5N1 transmission to humans were classified into three categories: (1) animal-to-human, (2) environment-to-human, and (3) human-to-human. Animal-to-human transmission refers to human infections reported to be linked to direct or indirect contact with infected animals (poultry or cattle). Environment-to-human transmission is a subtype of indirect transmission and, in the context of this paper, refers to human infections associated with reported exposure to suspected contaminated environmental reservoirs [44] where viral survival/persistence may occur (e.g., mud, dust, dirt, water, milk and milk-derived aerosols, and aerosolized particles from infected animal secretions) [29], [30], [45], [46] in settings where infected or potentially infected avian populations, cattle, or their products were present. H5N1 has been detected in environmental samples such as water, dust, mud and soil following outbreaks [47], and these have been suggested as potential sources of human infection [48]. Human-to-human transmission refers to cases reported and suspected to be resulting from direct or indirect contact with a human H5N1 case.

Within the animal-to-human and human-to-human categories, transmission was further classified as direct or indirect contact. Direct contact includes physical handling or touching of animals, animal products (e.g., feathers, organs, uncooked meat), or H5N1 patients and their bodily secretions (e.g., respiratory secretions, bathing), or inhalation of close-range droplets, as reported in the literature. Indirect contact refers to reported exposure through proximity, contaminated surfaces, shared spaces without direct contact (e.g., no patient care, no exposure to respiratory particles), food products, without direct handling or touching, as well as no exposure to contaminated environmental reservoirs.

Pathways of H5N1 transmission to humans were synthesized to describe (within the categories of animal-to-human, environment-to-human, and human-to-human transmission) reported exposure scenarios and biologically plausible entry routes, without implying relative likelihood or causal attribution. These included mucosal entry via the eyes, nose, or mouth (120 of 120 sources, 100%), inhalation of aerosols or close-range droplets (117 sources, 97.5%), ingestion (20 sources, 16.7%), and percutaneous exposure (one source, 0.8%). The figures illustrating transmission pathways were developed iteratively as recurring pathways were identified and refined to align with the current understanding of avian influenza A virus transmission and persistence [18]. Because sources contributing to these pathway frequencies span this range of diagnostic certainty, reported counts reflect the frequency with which a pathway was reported across the literature, not confirmed transmission events. Pathway inferences are most robust where they derive from molecularly confirmed, acute cases with a clear temporal link to the reported exposure, and are weakest where they rest on serological or seroprevalence evidence in which exposure timing is undefined.

## 3 Results

### 3.1 Selection of Evidence Sources

**Fig. 1:**
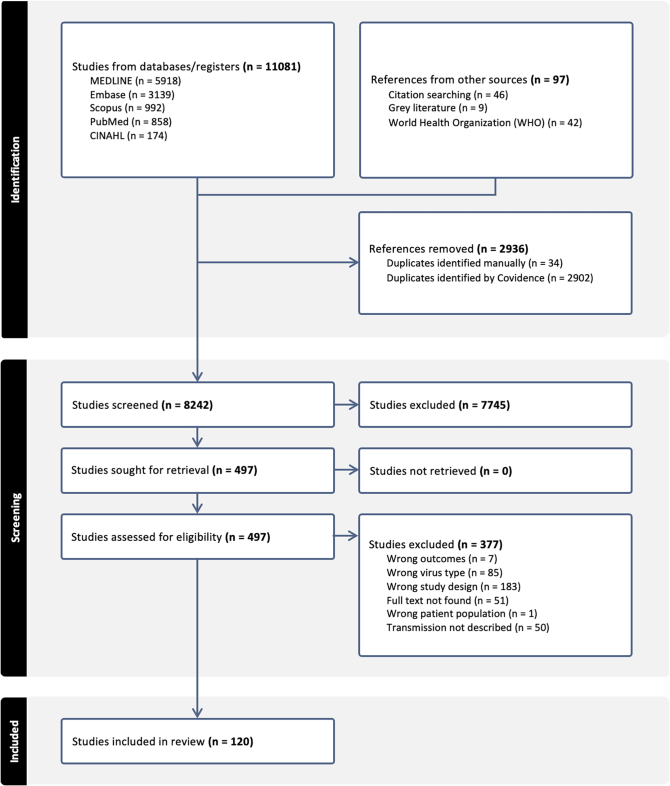
PRISMA flow chart.

After completing the search strategy, a total of 11,178 records were imported into Covidence. After automatic duplicate removal, 8242 records were screened at the title and abstract level, followed by full-text screening, with moderate inter-reviewer agreement. Overall, 120 literature sources were included in this review (70 studies and 50 grey literature sources). The PRISMA flow-chart (Fig. 1) outlines the screening process [49].

### 3.2 Characteristics of Evidence Sources

Table 1 summarizes the characteristics of the included literature. The majority originated from Asia (n = 86 of 120 sources, 72.0%), which included Azerbaijan (n = one, 0.8%), Bangladesh (n = four, 3.3%), Cambodia (n = 12, 10.0%), China (n = 19, 15.8%), India (n = one, 0.8%), Indonesia (n = 12, 10.0%), Iraq (n = one, 0.8%), Laos (n = one, 0.8%), Myanmar (n = one, 0.8%), Pakistan (n = one, 0.8%), South Korea (n = one, 0.8%), Thailand (n = 14, 11.7%), Türkiye (n = three, 2.5%), and Vietnam (n = 15, 12.5%). Of the 120 sources, two were from South America (Chile [n = one, 0.8%] and Ecuador [n = one, 0.8%]), one from Russia (0.8%), 12 (10.0%) from Africa (Cameroon [n = one, 0.8%], Egypt [n = nine, 7.5%], and Nigeria [n = two, 1.7%]), one (0.8%) from Spain, two (1.7%) from the United Kingdom (England), and 16 (13.3%) from the United States.

**Table 1:** Study and population characteristics of 120 included literature sources with reported modes of transmission of H5N1 to human populations.

Characteristic	Category	Number of Studies	%
**Country**	Azerbaijan	1	0.8%
	Bangladesh	4	3.3%
	Cambodia	12	10.0%
	Cameroon	1	0.8%
	Chile	1	0.8%
	China	19	15.8%
	Ecuador	1	0.8%
	Egypt	9	7.5%
	England	2	1.7%
	India	1	0.8%
	Indonesia	12	10.0%
	Iraq	1	0.8%
	Laos	1	0.8%
	Myanmar	1	0.8%
	Nigeria	2	1.7%
	Pakistan	1	0.8%
	Russia	1	0.8%
	South Korea	1	0.8%
	Spain	1	0.8%
	Thailand	14	11.7%
	Türkiye	3	2.5%
	United States	16	13.3%
	Vietnam	15	12.5%
**Literature Type**	Case Investigation or Series	25	20.8%
	Case Report	13	10.8%
	Case-Control	5	4.2%
	Cohort studies (prospective or historical)	13	10.8%
	Cross-sectional studies	14	11.7%
	News Release	8	6.7%
	WHO Press Release	41	34.2%
	Report	1	0.8%
**Year of Publication**	1999-2008	49	40.8%
	2009-2017	36	30.0%
	2018-2025	35	29.2%
**Age**	Literature that reported means (pooled mean: 23.9 years)	7	5.8%
	Literature that reported medians (mean of medians: 23.2 years; range of ranges: 1 to 77 years)	28	23.3%
	Literature that reported single age (median: 21.5 years, range: 9 months–59 years)	57	47.5%
	Literature that reported age categories	6	5.0%
	Literature that did not report age	22	18.3%
**Sex**	Males	527	44.1%
	Females	462	38.7%
	Not Reported	205	17.2%
**Reported H5N1 Case Type**	Confirmed	873	73.1%
	Suspected	321	26.9%
**Sample Size** (only suspected or confirmed cases, and/or seropositive)	1	51	42.5%
	2-25	53	44.2%
	26-50	10	8.3%
	51+	6	5.0%
**Literature Setting**	Agriculture	26	21.7%
	Healthcare	25	20.8%
	Veterinary	1	0.8%
	Rural Areas	19	15.8%
	Urban Areas	37	30.8%
	Rural and Urban Areas	4	3.3%
	1+ Category	4	3.3%
	Not Reported	4	3.3%
**Population** (reported in literature, may report more than one population)	Backyard Poultry Owners/Poultry Consumers	62	51.7%
	Residents of Poultry-Contaminated Environments	32	26.7%
	Close Contacts of H5N1 Cases	13	10.8%
	Commercial Poultry Facility Workers	19	15.8%
	Live Bird Market Workers	10	8.3%
	Dairy Workers	9	7.5%
	Swine Workers	2	1.7%
	Healthcare Workers	1	0.8%
	Live Bird Market Visitors	13	10.8%
	Hunters	1	0.8%
	Veterinary Professionals	1	0.8%
	Gardener	2	1.7%
	Not Reported	13	10.8%
**Participant Exposure Setting** (reported in literature, may report more than one setting)	Backyard/Neighbourhood Poultry	55	45.8%
	Live Bird Markets	24	20.0%
	Commercial Poultry Facilities	21	17.5%
	Commercial Swine Facilities	2	1.7%
	Commercial Beef Facilities	1	0.8%
	Dairy Farms	8	6.7%
	Poultry-Contaminated Environments	31	25.8%
	Poultry Preparation Environments	21	17.5%
	Healthcare/Bedside Care	10	8.3%
	Veterinary Care	1	0.8%
	Gardens	2	1.7%
	Hunting/Cockfighting Environments	6	5.0%
	Not Reported	13	10.8%
**Reported Transmission Mode** (literature may report more than one)	Poultry-to-Human	100	83.3%
	Cattle-to-Human	9	7.5%
	Environment-to-Human	32	26.7%
	Human-to-Human	14	11.7%
**Suspected Entry Route** (literature may report more than one)	Mucosa (oral/nasal/conjunctival)	120	100.0%
	Inhalation (respiratory)	117	97.5%
	Gastrointestinal	20	16.7%
	Percutaneous	1	0.8%
**Testing/Methods of Transmission Confirmation** (literature may use more than one)	Polymerase chain reaction (PCR) (not specified)	4	3.3%
	RT-PCR	48	40.0%
	qRT-PCR	2	1.7%
	Microneutralization Assay	31	25.8%
	Hemagglutination Inhibition Assay	24	20.0%
	Viral Culture or Isolation	11	9.2%
	Enzyme-linked Immunosorbent Assay (ELISA)	3	2.5%
	Used confirmatory Western blot assay	11	9.2%
	Genetic/genome or Molecular Sequencing	21	17.5%
	Antiviral Resistance Test	1	0.8%
	Antigen Detection	1	0.8%
	Rapid Influenza Test	1	0.8%
	Not Reported	37	30.8%
**Serological Evidence**	Reported	40	33.3%
	Not Reported	80	66.7%
**Risk & Protective Factors Described**	Reported	33	27.5%
	Not Reported	87	72.5%

In terms of literature type, most were research studies (case investigations or series [n = 25 of 120 sources, 20.8%], cross-sectional studies [n = 14, 11.7%], prospective or historical cohort studies [n = 13, 10.8%], case reports [n = 13, 10.8%], and case-control studies [n = five, 4.2%]). Regarding grey literature sources, news releases (n = eight of 120 sources, 6.7%), WHO press releases (n = 41, 34.2%), and a report (n = one, 0.8%) were included. Most literature was published between 1999 and 2008 (n = 49 of 120 sources, 40.8%), followed by 2009-2017 (n = 36, 30.0%) and 2018-2025 (n = 35, 29.2%).

### 3.3 Patient/Participant Characteristics

Age was variably reported across the literature sources. Approximately half of the sources (n = 57 of 120, 47.5%) reported single ages in years, with a median age of 21.5 years (range: 9 months to 59 years). Medians were reported in 28 of 120 sources (23.3%), with the mean of the medians being 23.2 years, and the range of the ranges spanning one to 77 years. Means were reported in seven of 120 sources (5.8%), with the pooled mean being 23.9 years. Categorical/interval age groups were used in six of 120 sources (5.0%) and categories varied widely (e.g., ranging from 0 to 4 years to 63-78 years). Finally, 22 of 120 sources (18.3%) did not report age.

A total of 1194 people were identified in the included literature, comprising 873 confirmed (73.1%) and 321 suspected (26.9%) cases. Here, “confirmed” encompasses two forms of laboratory evidence that differ in what they establish about the timing of infection: molecular confirmation of acute infection (e.g., RT-PCR, viral isolation or sequencing) and serological confirmation of prior exposure (e.g., microneutralization, hemagglutination inhibition, or ELISA), the latter indicating past infection of undefined timing. This distinction is relevant to interpreting the transmission pathways described below, as serologically defined cases cannot establish a temporal link between a specific reported exposure and acute infection. Of these people, 527 were male (44.1%), 462 were female (38.7%), and sex was not reported for 205 people (17.2%). Nearly half of the sources (n = 53 of 120, 44.2%) included two to 25 people, while 51 sources (42.5%) described a single person. Ten of 120 sources (8.3%) included 26 to 50 people, and six sources (5.0%) reported on more than 51 people.

### 3.4 Setting (Literature)

Most of the included literature took place in urban areas (e.g., cities, regions, suburbs) (37 of 120 sources, 30.8%), followed by agricultural settings (26 sources, 21.7%) and healthcare (25 sources, 20.8%), and rural areas (e.g., villages) (19 sources, 15.8%). Fewer literature sources involved both urban and rural areas, multiple settings, unspecified settings (four sources each, all 3.3%), or veterinary settings (one source, 0.8%).

### 3.5 Setting (Participant Exposure)

Regarding the specific settings where people were exposed, the most common was backyard or neighbourhood poultry (defined as poultry owned by people in a home or neighbourhood setting) (55 of 120 sources, 45.8%). Other settings included poultry-contaminated environments (31 of 120 sources, 25.8%), live bird markets (24 sources, 20.0%), commercial poultry facilities (21 sources, 17.5%), poultry preparation/processing environments (e.g., slaughtering, defeathering, disemboweling) (21 sources, 17.5%), healthcare/bedside care (10 sources, 8.3%), dairy farms (eight sources, 6.7%), hunting/cockfighting environments (six sources, 5.0%), commercial swine facilities (two sources, 1.7%), gardens (two sources, 1.7%), commercial beef facilities (one source, 0.8%), and veterinary facilities (one source, 0.8%). Thirteen sources (10.8%) did not specify settings.

The most commonly affected population infected or exposed to H5N1 were backyard poultry owners and consumers (62 of 120 sources, 51.7%), followed by residents of poultry-contaminated environments (those that reported living in neighbourhoods with sick or dead poultry) (32 sources, 26.7%), commercial poultry facility workers (19 sources, 15.8%), close contacts of human H5N1 cases (13 sources, 10.8%), live bird market visitors (13 sources, 10.8%), live bird market workers (10 sources, 8.3%), dairy workers (nine sources, 7.5%), gardeners (two sources, 1.7%), swine workers (two sources, 1.7%), and healthcare workers, hunters, and veterinary professionals (each reported in one source, all 0.8%). In 13 sources (10.8%), the population was not specified.

Laboratory confirmation of H5N1 (in both surveillance and acute clinical settings) was as follows for the suspected (321 cases) or confirmed (873 cases): reverse transcriptase polymerase chain reaction (RT-PCR), the current gold standard for testing avian influenza viruses [14], was reported in 48 of 120 (40.0%) of sources. Two of 120 (1.7%) of sources specifically reported using qRT-PCR, and four (3.3%) reported using PCR but did not specify which type. Other reported laboratory confirmation methods included microneutralization assays (31 of 120 sources, 25.8%), hemagglutination inhibition assays (24 sources, 20.0%), genetic/genome or molecular sequencing (21 sources, 17.5%), viral culture or isolation (11 sources, 9.2%), and enzyme-linked immunosorbent assay (ELISA) (three sources, 2.5%), with antiviral resistance testing, antigen detection, and rapid influenza tests each accounting for one source (0.8%). A confirmatory Western blot assay, when combined with microneutralization assays (the gold standard for detecting anti-H5N1 antibodies in humans) [50], was used in 11 of 120 sources (9.2%). Thirty-seven of 120 sources (30.8%) did not specify the testing method.

In addition to laboratory confirmation, 40 of 120 sources (33.3%) reported collecting serological evidence of H5N1 exposure (including hemagglutination inhibition assays, microneutralization assays, or ELISA testing), and 33 of 120 sources (27.5%) described epidemiological risk and/or protective factors (e.g., exposure duration, vaccination status, oseltamivir administration [to prevent or treat infection], use of, and compliance with, PPE).

### 3.6 Synthesis of Results: Methods of H5N1 Transmission to Humans

#### 3.6.1 Animal-to-Human

**Fig. 2:**
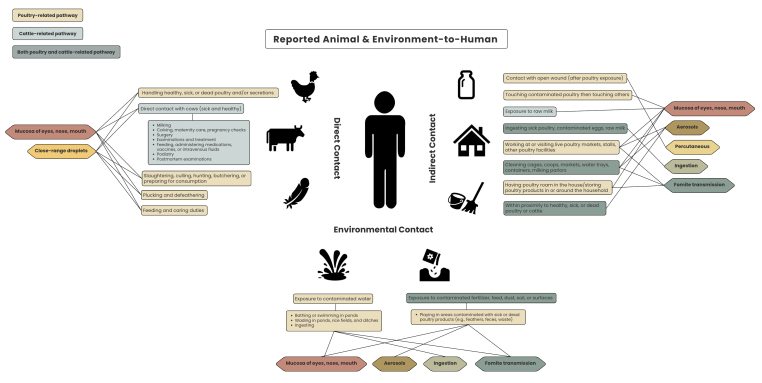
Reported suspected animal and environment-to-human transmission pathways of H5N1 and corresponding viral entry routes. Yellow pathways indicate poultry-related transmission, light blue indicates cattle-related transmission, and dark blue indicates pathways shared between poultry and cattle. Lines linking pathways to entry routes (mucosa, close-range droplets, aerosols, percutaneous, ingestion, fomites) demonstrate that a single pathway may involve multiple entry routes and therefore more than one way of infecting humans. (For interpretation of the references to colour in this figure legend, the reader is referred to the web version of this article.)

**Fig. 3:**
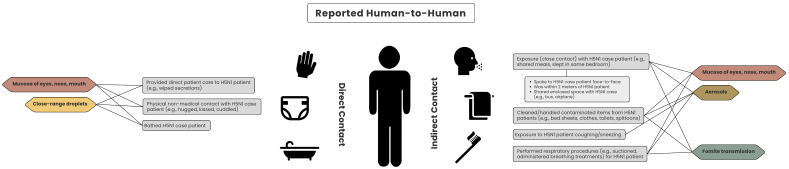
Reported suspected human-to-human transmission pathways of H5N1 and corresponding viral entry routes.

Fig. 2, Fig. 3 provide a visual representation of transmission pathways. One hundred and nine of 120 sources (90.8%) reported contact history or epidemiological information on animal-to-human transmission, including poultry-to-human (100 of 120 sources, 83.3%) and cattle-to-human (nine of 120 sources, 7.5%). Within this category, five pathways were classified as *direct contact* and linked to their respective entry routes (see Fig. 2). The first pathway involves contact with or handling of healthy, sick, or dead poultry and/or their secretions, reported in 69 of the 100 sources (69.0%) describing poultry-to-human transmission [51], [52], [53], [54], [55], [56], [57], [58], [59], [60], [61], [62], [63], [64], [65], [66], [67], [68], [69], [70], [71], [72], [73], [74], [75], [76], [77], [78], [79], [80], [81], [82], [83], [84], [85], [86], [87], [88], [89], [90], [91], [92], [93], [94], [95], [96], [97], [98], [99], [100], [101], [102], [103], [104], [105], [106], [107], [108], [109], [110], [111], [112], [113], [114], [115], [116], [117], [118], [119]. General exposure to sick and/or dead poultry was stated in 20 of 100 sources (20.0%) [74], [76], [79], [110], [120], [121], [122], [123], [124], [125], [126], [127], [128], [129], [130], [131], [132], [133], [134], [135], as well as exposure to pigeons in one of 100 sources (1.0%) [107]. General exposure to poultry (not specifying poultry health) was stated in seven of 100 sources (7.0%) that reported poultry-to-human transmission [76], [95], [107], [134], [136], [137], [138]. Notably, four of 100 (4.0%) sources reported lack of hand hygiene (handwashing) [87], [108], [117], [139] eight (8.0%) reported lack of PPE use [61], [86], [89], [108], [110], [117], [118], [140], one (1.0%) reported general poor hygiene conditions while working with poultry [141], and one (1.0%) reported that longer daily exposure to poultry led to positive antibody titres [80], all of which are factors that may have facilitated poultry-to-human transmission.

The second pathway involves direct contact with dairy cattle (both sick and healthy), all of which are cases reported from the United States [10], [27]. The following activities were reported in seven of nine sources (77.8%) reporting cattle-to-human transmission, that possibly lead to human infection with H5N1 among veterinary professionals and dairy workers: milking, calving and maternity care, surgery, examinations and treatment, feeding, administering medications, vaccines, or intravenous fluids, podiatry, pregnancy checks, postmortem examinations, and working in close proximity to cattle [92], [142], [143], [144], [145], [146], [147]. General exposure to cattle was stated in two of nine sources that reported cattle-to-human transmission (22.2%) [148], [149]. One study (of nine, 11.1%) reported that an H5N1-positive worker was splashed in the eye while milking a cow on a farm with confirmed dairy cattle H5N1 cases, after which the worker experienced eye discomfort [145]. Studies that reported risk and protective factors among people who had direct contact with cattle report low or varying compliance with PPE, which may have facilitated transmission to humans. Respiratory and eye protection were often used infrequently or not at all, whereas gloves were more commonly used (five of nine sources, 55.6%) [92], [143], [144], [145], [146]. Some studies reported lack of respiratory protection and masking, and some use of eye protection and gloves (two of nine sources, 22.2%) [144], [145]. Another study (one of nine sources, 11.1%) found that although most workers (78.0%) reported using some form of PPE, compliance varied across items, with specifically low compliance among gowns, boots, and non-respirators (e.g., face coverings or masks) [147]. One study (of nine, 11.1%) stated that none of the dairy workers with H5N1 antibodies reported following recommended PPE guidelines for working with H5N1-infected animals [144].

The third reported direct contact pathway is slaughtering, culling, hunting, butchering, or preparing poultry for consumption (handling some form of raw meat or viscera) (42 of 100 sources reporting poultry-to-human transmission, 42.0%) [[52], [54], [60], [61], [62], [69], [70], [71], [73], [75], [76], [77], [79], [80], [81], [82], [85], [87], [88], [89], [90], [91], [92], [93], [97], [99], [101], [102], [103], [104], [108], [109], [112], [113], [114], [115], [116], [117], [137], [139], [141], [150]]. One study (of 100 sources, 1.0%) reported that “more intensive” poultry exposure (including butchering) and a higher number of reported poultry exposures were associated with workers testing positive for H5N1 antibodies [82].

The fourth pathway is plucking and defeathering poultry (with or without handling some form of raw meat or viscera, reported in 12 of 100 sources reporting poultry-to-human transmission, 12.0%) [60], [81], [85], [87], [93], [97], [108], [111], [112], [115], [116], [117]. The fifth and final direct contact pathway reported in the literature is feeding and caring for poultry (seven of 100 sources, 7.0%) [[54], [82], [108], [114], [116], [118], [151]]. One source (of 100, 1.0%) from Türkiye reported that during the winter months, poultry were kept indoors (when usually outdoors) in cramped household living conditions, increasing human-poultry contact and, as a result, the risk of transmission [54].

Eight reported *indirect contact* pathways were identified that may facilitate H5N1 infection in humans, each linked in some way to one or more of the entry routes into the human body. Suspected pathways reported to cause infection via the mucosa of the eyes, nose, or mouth included (1) touching suspected infectious poultry/contaminated poultry meat and then touching other humans (two of 109 sources reporting animal-to-human transmission, 1.8%) [87], [139], (2) exposure to raw milk (two of 109 sources, 1.8%) [92], [144], (3) cleaning cages, coops, markets, water trays, containers, or milking parlours (nine of 109 sources, 8.3%) [[82], [85], [91], [108], [112], [117], [118], [144], [151]], and (4) having poultry roam in the house, and/or storing suspected sick or dead poultry materials/products in or around the home (11 of 109 sources, 10.1%) [[81], [86], [87], [111], [116], [119], [135], [139], [141], [152], [153]]. General indirect exposure to sick and/or dead poultry was explicitly stated in one source (one of 109 sources, 0.9%) [79], while another source defined indirect contact as poultry presence within 25 m of a confirmed case’s house (one of 109 sources, 0.9%) [113]. Poor hand hygiene after handling contaminated poultry during slaughtering then touching another person (reported in two of 109 sources, 1.8%) [87], [139] could have led to self-inoculation and subsequent suspected infection. In occupational settings (specifically, dairy workers), touching raw milk followed by self-inoculation (e.g., contact with the eyes, nose, or mouth) was specifically reported in one of 109 sources (0.9%) as a possible mode of H5N1 transmission to humans [92].

Tying into fomite transmission and self-inoculation, working at or visiting live poultry markets, stalls, or other poultry facilities (38 of 109 sources, 34.9%) [[61], [71], [73], [77], [79], [81], [82], [85], [90], [91], [92], [94], [95], [96], [99], [101], [102], [105], [107], [108], [109], [112], [113], [116], [118], [119], [121], [122], [133], [137], [138], [140], [141], [151], [154], [155], [156], [157]], as well as cleaning cages, coops, markets, water trays, containers, or milking parlours are reported pathways that may lead to infection through contact with these contaminated surfaces themselves (e.g., high touch surfaces, cages), as well as objects coming in contact with these surfaces (e.g., cleaning supplies). One (of 109 sources, 0.9%) reported cleaning a poultry house without the use of PPE [118], which could have facilitated self-inoculation or inhalation of infectious aerosolized particles.

Less frequently reported possible transmission pathways of H5N1 included ingestion and percutaneous infection. Ingestion of the virus through possibly contaminated, raw/undercooked poultry or wild birds, raw milk, or eggs was reported as a possible indirect transmission pathway in 17 of 109 sources (15.6%) [[54], [62], [69], [70], [85], [86], [89], [92], [93], [101], [103], [106], [108], [114], [115], [116], [153]]. Reported cases included people who consumed poultry or wild birds suspected of being infected with H5N1 (16 of 109 sources, 14.7%) [[54], [62], [69], [70], [85], [86], [89], [93], [101], [103], [106], [108], [114], [115], [116], [153]], as well as those who ate undercooked eggs (two of 109 sources, 1.8%) [108], [116] or possibly consumed raw milk (one of 109 sources, 0.9%) [92]. Only one source (of 109 sources, 0.9%) [87] reported H5N1 infection after poultry exposure involving contact with an open wound (in addition to direct hand contact with infected poultry), suggesting a possible (though highly unlikely) percutaneous route of viral entry via the skin.

#### 3.6.2 Environment-to-Human

Thirty-two of 120 sources (26.7%) reported contact history or epidemiological information on possible environment-to-human transmission of H5N1. General environmental exposure was reported in two of 32 sources (6.3%) [[158], [159]].

One potential poultry-related transmission pathway (reported in seven of 32 sources, 21.9%) is exposure to contaminated water [[84], [85], [106], [117], [141], [160], [161]]. Reported behaviours included bathing or swimming in ponds (three of 32 sources, 9.4%) [[85], [117], [160]] (specifically reported to be inhabited by poultry, wild waterfowl and/or ducks [84], [106]), wading in ponds, rice fields, and ditches (one source, 3.1%) [141], and consuming water possibly contaminated by poultry (two sources, 6.3%) [[160], [161]]. Transmission to humans may occur through ingestion of contaminated drinking water, accidental swallowing while swimming in ponds or canals contaminated with H5N1, or via fomites that have come into contact with infected water [48]. People without access to an indoor water source were reported to have a higher risk of H5N1 exposure (compared to those with an indoor water source [161]), as well as those who waded in ponds, rice fields, and ditches [141] (as reported in two sources, 6.3%). Additionally, swimming or washing in contaminated areas (e.g., canals, ponds) may facilitate viral inoculation through the mucosa of the eyes or nose [48].

Reported possible exposure to fertilizer, feed, dust, or soil suspected to be contaminated with poultry or cattle feces, mucus, or saliva (e.g., poultry deaths in the neighbourhood), may be a potential transmission pathway (27 of 32 sources, 84.4%) [[5], [52], [54], [62], [82], [85], [88], [89], [94], [98], [101], [106], [108], [110], [112], [113], [116], [117], [126], [141], [151], [162], [163], [164], [165], [166], [167]]. One source (of 32, 3.1%) reports that a case who used chicken feces garden fertilizer (that later tested positive for H5N1) did not use gloves or a mask while gardening, which could have facilitated transmission [162]. Another source (one of 32, 3.1%) reports a fish farmer that used a feeder containing dried bird feces [101], while another reports possible transmission from fertilizers containing chicken manure [94]. Specifically, in young children, playing in areas contaminated with sick or dead poultry products (e.g., feathers, feces, waste) has been reported as a behaviour possibly leading to exposure and infection (four of 32 sources, 12.5%) [[5], [88], [89], [163]].

#### 3.6.3 Suspected Human-to-Human

Limited, inefficient, non-sustained, suspected human-to-human transmission was observed in the 14 of 120 sources (11.7%) [[5], [52], [61], [79], [81], [83], [98], [99], [113], [114], [134], [136], [168], [169]] that reported human exposure leading to possible human-to-human transmission of H5N1. This means that transmission has only been detected in rare instances, it requires specific conditions to spread between people (e.g., very close-contact situations such as family caregiving, prolonged exposure), and once it passes from one person to another, the virus is unlikely to continue to spread further. Pathways within this category were further classified as either *direct* or *indirect* contact and linked to the corresponding suspected entry route(s) into the human body.

Reported *direct contact* pathways for suspected human-to-human transmission included providing patient care to an H5N1 case (e.g., wiping secretions) (eight of 14 sources, 57.1%) [[5], [52], [61], [83], [99], [136], [168], [169]], having physical, non-medical contact with the case (e.g., hugging, kissing, cuddling) (two sources, 14.3%) [5], [99], and bathing the case patient (one source, 7.1%) [136]. A few studies reported general human contact (three of 14 sources, 21.4%) [79], [81], [114], close contact (one source, 7.1%) [98], and exposure (three sources, 21.4%) [79], [134], [136] to human H5N1 cases (suspected or confirmed) as transmission pathways. Healthcare workers who later tested H5N1 antibody-positive had followed standard infection-control procedures; however, droplet precautions (e.g., masks, gloves, gowns, and eye protection within 3 ft of the patient) were not used because the H5N1 infection was identified only after the patient’s death (as reported in one source, 7.1%) [136]. Other literature sources reported either a lack of PPE use during close contact and bedside care of H5N1-positive cases (two sources, 14.3%) [[5], [168]] or delayed use of PPE (e.g., surgical masks, gloves, gowns, hair covers, N95 respirators, eye protection) after the infection was confirmed (one source, 7.1%) [169].

Reported *indirect contact* for suspected human-to-human transmission pathways included exposure (close contact without direct touching) to an H5N1 case patient (e.g., caregiving, shared meals, slept in same bedroom) (14 of 14 sources, 100%) [[5], [52], [61], [79], [81], [83], [98], [99], [113], [114], [134], [136], [168], [169]]. As sub-pathways within this pathway, speaking face-to-face (two sources, 14.3%) [99], [136], being within two meters of an H5N1 case patient (11 of 14 sources, 78.6%) [[5], [61], [79], [83], [98], [99], [113], [134], [136], [168], [169]], and sharing enclosed spaces with infected H5N1 cases (e.g., buses, airplanes) (one source, 7.1%) [99] were reported as possible routes of transmission. Additional reported indirect transmission pathways included cleaning or handling contaminated items from H5N1 patients (e.g., bed sheets, clothing, toilets, spittoons) (two of 14 sources, 14.3%) [[136], [169]], exposure to coughing or sneezing (four sources, 28.6%) [[99], [136], [168], [169]], performing respiratory procedures (e.g., suctioning, administering breathing treatments) (one source, 7.1%) [136]. It is important to note that there is likely overlap between indirect and direct contact pathways and distinguishing between them is not always possible. For example, speaking face-to-face with an H5N1 case patient, exposure to coughing or sneezing, and performing respiratory procedures can lead to exposure to both infectious droplets (direct contact) and aerosols (indirect contact) [170] that may be inhaled. Additionally, respiratory procedures often involve both direct contact with the patient and indirect contact with fomites (e.g., contaminated medical instruments). Finally, many reported human-to-human transmission events occurred in settings with overlapping animal or environmental exposures. Therefore, it is difficult to rule out common-source transmission rather than true, limited, human-to-human spread.

## 4 Discussion

This review used a structured approach to map all plausible reported modes of H5N1 transmission to humans, including associated pathways and viral entry routes. Importantly, entry routes are biologically plausible and do not imply relative likelihood or causal attribution. Findings suggest that there are likely multiple pathways of exposure based on reported transmission in the literature, and a single pathway may involve more than one entry route (and therefore multiple ways) to infect humans.

The most commonly reported exposure pathway was handling healthy, sick, or dead poultry and their secretions, which is consistent with one of the most commonly reported exposures in human H5N1 cases [171]. This finding may be due to public health challenges caused by direct contact with infected or contaminated poultry, specifically in regions where backyard or small-scale poultry farming is common, and education/awareness on biosecurity may be limited. Infected poultry shed large amounts of virus through droppings, respiratory secretions, and saliva, all of which can contaminate surfaces and the environment, and can enable many routes of human exposure and infection [14]. To add, lack of hand hygiene (handwashing) and PPE were reported in a few studies, which could have facilitated transmission of the virus to humans. Humans may become infected through contact with clinically normal (yet infectious), sick, or dead poultry or with contaminated secretions (e.g., feces, blood, saliva). Transmission can occur via self-inoculation of the eyes, nose, or mouth after handling birds, tissues, or contaminated materials, or through splashes of infectious fluids to the mucosa during activities such as slaughtering or food preparation. Infection may also occur through inhalation of infectious droplets or aerosols generated at close range during poultry handling or processing, from animals’ respiratory aerosols, or from activities that disturb or disperse possible contaminated materials (e.g., sweeping feces, dirt, or hay, laying down fertilizer, using spray bottles while cleaning). Similarly, having poultry roam in the house and storing poultry products in or around the household can contaminate objects and facilitate self-inoculation. The CDC recommends that people avoid unprotected exposure to sick or dead animals and their secretions, and use appropriate PPE (safety goggles, N95 respirators, coveralls, disposable gloves, rubber boots) when in close proximity to potential infection sources [172]. For occupational settings, such as poultry, cattle, and healthcare facilities (where healthcare workers may be exposed to H5N1 case patients), the CDC recommends that employers provide PPE, ensure training in proper use, and reinforce infection control protocols [[172], [173], [174]].

Consumption of possibly undercooked poultry meat is another suspected transmission route that was reported. Although properly cooked meat and eggs pose minimal risk [175], consumption of raw or undercooked poultry or eggs may facilitate infection [176]. Sources that report ingestion of poultry could reflect detailed epidemiological documentation/investigation rather than gastrointestinal transmission. Additionally, unpasteurized milk consumption (ingestion) and exposure (possible self-inoculation) were found to be reported suspected transmission pathways. Milk from infected cattle has a high viral load and has been shown to be infectious when ingested by other mammals [[177], [178], [179], [180]]. H5N1 has also been detected on milking equipment, in the air of milking parlours, and in exhaled cow breath, suggesting potential cattle-to-human transmission pathways [45], [46]. Infection may occur through splashes of contaminated milk, saliva or blood into the mucosa of the eyes, self-inoculation via contaminated hands to the eyes, nose, or mouth, or inhalation of infectious droplets while in close range to cattle. Importantly, pasteurization of H5N1-infected milk has been shown to inactivate the virus, making it safe for human consumption [181]. The WHO also advises against consuming raw milk and recommends consuming only pasteurized dairy products [182]. The ingestion pathway should be interpreted with caution, as further research is needed to examine this possible transmission pathway and to further assess risk using appropriate measures of association.

One study reported H5N1 infection after poultry exposure involving an open wound, in addition to direct hand contact with infected poultry [87], suggesting a possible percutaneous route of viral entry. However, in the reported study’s context, infection more likely occurred through direct contact (inoculation or self-inoculation) after contact with poultry or their secretions, as reported by the authors [87]. The report of a contaminated knife-related wound and bleeding being managed with contaminated hands likely reflects detailed epidemiological documentation/investigation rather than percutaneous transmission; therefore, this transmission pathway should be interpreted cautiously as it is weakly supported. Influenza A viruses are primarily respiratory and known to transmit through respiratory or mucosal routes [19]; however, infection through damaged skin remains hypothetically possible, particularly when direct contact occurs with infected blood, secretions, or contaminated materials [183] (given that the virus has been detected in poultry blood [[162], [184]]). Notably, live H5N1 has been isolated from human blood in Thailand [185] and high viral RNA levels were found in the blood of fatal human cases in Vietnam [186]. These findings highlight the need for further research on this potential (although likely rare) transmission pathway and to assess whether enhanced biosafety precautions (i.e., level 3 laboratory handling of blood samples) are warranted [185].

Both environmental and ocular pathways of H5N1 transmission are important to consider when assessing the potential zoonotic risk to humans. The virus has been detected in poultry feces, blood, and possibly in the mucus and saliva of both poultry and cattle [[162], [184]], fluids considered potentially infectious and classified as high-risk exposure sources by the CDC [172]. Reports of conjunctivitis among poultry and dairy workers infected with H5N1 (including cases where it was the only symptom) support the possibility of conjunctival mucosa inoculation as a transmission route [[90], [145], [146], [187]]. Environmental contamination has also been found in Cambodia, where H5N1 was found in mud, pond water, water plants, and soil samples [30]. Additionally, poor ventilation within poultry facilities may further increase the risk of transmission by increasing the concentration of airborne viral particles and aerosols [[188], [189]]. These aerosols, along with droplets and contaminated fomites, may be possible indirect routes of infection for humans, since infectious particles can remain on surfaces and suspended in the air for extended periods [[188], [190], [191]]. In terms of contaminated water as a potential source of environmental transmission, the CDC classifies contact with contaminated water or feed on farms with confirmed H5N1 cases in animals as a high-risk exposure behaviour, while possible contact with these sources is considered a medium-to-high risk exposure [172]. Infected animals have been found to contaminate water sources (e.g., ponds, lakes) with avian influenza viruses via fecal and tracheal secretions [[192], [193]], which are common sources of infection or reinfection for avian populations [[194], [195]]. Contaminated recreational areas may pose a potential risk for H5N1 transmission to humans, specifically through contact with infected birds; therefore, some public health organizations have deemed that temporary closure or avoidance of public areas (e.g., beaches, ponds, playgrounds) may be necessary in areas contaminated by dead birds or feces [[196], [197]]. Low salinity has been identified as a factor associated with longer persistence of avian influenza viruses in water [198], and HPAI H5N1 viruses have been shown to remain most stable in colder water with freshwater or brackish salinities [199]. Additionally, chlorination has been shown to inactivate H5N1 using a free chlorine residual between 0.52 and 1.08 mg/L and a one-minute exposure time [200]. This reinforces the need for further research in public health, hygiene, sanitation, and biosecurity practices. This includes further research in effectiveness of PPE use [e.g., goggles, respirators, and outer garments when close to potential infection sources [172]], disinfection protocols, as well as environmental surveillance [201].

The interaction between human and animal health, as well as sociocultural, environmental, and behavioural factors, all influence the risk of H5N1 transmission to humans. This highlights the need to address H5N1 within a One Health framework [202] that not only considers biological and environmental factors, but also equity and the social determinants of health. The majority of literature identified in this review originated from Asia, highlighting the endemic nature of H5N1 in poultry and the significant implications this poses for human health [203]. Continuous circulation of H5N1 among domestic and backyard poultry provides ongoing opportunities for viral exposure to humans and zoonotic transmission. The close and often unprotected contact between humans and poultry could make implementing effective infection control and surveillance strategies difficult. Structural barriers such as limited access to education, PPE, and healthcare infrastructure could further increase vulnerability to exposure and infection in low-resource settings. These inequities have been observed in other influenza A viruses (e.g., H1N1), which has disproportionately affected low-income countries partly due to limited access to healthcare and supporting health infrastructure [204]. Included literature from the United States and United Kingdom report human H5N1 cases from commercial poultry, dairy, and veterinary facilities, and less commonly from backyard poultry or ducks, which highlights geographic differences in exposure and context. These sources report the need for proactive, targeted public health strategies/protocols to reduce transmission risk in commercial dairy and poultry facilities, such as PPE provision and training, screening, testing, and pre-exposure prophylaxis with oseltamivir [90], [144], [147]. However, real-world compliance with PPE among workers in close contact with infected animals has been observed to be low, which highlights current monitoring and enforcement challenges [92]. Importantly, public health responses should prioritize health equity and cultural considerations, as noted specifically in the context of an American commercial poultry facility workforce mainly composed of Spanish-speaking migrant workers [90]. Targeted testing of both symptomatic and asymptomatic people, as well as active surveillance of high-risk exposure groups, may be a proactive, future research priority to support early detection, intervention, and prevent further transmission [110].

All studies in this review that reported suspected human-to-human transmission of H5N1 reported limited, inefficient, and non-sustained transmission. This is consistent with current understanding of the virus’s transmissibility in humans and aligns with established influenza transmission routes in humans and animals (droplet, aerosol, and contact) [19]. Most of this transmission reported in the literature occurred through caregiving activities, both in healthcare and household settings, where close, prolonged, and unprotected contact with infected cases was common. Transmission may occur through droplets contacting the eyes, nose, or mouth during close contact, self-inoculation of these mucosae after touching an infected person or their secretions, or inhalation of infectious droplets or aerosols at close range (e.g., during caregiving or respiratory procedures). Indirect transmission may occur via contaminated surfaces or items (e.g., bedding, clothing, high-touch surfaces), leading to self-inoculation after contact, or through inhalation of aerosols in shared or enclosed spaces. Activities such as bathing, cleaning, or handling contaminated materials may also generate droplets or aerosols and further contribute to transmission through mucosal contact or inhalation. Importantly, most clusters of cases (notably family clusters) are likely infected from a common poultry source [205]. Genetic susceptibility to H5N1 infection has been suggested as a possibility, which requires closer examination [[163], [206], [207]]. These findings highlight the importance of further research (including surveillance studies) in clinical, occupational, and community settings to detect cases.

The studies in this review that assessed antibody prevalence (seropositivity or seroprevalence) within exposed human populations showed evidence of subclinical, mild, or asymptomatic H5N1 infections. Although these cases are often less severe, they remain epidemiologically important. Mild or asymptomatic infections are opportunities for viral adaptation and mutation and can potentially lead to more highly pathogenic strains [[208], [209]]. To add, mild cases can transmit the virus to vulnerable populations, such as pregnant women, as well as children and young adults, who are more likely to experience severe or fatal outcomes [[210], [211], [212]]. The wide age range of cases included in this review emphasizes that H5N1 infection can occur across all age groups. The relatively young average age of cases (pooled mean of 23.9 years, median of 21.5 years) may reflect that younger people often work in household farms/backyard poultry settings or occupations with high exposure to animals (e.g., farming, agriculture) [213]; however, this could also be because this age group is more likely to have symptoms of severe infection that are reported [[210], [211]]. The relatively balanced distribution of males (44.1%) and females (38.7%) among studies that reported sex or gender, could reflect that both sexes and genders engage in high-risk activities [213]. Previous research found that males 26-40 years, followed by those aged 16-25 years, had the highest involvement in activities associated with H5N1 transmission risk [213]. The same study reported that both men and women participated in food preparation but in different roles: men more often slaughtered poultry and removed organs, while women typically boiled, cut, and washed the meat [213]. Considering gender in the context of One Health can help improve understanding of transmission risk to inform future research and public health strategies [214].

### 4.1 Limitations

Finally, this review has several limitations. Many reported transmission pathways and viral entry routes (into the human body) were based on self-reported exposures and the current understanding of how avian influenza viruses transmit to humans. Additionally, 50 sources that did not specify an exposure/transmission pathway were excluded, which may have included cases with unknown sources that could inform understanding of transmission dynamics. Given the high pathogenic nature of the virus, some cases may have died before contact/exposure history could be collected, which may have resulted in missing countries with confirmed human H5N1 cases. Because duplicate cases could not always be determined, some cases may have been reported in this review more than once. The included sources did not distinguish between sex and gender, and in some cases, did not specify which was being reported. Important factors that may influence observed seropositivity include prior infection/exposure [110] or vaccination (which may provide partial immunity to H5N1), as well as waning antibody levels over time [215]. Because this scoping review included both molecular and serological evidence of infection, transmission pathways should be interpreted with awareness of differing diagnostic certainty. Serological evidence was reported in 40 of 120 sources (33.3%) and indicates prior exposure of undefined timing, whereas molecular evidence confirms acute infection and supports a more precise exposure-outcome relationship. Consequently, pathways supported predominantly by seroprevalence or serological data, particularly some lower-frequency environmental and indirect routes, carry greater uncertainty as to whether the specific reported exposure was the actual source of infection. We did not restrict the synthesis to molecularly confirmed cases, in order to retain the full breadth of reported pathways consistent with the mapping objective of a scoping review. However, this means pathway frequencies should not be read as measures of transmission risk or causal attribution. Further research (including detailed epidemiological studies and systematic reviews) is needed to help determine the causal mechanisms of H5N1 transmission to humans, assess the quality of the evidence, and identify specific risk factors and their respective measures of association for infection, to strengthen and guide evidence-based prevention strategies.

## 5 Conclusions

This review provides a comprehensive mapping of all reported modes of H5N1 transmission to humans, including associated pathways and viral entry routes (based on biological plausibility and without implying relative likelihood or causal attribution), suggesting that there could be multiple pathways of exposure, and a single pathway may involve more than one entry route (and therefore multiple ways) to infect humans.

## CRediT authorship contribution statement

**Nicole Billias:** Writing – original draft, Visualization, Validation, Software, Project administration, Methodology, Investigation, Formal analysis, Data curation, Conceptualization. **Victoria D’Alessandro:** Validation, Software, Investigation, Data curation. **Dimitra V. Pouliopoulou:** Methodology, Investigation. **Jessica J. Wong:** Methodology. **Erin Miller:** Methodology. **Jessica P. Hopkins:** Writing – review & editing. **Eleni C. Boutsikari:** Writing – review & editing. **Lauren E. Cipriano:** Writing – review & editing. **Tiago da Veiga Pereira:** Writing – review & editing. **Jennie Johnstone:** Writing – review & editing. **Saverio Stranges:** Writing – review & editing. **J. Scott Weese:** Writing – review & editing. **Joy C. MacDermid:** Writing – review & editing, Supervision. **Kieran L. Quinn:** Writing – review & editing. **David N. Fisman:** Writing – review & editing. **Pavlos Bobos:** Writing – original draft, Supervision, Project administration, Methodology, Funding acquisition, Conceptualization.

## Funding

Nicole Billias is supported by a 10.13039/501100000024Canadian Institutes of Health Research (CIHR) Graduate Scholarship as well as a Canadian Behavioural Interventions and Trials Network (CBITN) Scholarship. Jessica Wong is supported by a Canadian Institutes of Health Research (CIHR) Research Excellence, Diversity, and Independence (REDI) Early Career Transition Award. This study was funded by the Canadian Institutes of Health Research (CIHR)’s Centre for Research on Pandemic Preparedness and Health Emergencies (CRPPHE) as part of a Catalyzing One Health Research on Avian Influenza funding opportunity (FRN: 196787). The funder had no role in study design, data collection and analysis, decision to publish, or preparation of the manuscript.

## Declaration of competing interest

Pavlos Bobos reports financial support was provided by the Canadian Institutes of Health Research. Joy C. MacDermid reports financial support from the Canadian Institutes of Health Research for her research program. David N. Fisman reports having served on advisory boards related to influenza and SARS-CoV-2 vaccines for Seqirus and Pfizer.

## Data Availability

Data will be made available on request.
