# Supplementary material for: Mapping Reported Modes of Transmission of Highly Pathogenic Avian Influenza A (H5N1) to Humans: A Scoping Review
Source: One Health. 2026 Jun 26;23:101492. doi: 10.1016/j.onehlt.2026.101492 (PMC13393788; doi:10.1016/j.onehlt.2026.101492)
Supplement: Supplementary file 1 — Supplementary material [file mmc1.docx]

**Supplemental File**

Mapping Reported Modes of Transmission of Highly Pathogenic Avian Influenza A (H5N1) to Humans: A Scoping Review. Nicole Billias, Victoria D’Alessandro, Dimitra V. Pouliopoulou, Jessica J. Wong, Erin Miller, Jessica P. Hopkins, Eleni Boutsikari, Lauren Cipriano, Tiago da Veiga Pereira, Jennie Johnstone, Saverio Stranges, J. Scott Weese, Joy C. MacDermid, Kieran L. Quinn, David Fisman, Pavlos Bobos

Table of Contents

[1. Search Strategy 2](#_Toc223613443)

[2. Data Extraction Form 6](#_Toc223613444)

# **Search Strategy**

**MEDLINE**

**Database: Ovid MEDLINE(R) ALL <1946 to October 29, 2025>**

**Search Strategy:**

1 Influenza in Birds/ (9510)

2 Influenza A Virus, H5N1 Subtype/ (7262)

3 (H5N1 or avian influenza* or avian flu or bird flu or influenza* A or influenza* A virus subtype H5N1 or fowl plague or H5Nx or H5N*).tw. (49070)

4 1 or 2 or 3 (50376)

5 Food Contamination/ or Equipment Contamination/ (63619)

6 Occupational Exposure/ or Environmental Exposure/ (150303)

7 (human or person or people or farmer or healthcare worker or agriculture worker or clinical worker or laboratory worker or family or contact or community or travel associated).tw. (5754597)

8 (bird or avian species or poultry or fowl or chicken or animal or mammal or seal or cow or bovine or cat or feline or dog or canine or ferret or pig or wild birds or migratory birds or ducks or geese or turkeys or swine or cattle or livestock or domestic animals).tw. (1597751)

9 (farm or pasture or agricultural land or farmland or ranch or homestead or cropland or dairy farm or livestock farm or poultry farm or barnyard or fields or agribusinesses or livestock farms or dairy farms or poultry farms or agro-industrial site or beef cattle farms or swine farm or mixed farms or agrotourism farms or urban farms or chicken coop or hen house or backyard or residence or house).tw. (498883)

10 Prevalence/ (380254)

11 Incidence/ (327195)

12 Risk Factors/ (1055440)

13 exp risk/ (1501788)

14 exp Cohort Studies/ (2808673)

15 Cross-Sectional Studies/ (563349)

16 Case-Control Studies/ (353706)

17 prevalen*.ti,ab,kf. (1177166)

18 incidence*.ti,ab,kf. (1074513)

19 ((risk* or associated*) adj2 factor*).ti,ab,kf. (1141388)

20 cohort*.ti,ab,kf. (1105797)

21 (cross-section* or crosssection* or (cross* adj2 section*)).ti,ab,kf. (713968)

22 (longitudinal* or prospectiv* or retrospectiv* or follow-up* or (follow* adj1 up*)).ti,ab,kf. (3477681)

23 (case-control* or (case adj1 control*)).ti,ab,kf. (184040)

24 occurence*.ti,ab,kf. (2375)

25 (rate* or frequenc*).ti,ab,kf. (5022283)

26 proportion*.ti,ab,kf. (876181)

27 observational study.pt. (185056)

28 Seroprevalence/ (27325)

29 seroepidemiolog*.ti,ab,kf. (5476)

30 Case Reports.pt. (2512033)

31 (case series or case report*).ti,ab,kf. (705864)

32 (Contamination or exposure or contact or handling or travel or ingestion or shedding or airborne or inhalation or droplet or aerosol or respiratory or secretions or consumption or interaction or fomite or surface or dust or water or waste or feces or environment or direct contact or close contact or transmission or contagion or spread or communicability or human-to-human or animal-to-human or animal-to-animal or fomite-to-human or fomite-to-animal).tw. (6938967)

33 (Risk ratio or odds ratio or logistic models or risk assessment or risk factor).tw. (757308)

34 Behavioral Risk Factor Surveillance System/ (2914)

35 5 or 6 or 32 or 34 (7010784)

36 7 or 8 or 9 (7309424)

37 10 or 11 or 12 or 13 or 14 or 15 or 16 or 17 or 18 or 19 or 20 or 21 or 22 or 23 or 24 or 25 or 26 or 27 or 28 or 29 or 30 or 31 or 33 (13274637)

38 4 and 35 and 36 and 37 (5467)

**Grey Literature Search**

The reference list of the following report from the World Health Organization (WHO) was manually searched: *Updated joint FAO/WHO/WOAH public health assessment of recent influenza A(H5) virus events in animals and people.* World Health Organization; 2025. <https://www.who.int/publications/m/item/updated-joint-fao-who-woah-public-health-assessment-of-recent-influenza-a(h5)-virus-events-in-animals-and-people-july2025>

Individual reported summaries of case investigations via the WHO’s Disease Outbreak News (DONs) from 2004 to 2025 were manually searched.

Website: <https://www.who.int/emergencies/disease-outbreak-news>

Internal search keywords: H5N1, Avian Influenza A(H5N1)

Date range: 1990 (oldest) to 2025 (newest at date of search)

Regions: All

Countries/Areas: All

A general web search was conducted to identify government and news sources reporting transmission or epidemiological information for countries with confirmed human H5N1 cases reported to the WHO. Key phrases and terms of the general web search (the first 10 sources were manually searched):

H5N1 human case Australia

H5N1 human case Azerbaijan

H5N1 human case Bangladesh

H5N1 human case Cambodia

H5N1 human case Canada

H5N1 human case Chile

H5N1 human case China

H5N1 human case Djibouti

H5N1 human case Ecuador

H5N1 human case Egypt

H5N1 human case India

H5N1 human case Indonesia

H5N1 human case Iraq

H5N1 human case Lao People's Democratic Republic

H5N1 human case Mexico

H5N1 human case Myanmar

H5N1 human case Nepal

H5N1 human case Nigeria

H5N1 human case Pakistan

H5N1 human case Spain

H5N1 human case Thailand

H5N1 human case Turkey

H5N1 human case United Kingdom of Great Britain and Northern Ireland

H5N1 human case United States of America

H5N1 human case Vietnam

Case investigation news articles were searched to find details on cases of H5N1 transmission to humans from the following sources:

- Center for Infectious Disease Research & Policy Research and Innovation Office (University of Minnesota)
  - Website: <https://www.cidrap.umn.edu/>
  - Internal search keywords: H5N1 case, H5N1
- U.S. Centers for Disease Control and Prevention (CDC) Newsroom
  - Website: <https://www.cdc.gov/media/index.html>
  - Internal search keywords: H5N1, H5N1 humans, H5N1 human case
  - Date range: All dates
  - Topic: Avian Flu (selected)
  - Audience: All
- Wisconsin Department of Health Services
  - Website: <https://www.dhs.wisconsin.gov/>
  - Internal search keywords: H5N1
- Iowa Department of Health and Human Services
  - Website: <https://hhs.iowa.gov/>
  - Internal search keywords: H5N1
- Central Nevada Health District
  - Website: <https://www.centralnevadahd.org/community-alerts/>
  - Internal search keywords: H5N1
- Ohio Department of Health
  - Website: <https://odh.ohio.gov/media-center/ODH-News-Releases>
  - Internal search keywords: H5N1, bird flu
  - Date range: All dates
- Pan American Health Organization
  - Website: <https://www.paho.org/en/timeline-influenza-ah5n1-americas-region>

# **Data Extraction Form**

| **Study Identification** | | | | | **Study Characteristics** | | **Population** | | | **Exposure** | **Context** | **Outcomes** | | | |
| --- | --- | --- | --- | --- | --- | --- | --- | --- | --- | --- | --- | --- | --- | --- | --- |
| **Author** | **Year** | **Title** | **Journal/Source** | **Country/Region** | **Study Design** | **Sample Size** | **Human Subgroups** | **Age** | **Males, Females (sex)** | **Type** | **High-Risk Settings** | **Suspected Primary Entry Route** | **Testing/Methods of Transmission Confirmation** | **Serological Evidence** | **Risk and Protective Factors Described** |
| Adisasmito | 2010 | Epidemiology of human avian influenza in Indonesia, 2005-2009: a descriptive analysis | Medical Journal of Indonesia | Indonesia | Case investigation/series | 93 (all H5N1) | Backyard Poultry Owners/Poultry Consumers, Close Contacts of H5N1 Cases, Live Bird Market Visitors | Median age 18 years (range 1 to 67 years) | 42 males, 51 females | Direct exposure to AI poultry (no human contact, i.e., slaughtering, handling excrement, consuming, having poultry deaths at home): 28 cases (30%)  Indirect exposure to AI poultry (no human contact): 14 cases (15%)  In vicinity of live poultry (no human contact, i.e., visiting a live poultry market): 34 cases (37%)  Human contact only: 2 cases (2%) Human and poultry exposure: 3 cases (3%) Exposure not known: 7 cases (8%) Deny exposure: 2 cases (2%) Bought and cooked poultry: 1 case (1%) Any wild bird: 2 cases (2%) | Backyard/Neighborhood Poultry, Live Bird Markets, Poultry Preparation Environments | **Mucosa (oral/nasal/conjunctival) Inhalation (respiratory)** | Laboratory confirmation consisted of one of the following: isolation of influenza A H5N1 virus; influenza A H5N1 detected by PCR; increase neutralizing antibody titer of H5N1 and convalescent specimen compared to acute specimen, and convalescent titer neutralizing antibody titer of 1/80 or greater; or neutralizing antibody titer of H5N1 of 1/80 or more on serum specimen on day 14 or later after onset. | Laboratory confirmation consisted of one of the following: isolation of influenza A H5N1 virus; influenza A H5N1 detected by PCR; increase neutralizing antibody titer of H5N1 and convalescent specimen compared to acute specimen, and convalescent titer neutralizing antibody titer of 1/80 or greater; or neutralizing antibody titer of H5N1 of 1/80 or more on serum specimen on day 14 or later after onset. | Not stated |
| Apisarnthanarak | 2004 | Atypical avian influenza (H5N1) | Emerging Infectious Diseases | Thailand | Case report | 1 (confirmed H5N1 case) | Backyard Poultry Owners/Poultry Consumers | 39 | 1 female | The patient and five family members live together in a rural area of Ayudhaya in central Thailand. **Her family reported that she was exposed to several dead chickens in her neighborhood. Neighborhood chickens were noted frequently to roam around the patient’s house, and some had died in front of it.** | Backyard/Neighborhood Poultry | **Mucosa (oral/nasal/conjunctival) Inhalation (respiratory)** | Nasopharnygeal aspirates were positive for influenza A H5 strain by two RT-PCR primers (Figure 2) and by realtime RT-PCR.   Viral culture for avian influenza H5N1 was conducted. Nasopharyngeal aspiration specimens tested positive by an RT-PCR assay specific for the hemagglutinin gene of influenza A H5N1. The specimen was tested with the primer set for the H5 gene. | Not stated | Not stated |
| Apisarnthanarak | 2010 | Detection by microneutralization of antibodies against avian influenza virus in an endemic avian influenza region | Clinical Microbiology and Infection | Thailand | Prospective cohort study (serologic) | 242 (6 low evidence of H5N1) | Commercial Poultry Facility Workers | Of 6 with mild/subclinical evidence of H5N1, mean age is 38 | 3 males, 3 females | Handled sick poultry (2 cases) Handled dying poultry (1 case) Culled healthy poultry in outbreak area (2 cases) Handled healthy poultry on farm (1 case) | Commercial Poultry/Swine/Beef Facilities | **Mucosa (oral/nasal/conjunctival) Inhalation (respiratory)** | Microneutralization of low-titre antibodies (anti-H5 micro-NT titres) and confirmatory Western blot assays. | Presence of low-titre anti-H5 antibodies | Poultry workers who had low anti-H5 micro-NT titres had longer daily exposure to poultry than those with negative titres (8 h vs. 2 h; p 0.02). The four participants with higher (>=1 : 20) anti-H5 micro-NT titres had longer daily exposure to poultry than the two with lower (<=1 : 10) titres (median, 8 h/day vs. 3 h/day, respectively; p 0.04). |
| Areechokchai | 2006 | Investigation of avian influenza (H5N1) outbreak in humans--Thailand, 2004 | Morbidity and Mortality Weekly Report (MMWR) Supplements - Centers for Disease Control and Prevention (CDC) | Thailand | Case-control | 16 (all H5N1), plus matched controls without H5N1 | Backyard Poultry Owners/Poultry Consumers, Residents of Poultry-Contaminated Environments, Close Contacts of H5N1 Cases, Live Bird Market Visitors | Median age 13 years (range 2 to 58 years) | 9 males, 7 females | Direct touching of unexpectedly dead poultry (death of greater than 10% of all poultry in a farm or house within 1 day or of greater than 40% of poultry within 3 days) (10 cases, 63%). OR: **29.0** (95% CI: **2.7-308.2**)  Dressing poultry (5 cases, 31%). OR: **17.0** (95% CI: **1.6-177.0**) Having unexpectedly dead poultry around the house (8 cases, 50%). OR: **5.6** (95% CI: **1.5-20.7**)  Plucking poultry (4 cases, 25%). OR: **14.0** (95% CI: **1.3-152.5**) Being 1 metre or less away from dead poultry (10 cases, 63%). OR: **13.0** (95% CI: **1.8-96.3**) Being 1 metre or less away from sick poultry (9 cases, 56%). OR: **3.8** (95% CI: **1.2-11.7**) Storing products of sick or dead poultry in house (7 cases, 44%). OR: **9.3** (95% CI: **2.1-41.3**) Direct touching of sick poultry (8 cases, 50%). OR: **5.6** (95% CI: **1.5-20.7**) Having contact with person with suspected or confirmed H5N1 illness (3 cases, 19%) OR: **0.9** (95% CI: **0.2-4.4**) Visiting live poultry market (1 case, 6%) | Backyard/Neighborhood Poultry, Poultry Preparation Environments, Poultry-Contaminated Environments, Live Bird Markets | **Mucosa (oral/nasal/conjunctival) Inhalation (respiratory)** | Nasopharyngeal aspirates or nasopharyngeal swabs  Cases were defined as H5N1 illness occurring in a person who had received a diagnosis of pneumonia or influenza-like illness and who had either a positive viral culture for H5N1 virus or confirmation of H5 strain by real-time reverse transcription-polymerase chain reaction (RT-PCR). | Not stated | Not stated |
| Bridges (Buxton) | 2000 | Risk of Influenza A (H5N1) Infection among Health Care Workers Exposed to Patients with Influenza A (H5N1), Hong Kong | Journal of Infectious Diseases | China | Historical cohort | 217 (exposed to H5N1) and 309 (not exposed to H5N1) **10 H5N1-positive** | Healthcare Workers | Of 10 health care workers who were H5N1 antibody positive, mean age is 36.3 | 3 males, 7 females | Exposed to H5N1 case-patient (8 cases) Provided direct patient care (5 cases) Physical contact (5 cases) Talked face-to-face (3 cases) Worked within 2 meters (7 cases) Recalled patient coughing/sneezing (1 case) Suctioned respiratory secretions/ administered breathing treatments (1 case) Changed bed linens (3 cases) Bathed patient (3 cases) Exposed to poultry (6 cases)  **Exposure of HCWs to H5N1 case-patients was associated with having H5-specific antibody. The seroconversion of 2 exposed HCWs strongly suggested that H5N1 was transmitted from patients to HCWs**. | Healthcare/Bedside Care | **Mucosa (oral/nasal/conjunctival) Inhalation (respiratory)** | Microneutralization and and Western blot assays | H5N1 antibodies (titers) greater than 80. Eight health care workers who were H5N1 antibody positive had paired acute and convalescent postexposure blood samples available for testing. Two anti-body positive health care workers with paired serum samples seroconverted. | Standard infection-control procedures had been followed; however, because the determination of H5N1 infection was not made until the day after death, additional droplet precautions,such as wearing masks, gloves, and gowns when within 3 feet of the patient, were not initiated |
| Bridges | 2002 | Risk of influenza A (H5N1) infection among poultry workers, Hong Kong, 1997-1998 | Journal of Infectious Diseases | China | Case-control | 293 government workers involved in poultry culling operation, 1525 poultry workers (1312 poultry workers included in nested case-control analysis). Of those that were positive via MN and WB assays:  **81 poultry workers H5N1 positive  9 government workers seropositive** | Commercial Poultry Facility Workers | Poultry workers: 15–29 years old: 3 30–44 years old: 56 45–59 years old: 22  Government workers: median age was 43 years (range, 37–55 years) | Positive poultry workers: 45 males, 36 females  Positive government workers: 8 males, 1 female | Work in retail poultry operation vs. wholesale/hatchery/farm/other poultry industry. OR: **2.7** (95% CI: **1.5–4.9**)  Reporting >10% mortality among poultry that they worked with. OR: **2.2** (95% CI: **1.3–3.7**)  Touching poultry. OR: **5.8** (95% CI: **0.9–113.6**) Butchering poultry. OR: **3.1** (95% CI: **1.6–5.9**) Feeding poultry. OR: **2.4** (95% CI: **1.4–4.1**) Collecting eggs. OR: **1.2** (95% CI: **0.6–2.2**) Cleaning poultry stalls. OR: **1.6** (95% CI: **0.9–2.7**) Touching poultry intestines. OR: **1.7** (95% CI: **0.9–2.9**) Handling money. OR: **1.6** (95% CI: **1.0–2.5**) Preparing poultry for restaurants. OR: **1.7** (95% CI: **1.1–2.7**)  **More-intensive poultry exposure, such as butchering and exposure to ill poultry, was associated with having anti–H5 antibody.** | Commercial Poultry/Swine/Beef Facilities | **Mucosa (oral/nasal/conjunctival) Inhalation (respiratory)** | Microneutralization and and Western blot assays | Detection of H5-specific antibodies. Serum samples were considered to be positive by microneutralization assay if anti-H5 titers of >80 were obtained. Among government workers, 3% were seropositive, and 1 seroconversion was documented. Among poultry workers, ~10% had anti–H5 antibody. | Government workers also often wore protective clothing, such as gowns, masks, and gloves, when working directly with poultry during the culling operation. Poultry workers had more-prolonged exposures to poultry. **The risk of having H5 antibody increased as the number of reported types of exposures increased.** |
| Buchy | 2007 | Influenza A/H5N1 virus infection in humans in Cambodia | Journal of Clinical Virology | Cambodia | Case investigation/series | 6 (all H5N1 confirmed cases) | Backyard Poultry Owners/Poultry Consumers, Close Contacts of H5N1 Cases | The mean age of the patients was 16 years (range 3–28) | 2 males, 4 females | **Except for patient 1 who nursed her sick brother**, all patients had a **history of contact with sick or dead poultry**. It is thought that she may have been infected when **nursing and wiping out the body of her bother** who had died with undiagnosed severe pulmonary infection 2 days prior to her symptoms onset and who was cremated before specimens could have been collected. | Backyard/Neighborhood Poultry, Healthcare/Bedside Care | **Mucosa (oral/nasal/conjunctival) Inhalation (respiratory)** | RT-PCR | Not stated | Not stated |
| Cao | 2013 | Avian influenza A (H5N1) virus antibodies in pigs and residents of swine farms, southern China | Journal of Clinical Virology | China | Cross-sectional study (serologic) | 16 (all H5N1) | Dairy or Swine Workers | Of 16 seropositive: median age 29 (range 19-52 years) | 11 males, 4 females | Most participants admitted having close contact with avian species in swine farms, and many participants indicated they sometimes **bathed or swam** in swine farm ponds, putting them in close contact with waterfowl and ducks.  Most of these participants reported **direct** **exposure to avian species around lakes adjacent to their homes.** | Commercial Poultry/Swine/Beef Facilities | **Mucosa (oral/nasal/conjunctival) Inhalation (respiratory) Gastrointestinal** | Hemagglutination inhibition (HI) and (neutralization) NT assays | All samples were tested by hemagglutination inhibition (HI) and HI-positive sera were tested for neutralizing antibodies by using a traditional neutralization method and supplementary HI titer ≥ 80 and NT titer ≥ 80 were considered as having positive evidence of previous H5N1 infection. | Not stated |
| Brooks | 2009 | Avian influenza virus A (H5N1), detected through routine surveillance, in child, Bangladesh | Emerging Infectious Diseases | Bangladesh | Case report | 1 (confirmed H5N1 case) | Backyard Poultry Owners/Poultry Consumers | 16 months | 1 male | In late January 2008, the father had brought home a well-appearing live chicken from a local market located 50 m from the house. The chicken was kept on a veranda outside the child’s room. The mother slaughtered the chicken inside the bathroom while the child slept; she did not report having washed her hands before she handled the child. The waste materials from the slaughter were then stored in a tied polyethylene bag near the house entrance for 2 hours before disposal.  One potential exposure was the healthy-appearing chicken that was brought inside the home. The child did not have direct contact with the chicken, although indirect contact was suggested because his mother handled him after slaughtering the chicken. The owner of the poultry shop where the chicken was purchased reported that 5%–10% of chickens had died each day during January 2008. | Poultry Preparation Environments | **Mucosa (oral/nasal/conjunctival) Inhalation (respiratory)** | Microneutralization assay, RT-PCR | Microneutralization assay, using the child’s subtype H5N1 isolate at CDC, demonstrated a 4-fold rise in subtype H5N1 neutralizing antibodies between the child’s January 2008 serum specimens (titer <20/20) and May 2008 (titer 160/80) serum specimens | Lack of hand hygiene (i.e., handwashing). |
| Cavailler | 2010 | Seroprevalence of anti-H5 antibody in rural Cambodia, 2007 | Journal of Clinical Virology | Cambodia | Cross-sectional study (serologic) | 700 villagers (18 seroprevalent for H5N1 antibodies) | Backyard Poultry Owners/Poultry Consumers, Residents of Poultry-Contaminated Environments, Close Contacts of H5N1 Cases | Of 18 seropositive: median age was 14.5 years (range: 3-77 years old) | 8 males, 10 females | Swam/bathed in pond. OR: **2.52** (95% CI: **0.98–6.51**) Cleaned poultry stalls. OR: **0.82** (95% CI: **0.29–2.33**) Collected poultry feces for manure. OR: **0.85** (95% CI: **0.11–6.56**) Touched/fed live poultry. OR: **0.63** (95% CI: **0.24–1.64**) Attended cockfight. OR: **1.47** (95% CI: **0.33–5.54**) Touched sick poultry. OR: **1.44** (95% CI: **0.55–3.77**) Plucked sick poultry. OR: **1.68** (95% CI: **0.59–4.80**) Eviscerated sick poultry. OR: **1.17** (95% CI: **0.38–3.61**) Washed sick poultry carcasses. OR: **1.33** (95% CI: **0.47–3.79**) Chopped/butchered sick poultry. OR: **1.60** (95% CI: **0.56–4.57**) Cooked sick poultry. OR: **1.17** (95% CI: **0.38–3.61**) Ate sick poultry. OR: **1.26** (95% CI: **0.48–3.29**) Any contact with sick poultry. OR: **1.12** (95% CI: **0.43–2.93**) Close contact with H5N1 case. OR: **1.48** (95% CI: **0.00–7.00**) Relative of the H5N1 case. OR: **0.91** (95% CI: **0.32–2.58**) | Backyard/Neighborhood Poultry, Poultry-Contaminated Environments, Hunting/Cockfighting Environments | **Mucosa (oral/nasal/conjunctival) Inhalation (respiratory) Gastrointestinal** | Microneutralization assay | According WHO recommendations, a serum was considered as positive when the neutralization antibody titer was ≥1:80 and when the HI test antibody titer was ≥1:160. | Not stated |
| Ceyhan | 2010 | Serosurveillance study on transmission of H5N1 virus during a 2006 avian influenza epidemic | Epidemiology and Infection | Turkey | Cross-sectional study (serologic) | 381 subjects (4 tested positive for H5N1) | Backyard Poultry Owners/Poultry Consumers, | Not stated | Not stated | All cases had a history of close contact with diseased or dead chickens   One mother of one of the cases and had close and continuous household contact with a diseased chicken. One of her children and the chicken had confirmed H5N1 infection and the chicken had been living in the house with family members. Both the child and the H5-antibody positive parent had eaten the infected chicken. Therefore, it is difficult to demonstrate that this was a case of human-to-human transmission. | Backyard/Neighborhood Poultry | **Mucosa (oral/nasal/conjunctival) Inhalation (respiratory) Gastrointestinal** | Microneutralization assay, ELISA, HI, positive cases confirmed via RT-PCR | 3 confirmed-positive (by PCR) cases were seropositive (confirmed by microneutralization and HI). One positive from ELISA.  Exposed family members: 1 positive microneutralization, 1 positive ELISA, 1 HI, 1 ELISA and HI positive  Culling staff: 5 seropositive by ELISA, 4 by HI.  Asymptomatic individuals living in the area with known contact with diseased chickens: 1 seropositive by HI  A symptomatic individuals living in the area with known contact without any known contact with diseased chickens: 81 positive by ELISA, 4 with HI, 1 positive by both ELISA and HI | The culling was performed with protection such as masks, glasses, caps, protective dress and gloves. However, all culling personnel reported at least one exposure to diseased chickens while not wearing PPE. |
| Chakraborty | 2017 | Mild Respiratory Illness Among Young Children Caused by Highly Pathogenic Avian Influenza A (H5N1) Virus Infection in Dhaka, Bangladesh, 2011 | Journal of Infectious Diseases | Bangladesh | Case investigation/series | 2 (both positive for H5N1) | Backyard Poultry Owners/Poultry Consumers, Live Bird Market Visitors | Both patients <5 years old (aged 13 months and female, and aged 31 months, male) | 1 male, 1 female | Chicken slaughtered at home (Patient A): Vendor slaughtered, defeathered, and skinned the chickens inside patient A’s home while patient A was present. Her father handled the chickens, washed his hands afterward only with water, and then held his daughter in his lap.  Touched chicken, chicken rubbed in wound (Patient B): Ten days before his illness, patient B had touched live poultry while his mother purchased a chicken from a roaming vendor. Blood from the chicken was smeared on his hand when the chicken was eviscerated. The next morning, he cut his hand with the knife his mother was using to prepare the raw chicken meat, and his mother pressed her hand on his wound to stop the bleeding without washing her hands. | Live Bird Markets, Poultry Preparation Environments | **Mucosa (oral/nasal/conjunctival) Inhalation (respiratory) Percutaneous (skin/abrasion)** | Tested positive via RT-PCR. Microneutralization and HI assays | Patient A met the WHO serology criteria for a confirmed H5N1 case.  Patient B did not meet the WHO criteria for seroconversion but was considered seropositive based on a microneutralization antibody titer ≥40. | Lack of hand hygiene (i.e., handwashing). |
| Chea | 2014 | Two clustered cases of confirmed influenza A(H5N1) virus infection, Cambodia, 2011 | Eurosurveillance | Cambodia | Case investigation/series | 2 (both positive for H5N1) | Backyard Poultry Owners/Poultry Consumers, Residents of Poultry-Contaminated Environments | Case 1: 9 months Case 2: 19 years | 1 male, 1 female | The likely cause of the fatal infection in the mother and the child was common-source exposure in Preah Sdach District, Prey Veng Province.  From 7 January onwards, **poultry in the family and two neighbouring households began to die. By 20 February, all chickens in the three households including the relatives’ 20 chickens had died.** Case 2 was reportedly present with Case 1 at all times. **Case 1 sat, played and crawled on the ground at their relatives’ and neigbours’ homes.** However, none of the cases visited the pond on the homestead or had direct contact with any sick, dead or slaughtered poultry. **Both cases reportedly spent time in areas surrounding the three households where chickens had died, including areas contaminated with feathers, feces and discarded waste of sick or dead poultry.**  As revealed by interviews, Case 2 had much less exposure to sick or dead poultry compared with other household members, and Case 1 had no direct exposure to sick or dead poultry. Yet they were infected while the other household members were not. **This may be due to higher levels of infective virus in the environment than that in the sick or dead poultry, which is unlikely.** Alternatively, genetic vulnerability could have been a crucial risk factor. | Backyard/Neighborhood Poultry, Poultry-Contaminated Environments | **Mucosa (oral/nasal/conjunctival) Inhalation (respiratory)** | qRT-PCR | Not stated | Not stated |
| Chokephaibulkit | 2005 | A child with avian influenza A (H5N1) infection | The Pediatric Infectious Disease Journal | Thailand | Case report | 1 (confirmed H5N1 case) | Backyard Poultry Owners/Poultry Consumers | 6 years old | 1 male | Additional history recording revealed that the **patient’s family and neighbors raised chickens in the back yard and let them feed freely in the yard and surrounding areas.** Almost every house in the village kept 5-10 egg-laying chickens and some chickens for cock fighting. **The patient had grown up with chickens and liked to play with them.** Approximately 500 meters away, his relatives had a chicken farm. It was an open farm with 300 broiler chickens. During the winter months of each year, chicken deaths were not unusual and were typically attributed to cold or infections. In 2003, chickens in the village started to die in late December. It seemed that more chickens died than in previous years. The symptoms of the sick chickens included diarrhea and hypersecretion, followed quickly by death. **All of the chickens in the relative’s farm were sick and died or were culled. Four of the 5 chickens in the patient’s house also died during that time. The patient had helped cull the sick chickens and carried one of them from the farm back home** on December 28. Typically sick chickens are culled, cooked and shared with neighbors; however, the patient’s family did not eat any sick chickens on this occasion. | Backyard/Neighborhood Poultry | **Mucosa (oral/nasal/conjunctival) Inhalation (respiratory)** | RT-PCR | Not stated | Not stated |
| Chotpitayasunondh | 2005 | Human disease from influenza A (H5N1), Thailand, 2004 | Emerging Infectious Diseases | Thailand | Case investigation/series | 12 H5N1-confirmed cases, 21 H5N1-suspected cases | Backyard Poultry Owners/Poultry Consumers, Residents of Poultry-Contaminated Environments | Confirmed cases: median age 12 years (range: 2-58)  Suspected cases: median age 33 years (range: 1-67 years) | Confirmed cases: 8 males (67%), and 4 females (33%)  Suspected cases: 15 males (71%), and 6 females (29%) | **Poultry contact: 58% of confirmed cases, 52% of suspected cases.** Of **confirmed** cases:  **Case 1:** Raised chickens in backyard. Chickens died unexpectedly 5 days before illness onset. Frequently played with chickens and had direct contact with carcasses. **Case 2:** Raised chickens in backyard, but chickens did not die. Two months before onset, ducks in a nearby area died unexpectedly. **Case 3:** Raised chickens in backyard. Three days before onset, chickens started to die. The last patient died on the date he became sick. He buried all carcasses. **Case 4:** Raised 60 chickens in back yard. All chickens died unexpectedly 1 month before onset. She burned and buried carcasses without protection. **Case 5:** Raised fighting cocks that died 4 days before onset. Reported direct contact with carcasses. Ate chicken with suspected H5N1 influenza. **Case 6:** No poultry in family. Helped slaughter one ill chicken 2 days before onset. **Case 7:** Mother slaughtered 2 ill chickens in house 4 days before onset. No direct contact with chickens. Mother got sick on same day and died without laboratory confirmation. **Case 8:** Chickens in backyard died unexpectedly. Grandfather slaughtered ill chickens. No direct contact with chickens but played near slaughtering area. **Case 9:** No poultry in family. Frequently played on ground near a chicken farm that reported unexpected poultry deaths. **Case 10:** Helped raise chickens in backyard. Eight days before onset, chickens died unexpectedly and patient assisted with slaughtering. **Case 11:** Factory worker living in province A during weekdays but in province B on weekends. Fighting cocks lived at a neighboring house. Province B reported outbreaks 2 months before onset. No contact with live or dead chickens. **Case 12:** Raised 40–50 chickens in backyard. Chickens started to die 5 days before onset. Buried and slaughtered ill chickens every day until onset date. | Backyard/Neighborhood Poultry, Poultry Preparation Environments, Poultry-Contaminated Environments, Hunting/Cockfighting Environments | **Mucosa (oral/nasal/conjunctival) Inhalation (respiratory) Gastrointestinal** | RT-PCR, immunofluorescence assay | Specimens were considered positive for avian influenza virus if the viral culture was positive and was confirmed by IFA with H5-specific monoclonal antibody provided by the WHO, if epithelial cells in clinical specimens were IFA positive for H5, or if the RT-PCR was positive with H5 specific primers (RT-PCR or real-time RT-PCR) | Mention of confirmed case 4 burying carcasses without protection. |
| de Jong | 2005 | Fatal avian influenza A (H5N1) in a child presenting with diarrhea followed by coma | New England Journal of Medicine | Vietnam | Case investigation/series | 2 (both positive for H5N1) | Residents of Poultry-Contaminated Environments | Case 1: 9 years old Case 2: 4 years old | 1 male, 1 female | **Water from a nearby canal was used for washing and, after boiling, for drinking**. Patient 1 **swam** regularly in this canal, as did other children in the neighborhood. At the time of the children’s illnesses, the family owned apparently healthy fighting cocks. Many chickens and ducks were present in the hamlet and canal during early 2004, but none were ill. All were culled in February as part of routine measures to contain the outbreak of influenza H5N1 in poultry. The parents did not handle poultry from markets  The routes of transmission in our patients are unclear. Epidemiologic investigations did not reveal exposure to ill poultry. In view of recent data suggesting that ducks infected with the current H5N1 strain shed large amounts of virus, **the source of transmission may have been domestic ducks present in the canal near the children’s house. Water from this canal was used for washing, and Patient 1 was reported to have swum regularly in this canal.** | Poultry-Contaminated Environments, Hunting/Cockfighting Environments | **Mucosa (oral/nasal/conjunctival) Gastrointestinal** | RT-PCR | Not stated | Not stated |
| Drehoff | 2024 | Cluster of Influenza A(H5) Cases Associated with Poultry Exposure at Two Facilities - Colorado, July 2024 | Morbidity and Mortality Weekly Report (MMWR) Supplements - Centers for Disease Control and Prevention (CDC) | United States (Colorado) | Case investigation/series | 663 workers screened for illness (109 reported symptoms and consented to testing, **9 with positive H5 test**) | Commercial Poultry Facility Workers | Of 9 workers who had positive test result, the median age was 32 years (range = 18–56 years) | 4 male, 5 female | Poultry depopulation activities (culling). Containerized gassing with carbon dioxide was determined as the preferred depopulation method in consultation with the Colorado Department of Agriculture and U.S. Department of Agriculture in accordance with American Veterinary Medical Association recommendations. Facility A used this method in 100% of bird houses, and facility B used it in approximately 55% of bird houses  Poultry depopulation activities and their attendant environments are associated with high potential for viral exposure at affected facilities. **In addition to handling and disposing of dead birds, the predominant depopulation method used at both facilities also involved handling each live bird, which increased exposure and the risk for displacement of or damage to PPE**, especially in these cage-free facilities where birds roam free and must be physically caught. | Commercial Poultry/Swine/Beef Facilities | **Mucosa (oral/nasal/conjunctival) Inhalation (respiratory)** | PCR. Nasopharyngeal swabs and conjunctival swab specimens were collected from workers reporting symptoms; swab specimens were tested for influenza A and A(H5) virus at the CDPHE laboratory. Specimens testing presumptively positive for influenza A(H5) virus or with inconclusive results were sent to CDC for confirmatory testing. Genetic sequencing performed as well. | Not stated | Among workers who were symptomatic (65 at Facility A and 44 at Facility B) Self-reported PPE use* Eye protection 28 (43) 38 (86) Mask 32 (49) 44 (100) Coveralls 23 (35) 41 (93) Gloves 20 (31) 43 (98) Boots or boot covers 12 (18) 41 (93) Head cover 20 (31) 34 (77) |
| Dinh | 2006 | Risk factors for human infection with avian influenza A H5N1, Vietnam, 2004 | Emerging Infectious Diseases | Vietnam | Case-control | 28 case patients (positive for H5N1) and 106 controls | Backyard Poultry Owners/Poultry Consumers, Residents of Poultry-Contaminated Environments, Live Bird Market Visitors | The mean age of the patients was 14 years (range 1–31) | 14 males, 14 females | Prepared and cooked healthy poultry. OR: **2.2** (95% CI: **0.6–10.4**) Prepared and cooked sick or dead poultry. OR: **31.0** (95% CI: **3.4–1150**) Helped prepare or cook sick or dead poultry. OR: **2.6** (95% CI: **0.8–8.7**) Bought live poultry for household consumption. OR: **1.2** (95% CI: **0.2–7.0**) Live poultry in household. OR: **3.0** (95% CI: **0.9–10.0**) Sick or dead poultry in household. OR: **7.4** (95% CI: **2.7–59.0**) Live poultry in neighborhood. OR: **1.07** (95% CI: **0.2–6.6**) Sick or dead poultry in neighborhood. OR: **3.9** (95% CI: **1.0–55.7**) Farm or family with >150 poultry within 100 m . OR: **1.0** (95% CI: **0.2–4.2**)  Household members work with commercial poultry. OR: **2.0** (95% CI: **0.0–38.4**) Exposed to patients with acute respiratory infection (temperature ≥38°C). OR: **2.4** (95% CI: **0.7–13.5**) Exposed to hospitalized patients with acute respiratory infection. OR: **2.4** (95% CI: **0.6–12.9**) | Backyard/Neighborhood Poultry, Live Bird Markets, Poultry-Contaminated Environments, Poultry Preparation Environments | **Mucosa (oral/nasal/conjunctival) Inhalation (respiratory)** | Persons who met any of the following criteria were considered to be laboratory-confirmed influenza A H5 case-patients: 1) influenza A H5–specific RNA detected in a single specimen by RT-PCR by using 2 different primer pairs; 2) influenza A H5 detected in a single specimen by RT-PCR identification and by sequencing or virus isolation; 3) influenza A H5–specific RNA detected by RT-PCR in 2 different specimen types (e.g., throat swab and tracheal aspirate); and 4) influenza A H5–specific RNA detected by RT-PCR in 2 samples taken on different days. | Not stated | Handwashing before eating (usually or sometimes). OR: 1.3 (95% CI: 0.3–5.6) Handwashing >3 times/d. OR: 0.53 (95% CI: 0.1–2.4) Wading in ponds, rice fields, ditches. OR: 2.4 (95% CI: 0.4–19.2) No indoor water source in household. OR: 5.0 (95% CI: 1.3–77.0) Poor hygiene conditions. OR: 1.0 (95% CI: 0.3–3.4) |
| Dung | 2014 | Seroprevalence survey of avian influenza A(H5N1) among live poultry market workers in northern Viet Nam, 2011 | Western Pacific Surveillance and Response Journal | Vietnam | Cross-sectional study (serologic) | 37 participants seropositive | Live Bird Market Workers | 0–24 years: 1 25–34 years: 11 35–44 years: 9 45 and above years: 16 | 13 males, 24 females | By occupation, the proportion seropositive was **higher in slaughterers and sellers** compared with all others (veterinarians, drivers, feather collectors, cleaners and market managers) but this was not significant (8.2% compared with 2.6%, P = 0.06). 37 participants seropositive for (21.9%), overall seropositive rate of 6.1%; (95% CI: 4.6–8.3). | Live Bird Markets | **Mucosa (oral/nasal/conjunctival) Inhalation (respiratory)** | Horse haemagglutination inhibition assay and microneutralization assay, RT-PCR. A sample was considered seropositive for influenza A(H5N1) virus antibody if an HHI titre ≥80 and an MN titre ≥20 were obtained in duplicate HHI and MN tests with any influenza A(H5N1) clade. | 37 participants seropositive for (21.9%), overall seropositive rate of 6.1%; (95% CI: 4.6–8.3). | Not stated |
| Garg | 2025 | Highly Pathogenic Avian Influenza A(H5N1) Virus Infections in Humans | New England Journal of Medicine | United States | Case investigation/series | 45 (all confirmed H5N1) | Commercial Poultry Facility Workers, Dairy or Swine Workers | Median age: 34 years | 36 males (80%), 9 females (20%) | All the case patients who were exposed to infected poultry were involved in depopulation activities. Among dairy workers, 4 (16%) were exposed to cows and 21 (84%) to both cows and raw milk.  **Poultry depopulation event: 20 people (44%) Direct contact with cows: 4 (9%) Raw milk and direct contact with cows (“Raw milk” refers to raw-milk consumption, raw-milk exposure, or both): 21 (47%)** | Commercial Poultry/Swine/Beef Facilities, Dairy Farms | **Mucosa (oral/nasal/conjunctival) Inhalation (respiratory)** | RT-PCR confirmation and genetic sequencing | Not stated | Gloves (71%) were the most frequently reported type of PPE, followed by eye protection (60%) and face masks (47%). Reported use of both eye protection and respirators or face masks was less common (36%). All categories of reported PPE use were higher among poultry workers than among dairy workers |
| Gilsdorf | 2006 | Two clusters of human infection with influenza A/H5N1 virus in the Republic of Azerbaijan, February-March 2006 | European Communicable Disease Bulletin | Azerbaijan | Case investigation/series | Cluster 1: 7 Cluster 2: 2 (8 confirmed, 1 probable case) | Backyard Poultry Owners/Poultry Consumers | Cluster 1: 10 to 20 year range (mean: 16 years; median: 17 years)  Cluster 2: 24 years (male), 18 years (female) | Cluster 1: 2 males, 5 females  Cluster 2: 1 male, 1 female | Cluster 1: In February 2006 a massive die-off of swans had occurred in the area and that the family might have had contact with the swans. Following further repeated interviews, relatives of the cases revealed that, in February 2006, **the family had been involved in de-feathering dead wild swans**. **Close contact with and de-feathering of infected wild swans were the most plausible exposures to infuenza A/H5N1** virus in the Daikyand cluster.  Cluster 2: Information provided by community members, however, suggested that the siblings had **purchased a dead turkey that was thought to have been ill, and then de-feathered it, prepared it and ate it**. | Poultry Preparation Environments | **Mucosa (oral/nasal/conjunctival) Inhalation (respiratory) Gastrointestinal** | RT-PCR (8 confirmed, and 1 probable case) | Not stated | Consumption of/contact with wild bird meat. |
| Giriputro | 2008 | Clinical and epidemiological features of patients with confirmed avian influenza presenting to Sulianti Saroso Infectious Diseases Hospital, Indonesia, 2005-2007 | Annals of the Academy of Medicine | Indonesia | Case investigation/series | 27 laboratory confirmed cases, 296 suspect cases | Backyard Poultry Owners/Poultry Consumers, Residents of Poultry-Contaminated Environments, Live Bird Market Visitors | Mean age of the confirmed cases was 16.9 years | 13(48.1%) males, 14 (51.9%) females | **The largest number of cases (12 or 44.4%) had indirect contact with poultry – predominantly by visiting markets or areas where outbreaks of poultry disease caused by H5N1 avian influenza had been reported.  Direct contact history referred to a patient who had direct contact with sick or dead poultry. Indirect contact referred to a patient who had contact with contaminated environments including fertiliser or animal markets.**  **4 cases with direct contact with poultry.  Surprisingly, we found more patients with an indirect contact history than with a direct contact history. This is possibly because of transmission through fertilisers made from chicken manure.**  Unfortunately, for a significant number of cases (11 cases), the contact history could not be definitively obtained partly because the disease was often rapidly fatal by the time the patients presented to the hospital. | Backyard/Neighborhood Poultry, Live Bird Markets, Poultry-Contaminated Environments | **Mucosa (oral/nasal/conjunctival) Inhalation (respiratory)** | RT-PCR, or a 4-fold rise in H5 antibody titres on 2 serum specimens collected at least 2 weeks apart | RT-PCR, or a 4-fold rise in H5 antibody titres on 2 serum specimens collected at least 2 weeks apart | Not stated |
| Gomaa | 2020 | Incidence and Seroprevalence of Avian Influenza in a Cohort of Backyard Poultry Growers, Egypt, August 2015-March 2019 | Emerging Infectious Diseases | Egypt | Prospective cohort study (serologic) | 5 (confirmed H5N1 cases) | Backyard Poultry Owners/Poultry Consumers, Live Bird Market Visitors | Median age: 10 (range: 5-27 years) | 2 males, 3 females | The first case was in a 5-year-old boy with **exposure to poultry at the household and at a live bird market.**  Case 2 was in an 11-year-old girl with **direct contact with chickens and ducks.**  Case 3 was in a 5-year-old boy with **direct contact with chickens and ducks.**  Case 4 was in a 27-year-old woman who had **direct contact with chickens and ducks.**   The H5N1 case was in a 10-year-old girl with **direct contact with chickens and ducks.** | Backyard/Neighborhood Poultry, Live Bird Markets | **Mucosa (oral/nasal/conjunctival) Inhalation (respiratory)** | RT-PCR, confirmed by sequencing | The absence of hemagglutination was considered a positive test result for antibodies to the virus. Virus microneutralization assay positivity was considered at an endpoint titer of >1:80. | Not stated |
| Gomaa | 2015 | Avian influenza A(H5N1) and A(H9N2) seroprevalence and risk factors for infection among egyptians: A prospective, controlled seroepidemiological study | Journal of Infectious Diseases | Egypt | Prospective cohort study (serologic) | 15 subjects positive for anti-A(H5N1) antibodies at baseline | Backyard Poultry Owners/Poultry Consumers | Median: 27 (range: 9 to 71 years old) | 5 males, 10 females | Exposure setting (exposed to poultry): Backyard: 731 (97.5%), live bird market: 3 (0.4%), commercial farm: 16 (2.1%) Days exposed per week (among those exposed to poultry): ≤2: 221 (29.8%), 3–6: 66 (8.9%), 7: 455 (61.3%)  **Baseline: Positive in exposed group: 15 (2.1%), Negative: 693 (97.9%) Follow-up 1: Positive in exposed group: 3 (0.4%), Negative 679 (99.6%) Follow-up 2: Positive in exposed group: 4 (0.6%), Negative 645 (99.4%)**  **For 15 subjects positive to H5N1 at baseline: All were backyard poultry growers. Their ages were 9 to 50 years, and 10 were female.** Only 1 of 15 subjects maintained a positive titer over the study duration; the others had negative titers at follow-ups 1 and 2  In bivariate analysis, chronic lung disease **(unadjusted OR: 11.0, 95% CI: 3.5–34.5, adjusted OR: 12.6, 95% CI: 3.8–41.7)**, exposure to geese **(unadjusted OR: 3.6, 95% CI: 1.2–10.2)**, and exposure to turkeys **(unadjusted OR: 3.8, 95% CI: 1.3–11.3)**  were associated with seropositivity against A(H5N1) virus. In multivariate analysis, only chronic lung disease remained significantly associated with A(H5N1) seropositivity | Backyard/Neighborhood Poultry | **Mucosa (oral/nasal/conjunctival) Inhalation (respiratory)** | Microneutralization assay and hemagglutination inhibition assay. A titer of ≥80 was considered positive. | A titer of ≥80 was considered positive. | None of the subjects reported consistent use and proper decontamination of personal protective equipment. |
| Hien (Tran) | 2004 | Avian influenza A (H5N1) in 10 patients in Vietnam | New England Journal of Medicine | Vietnam | Case investigation/series | 10 (all confirmed H5N1) | Backyard Poultry Owners/Poultry Consumers, Residents of Poultry-Contaminated Environments, Live Bird Market Visitors | Mean age: 13.7 years (range: 5 to 24 years) | 6 males, 4 females | **Patient 1:** Family members are farmers who do not keep poultry, but many chickens in neighborhood unexpectedly died in the preceding 2 wk; mother died of influenza A (H5N1) Jan. 9, 2004. **Patient 2:** No information available on exposure to sick poultry; 7-yr-old sister died of acute respiratory illness on Dec. 29, 2003. **Patient 3:** Family members are farmers who kept chickens, which died unexpectedly 5 days before onset of illness. **Patient 4:** Family members are farmers who kept chickens, which died 2 wk before onset of illness; chickens died in patient’s house and neighbors’ houses during week before onset of illness. **Patient 5:** Patient bought duckling as pet and cared for it in her house for 5 days; duck had diarrhea and died, patient buried it, dug it up a day later and reburied it; both patient and brother handled duck; patient also ate barely cooked eggs (Vietnamese delicacy) 2 days before onset of illness; neighbors kept 40 chickens, but no illness reported in these birds; fever developed in patient 3 days after she bought duck. **Patient 6:** Frequently attended cockfights, held roosters and chickens; no illness reported in the chickens or in 20 people involved in cockfighting; patient walked through live-poultry market 50 m from house on his way to school. **Patient 7:** Extensive exposure, including handling of 10 dead or dying chickens in patient’s homestead; father and patient prepared dead chickens for eating (removed feathers, washed, cut meat) 3 days before onset of illness. **Patient 8:** Direct handling of 50 chickens, including dead chickens, at home (which was also a restaurant); patient and father prepared chickens for eating. **Patient 9:** Direct handling of chickens in patient’s homestead 3 days before onset of illness; he prepared dead chickens for eating. **Patient 10:** Direct handling of sick ducks, chickens in patient’s home; many sick poultry in district. | Backyard/Neighborhood Poultry, Live Bird Markets, Poultry-Contaminated Environments, Poultry Preparation Environments, Hunting/Cockfighting Environments | **Mucosa (oral/nasal/conjunctival) Inhalation (respiratory) Gastrointestinal** | RT-PCR | Not stated | Not stated |
| Huo | 2012 | Seroprevalence of avian influenza A (H5N1) virus among poultry workers in Jiangsu Province, China: an observational study | BMC Infectious Diseases | China | Cross-sectional study (serologic) | 306 samples from poultry workers  **(8 seropositive for H5N1)** | Backyard Poultry Owners/Poultry Consumers | Median age: 64 years | 2 males, 6 females | **Job duties: feeding poultry, collecting eggs, cleaning poultry stalls.**  **Poultry number was identified as a novel risk factor associated with human infection with avian H5N1 virus. OR: 2.39 (95% CI: 1.00-5.69). Workers associated with raising larger poultry flocks have a higher risk of seropositivity.** | Backyard/Neighborhood Poultry | **Mucosa (oral/nasal/conjunctival) Inhalation (respiratory)** | Haemagglutination inhibition assay. | According to WHO recommendations, a serum sample was considered as positive when the HI test antibody titer was ≥1:160. | Compared with poultry workers from large scale poultry farms, backyard workers may have an even higher risk due to the lack of preventive measures and healthy environment. |
| Ilyicheva | 2018 | Humoral immunity to influenza in an at-risk population and severe influenza cases in Russia in 2016–2017 | Archives of Virology | Russia | Cross-sectional study (serologic) | 28 (all seropositive for H5N1) | Backyard Poultry Owners/Poultry Consumers, Commercial Poultry Facility Workers | Not stated | Not stated | There was no clinical evidence of human disease; however, analysis of sera from people who had **contact with infected or perished birds** **during the outbreaks of highly pathogenic influenza** in January–March 2017 in the European part of Russia, demonstrated the presence of antibodies to A/rook/Chany/32/2015 (H5N1). | Backyard/Neighborhood Poultry | **Mucosa (oral/nasal/conjunctival)** | Hemagglutination inhibition (HI) tests, microneutralization assays (for serological), PCR with cell culture confirmation, HI test results were confirmed by real-time PCR data, genetic sequencing | Hemagglutination inhibition (HI) tests, microneutralization assays (for serological), PCR with cell culture confirmation, HI test results were confirmed by real-time PCR data, genetic sequencing | Not stated |
| Kandeel | 2010 | Zoonotic transmission of avian influenza virus (H5N1), Egypt, 2006-2009 | Emerging Infectious Diseases | Egypt | Case investigation/series | 63 confirmed cases (all positive for H5N1) | Backyard Poultry Owners/Poultry Consumers, Live Bird Market Visitors | 0–4 years (23 cases) 5–14 (11 cases) 15–49 (27 cases) ≥50 (2 cases) | 23 males, 40 females | * Risk factor data on exposure to birds were available for 41 case-patients. Handling live domestic poultry likely infected with avian influenza virus (H5N1) was the primary source of exposure. Investigations showed that human-to-human transmission was unlikely; even clusters of case-patients had exposure to infected poultry. **Recently purchased domestic poultry from market/seller: 12 (29.2%) Recently purchased poultry became ill: 7 (58.3%) Noted illness or death among their birds: 33 (80.5%) Bred birds: 14 (51.8%) Slaughtered birds in past 10 days: 13 (48.1%) Defeathered birds in past 10 days: 13 (48.1%)** | Backyard/Neighborhood Poultry, Live Bird Markets | **Mucosa (oral/nasal/conjunctival) Inhalation (respiratory)** | PCR | Not stated | Not stated |
| Kandun | 2010 | Chicken faeces garden fertilizer: possible source of human avian influenza H5N1 infection | Zoonoses and Public Health | Indonesia | Case report | 1 (positive for H5N1) | Gardeners | 37 years old | 1 female | **The patient was known to be a keen gardener, and she used to purchase garden fertilizer (chicken faeces) in sealed bags from a gardening shop to use for her potted plants.**   A bag of chicken faeces garden fertilizer was found at the patient’s home. The husband stated that the bag was purchased before the patient’s onset, but the exact dates of purchase or manufacture were unknown. The label on the bag indicated that the fertilizer came from a company in East Java (over 1000 km away). Through trace-back, we found out that the company purchased chicken faeces from a variety of collectors for inclusion in their product, and that the collectors sourced the faeces from numerous farms, as far as hundreds of kilometres away.  **The chicken faeces (garden fertilizer) collected from the index patient’s home also tested H5N1 positive by RT-PCR.** From the epidemiological aspects of this investigation, we found that the patient had direct and unprotected contact with the H5N1-contaminated fertilizer. Our results also confirmed the prolonged (>2 weeks) environmental stability of H5N1 virus in bags of chicken faeces garden fertilizer. | Gardens | **Mucosa (oral/nasal/conjunctival) Inhalation (respiratory)** | RT-PCR, sequencing | Not stated | **She did not wear gloves or a mask whilst gardening.** |
| Kandun | 2006 | Three Indonesian clusters of H5N1 virus infection in 2005 | New England Journal of Medicine | Indonesia | Case investigation/series | 8 (7 confirmed, 1 probable H5N1 case) | Backyard Poultry Owners/Poultry Consumers, Residents of Poultry-Contaminated Environments, Close Contacts of H5N1 Cases, Gardeners | The median age of the patients was 8.5 years (range, 1 to 38) | 5 males, 3 females | Cluster 1: Patient 1A, an 8-year-old girl in whom fever, headache, nausea, vomiting, and rhinorrhea developed, was hospitalized with pneumonia 6 days after the onset of symptoms. Had mechanical ventilation for respiratory failure, but she died on the 26th day of illness. Patient 1B was a 1-year-old girl in whom fever developed 1 week after the onset of illness in her sister (Patient 1A). She was placed on mechanical ventilation but died on the 12th day of illness. **Patient 1C, who worked as a government auditor, was the 38-year-old father of Patients 1A and 1B. He had close contact with his sick daughters at home and during their hospitalizations.** He had onset of fever 3 and 9 days, respectively, after the onset of his daughters’ illnesses  Cluster 2: Patients 2A and 2B **had chickens die nearby**, and **poultry were slaughtered daily approximately 50m from the home**. In her home garden, Patient 2A **used fertilizer containing poultry feces** that **tested positive for H5N1** by RT-PCR.  Cluster 3: In mid-September 2005, **backyard chickens started dying in the village**. Three days after **holding two dead chickens**, symptoms started. H5N1 virus was isolated from a lung specimen obtained from a chicken close to the home. Patient 3B, the 5-year-old brother of Patient 3A, had symptom onset the same day as Patient 3A. Patient 3C was the 4-year-old son of a sister of Patient 3A and Patient 3B. **He lived in a separate home and did not have contact with his uncles during their illnesses but moved to their home after they were hospitalized**. Patient 3C did not have any known contact with ill persons or with sick or dead poultry, but **his mother had handled dead chickens and buried them** | Backyard/Neighborhood Poultry, Poultry-Contaminated Environments, Healthcare/Bedside Care, Gardens | **Mucosa (oral/nasal/conjunctival) Inhalation (respiratory)** | RT-PCR, viral culture, molecular sequencing, antiviral resistance testing, microneutralization, and Western blot analyses | Serologic confirmation of H5N1 virus infection was made more than 3 months later for Patient 3B | Not stated |
| Katz | 1999 | Antibody response in individuals infected with avian influenza A (H5N1) viruses and detection of anti-H5 antibody among household and social contacts | Journal of Infectious Diseases | China | Prospective cohort study (serologic) | 17 (confirmed cases of H5N1) | Backyard Poultry Owners/Poultry Consumers, Residents of Poultry-Contaminated Environments, Close Contacts of H5N1 Cases, Live Bird Market Visitors | Median age 13 years, age range: 59 years (1–60) | 7 males, 10 females | Case patient 4: All tour members **rode buses daily with case patient 4 (the longest ride was ∼3 h) and shared 1 or 2 airline flights** of 3.5 h duration. Twenty-two tour members **either shared a meal, talked with the H5N1-infected person, or were close by when the ill person coughed**. One (4%) of 26 individuals was positive for neutralizing antibody to H5N1 virus in the first serum sample, collected 12 days after initial exposure to the case patient. The H5-antibody–positive individual had been exposed to the case patient on the airplane, on the tour buses, by eating a meal and talking with him, and by being nearby, but **at a distance of 12 m**, when the case patient coughed.  Six (12%) of 51 household contacts were positive for antibody to H5N1 virus. These 2 household contacts both had **close physical contact** that included **hugging, kissing, or cuddling the infected individuals (i.e., caring for while ill)** to whom they were exposed.  7 cases exposured to poultry, defined as: having ever lived or worked on a poultry farm and whether, since November 1, 1997, they had shopped at a market or stall (an urban retail business) that sold live poultry, had live or freshly butchered poultry in the home, had poultry butchered at the home, or had contact with any poultry or pet birds that appeared sick, had yellow diarrhea, or had died. | Backyard/Neighborhood Poultry, Live Bird Markets, Poultry-Contaminated Environments, Poultry Preparation Environments, Healthcare/Bedside Care | **Mucosa (oral/nasal/conjunctival) Inhalation (respiratory)** | Microneutralization assay or an H5-specific ELISA, each followed by confirmation with a Western blot assay | Sera were considered to be positive in the microneutralization assay if anti-H5 titers of >80 were obtained in 2 independent microneutralization assays performed at 1 or both testing facilities. A confirmatory Western blot assay was performed at the CDC on all sera that were positive by the microneutralization assay. Sera from persons <14 years old were also tested by an indirect ELISA. Sera that were positive for IgG or IgM in both ELISA and Western blot were considered positive for anti-H5 antibody. 2 cases were seroconverted. | Not stated |
| Khuntirat | 2011 | Evidence for subclinical avian influenza virus infections among rural Thai villagers | Clinical Infectious Diseases | Thailand | Prospective cohort study (serologic) | 73 with elevated antibody titers for H5N1 (2 different strains) | Backyard Poultry Owners/Poultry Consumers, Residents of Poultry-Contaminated Environments | Not stated | Not stated | **Not having an indoor water source** was a significant risk factor for exposure to either A/Thailand/676/2005 or A/Thailand/384/2006 HPAI H5N1 virus even after controlling for confounding variables (**adjusted OR: 3.2, 95% CI: 1.7–6.1, adjusted OR: 3.1, 95% CI: 1.4–6.7**)  **Any poultry exposure** for A/Thailand/384/2006(H5N1): **Unadjusted OR: 3.3 (95% CI: 1.1–13.1)** Self-reported poultry exposure, defined as ever being within 1 meter of live poultry for 30 consecutive minutes, was prevalent among the participants (65.4%) | Backyard/Neighborhood Poultry, Poultry-Contaminated Environments | **Mucosa (oral/nasal/conjunctival) Inhalation (respiratory) Gastrointestinal** | Hemagglutination inhibition (HI) assays, microneutralization (MN) assays | A low threshold of antibody titer (≥ 1:10) as evidence of previous infection with an avian influenza strain (MN). A HI titer ≥ 1:40 was accepted as evidence of human or swine influenza virus infection or human influenza vaccination | Not stated |
| Kongchanagul | 2011 | Decreased expression of surfactant protein D mRNA in human lungs in fatal cases of H5N1 avian influenza | Journal of Medical Virology | Thailand | Case investigation/series | 1 (confirmed H5N1 case) | Not stated | 48 years old | 1 male | The diagnosis of avian influenza was suspected on day 4 of illness after a history of **direct contact with a dying chicken** was revealed. | Not stated | **Mucosa (oral/nasal/conjunctival)** | Virological diagnosis was done by **antigen detection, viral culture, and RT-PCR** on a nasopharyngeal wash specimen and was confirmed by a **seroconversion of neutralizing antibody** against H5N1 virus. The virus was identified to be avian influenza virus (H5N1) by **sequencing** | Seroconversion stated…cut-offs not stated. | Not stated |
| Kudo | 2012 | Clinical preparedness for severe pneumonia with highly pathogenic avian influenza A (H5N1): experiences with cases in Vietnam | Respiratory Investigation | Vietnam | Case investigation/series | 8 (all confirmed H5N1 infection) | Backyard Poultry Owners/Poultry Consumers, Residents of Poultry-Contaminated Environments, Live Bird Market Workers | Aged 2–30 years, median: 22.5 years (range: 2-30 years) | 2 males, 6 females | Two patients were chicken traders and another 2 reported cooking and eating infected (dead) poultry. One patient was a fish farmer who handled dried bird excrement as feed. The remaining 4 patients of young age (2, 8, 17, and 23 years old) had no history of direct contact with either sick or dead poultry; however, they lived in areas where H5N1 poultry outbreaks had been reported at the same time as their illness  **Patient 1: Handled chickens Patient 2: Ate infected chicken Patient 3: Backyard poultry died 5 days before onset Patient 4: cooked and ate infected chicken Patient 5: Epidemic outbreak in poultry in residential area Patient 6: Fish farmer, handled bird excrement Patient 7: Chicken trader, handled dead chickens Patient 8: Epidemic outbreak in poultry in residential area** | Backyard/Neighborhood Poultry, Live Bird Markets, Poultry-Contaminated Environments, Poultry Preparation Environments | **Mucosa (oral/nasal/conjunctival) Inhalation (respiratory) Gastrointestinal** | All patients tested positive for H5N1 virus by real-time reverse transcriptase-polymerase-chain-reaction (RT-PCR) | Not stated | Not stated |
| Kwon | 2012 | Avian influenza a (H5N1) virus antibodies in poultry cullers, South Korea, 2003-2004 | Emerging Infectious Diseases | South Korea | Prospective cohort study (serologic) | 9 (all H5N1) | Commercial Poultry Facility Workers | Median: 25 years (range: 22–48 years) | 9 males | Among the 2,512 persons, MN assay results were confirmed positive for 9. The US CDC confirmed positive results in a single sample for 4 persons; the Korea CDC confirmed positive results in paired samples for 5 others. Among the 9 persons with positive MN results, only 2 had positive results according to horse hemagglutination inhibition assay; however, all 9 had clear reactivity to H5 proteins on Western blot analysis and were confirmed positive according to WHO criteria (Table 2). All those with influenza (H5N1)–positive results were male, median age was 32.5 years (range 22–48 years), and **all had participated in culling during the outbreaks** (Table 2). | Commercial Poultry/Swine/Beef Facilities | **Mucosa (oral/nasal/conjunctival) Inhalation (respiratory)** | Microneutralization assay, hemagglutination inhibition assay, Western blot analysis | Microneutralization assay, hemagglutination inhibition assay, Western blot analysis. MN: results were considered to be positive if titers against H5 were ≥80 according to at least 2 independent assays. | Not stated |
| Lafond | 2019 | Seasonal Influenza and Avian Influenza A(H5N1) Virus Surveillance among Inpatients and Outpatients, East Jakarta, Indonesia, 2011-2014 | Emerging Infectious Diseases | Indonesia | Cross-sectional cohort | 1 (confirmed H5N1 case) | Live Bird Market Visitors | 33 years | 1 male | The H5N1 virus–positive case-patient was a 33-yearold man (Table 4) with an onset of illness of June 1, 2014. On June 4, he was admitted into a hospital, where he received a diagnosis of pneumonia (chest radiograph data unavailable) and was enrolled into surveillance; his respiratory specimen was collected on June 6. On June 13, he was transferred to a designated referral hospital, where he began antiviral treatment and died on June 14. He was obese but had no other concurrent medical condition; **exposure history included visiting a live bird market, where he purchased live poultry within 7 days before hospital admission.** This case was not linked to any documented bird outbreaks. No animal or environmental specimen collection occurred because of the length of time that elapsed before linkage to a live poultry market and because of rapid turnover of poultry through these markets | Live Bird Markets | **Mucosa (oral/nasal/conjunctival) Inhalation (respiratory)** | Nasal and throat swab samples: RT-PCR | Not stated | Not stated |
| Le | 2013 | Subclinical avian influenza A(H5N1) virus infection in human, Vietnam | Emerging Infectious Diseases | Vietnam | Case report | 2 (both positive for H5N1) | Backyard Poultry Owners/Poultry Consumers | 40 years, 18 years | 1 male, 1 female | The man’s **household had sick poultry that were consumed by household members**. The chickens roamed close to the sleeping area of the household members. The index case-patient, his daughter, and his daughter-in-law were involved in **slaughtering and preparing the chickens.**  The patient we investigated probably was exposed during **slaughtering of a chicken 6 days before her positive throat swab was collected. However, because chickens in the commune tested positive at the time of the contact investigation, ongoing exposure to influenza A(H5N1) cannot be excluded as the source of infection.** | Backyard/Neighborhood Poultry, Poultry Preparation Environments | **Mucosa (oral/nasal/conjunctival) Inhalation (respiratory) Gastrointestinal** | RT-PCR, sequencing, hemagglutination inhibition (HI) and microneutralization (MN) | Hemagglutination inhibition (HI) and microneutralization (MN).  Serologic testing showed seroconversion only in the woman with subclinical infection; her HI titer increased from <20 to 160 against both clade 2.3.4 and 2.3.2.1 viruses | All household members were given oseltamivir (75 mg/d) for 1 week |
| Leonard | 2025 | Notes from the Field: Seroprevalence of Highly Pathogenic Avian Influenza A(H5) Virus Infections Among Bovine Veterinary Practitioners - United States, September 2024 | Morbidity and Mortality Weekly Report (MMWR) Supplements - Centers for Disease Control and Prevention (CDC) | United States | Cross-sectional study (serologic) | 150 bovine veterinary practitioners (3 seropositive for H5N1) | Veterinary Professionals | Not stated | Not stated | **Three (2%; 95% CI = 0.7%–5.7%) survey participants had antibodies to HPAI A(H5) suggestive of recent HPAI A(H5) infection**; all were U.S.-based practitioners.   All three practitioners with positive serology results **provided care to multiple animals**, including dairy cattle; two also provided care to nondairy cattle, one provided care to poultry, and one worked at livestock markets. None worked with dairy cattle with known or suspected HPAI A(H5) virus infection; however, one practitioner did work with HPAI A(H5) virus–positive poultry. Two of the participants with a positive serologic test result reported practicing in multiple U.S. states, and two practiced in states with known HPAI A(H5) infection among cattle (Figure). However, one reported providing veterinary care to dairy cattle only in Georgia and to nondairy cattle in South Carolina; these states had not previously reported HPAI A(H5) infection in dairy cattle.  Practitioners with evidence of HPAI A(H5) virus infection also reported exposure to goats or sheep (three), swine (two), and cats (three). One practitioner reported providing veterinary care to another (unspecified) animal not listed in the survey (i.e., not dairy cattle; other cattle; swine, pigs, or hogs; alpacas or llamas; poultry; wild birds including wild waterfowl; goats or sheep; or cats).  **Practitioners reported the following activities when working with dairy cattle: intervening during calving or maternity care (two); surgery (two); mammary exam or treatment administration (one); feeding (two); drenching, tubing, or administering oral medications (one); administering vaccines or injectable medications including intravenous fluids (two); podiatry (one); pregnancy checking (three); and postmortem examinations or necropsies (two).** | Veterinary Care | **Mucosa (oral/nasal/conjunctival) Inhalation (respiratory)** | Microneutralization assay, hemagglutinin inhibition assay | A positive serologic test result is defined as antibody titers ≥40 by the microneutralization assay and titers ≥40 by the hemagglutinin inhibition assay against a wild type influenza A(H5N1) 2.3.4.4b A virus | All reported wearing gloves or a clothing cover when providing veterinary care to cattle (including a variety of clinical activities, such as pregnancy checking or surgery); none reported wearing respiratory or eye protection |
| Le | 2019 | Fatal avian influenza A(H5N1) infection in a 36-week pregnant woman survived by her newborn in Soc Trang Province, Vietnam, 2012 | Influenza and Other Respiratory Viruses | Vietnam | Case report | 1 (confirmed H5N1 case) | Backyard Poultry Owners/Poultry Consumers | 26 years old | 1 female (pregnant, 36 weeks) | The case‐patient's family raised a flock of 40 backyard chickens, which started dying off on January 8, 2012. **Prior to the onset of symptoms, the case‐patient and her mother‐in‐law slaughtered 6 sick chickens between January 17 and 22, 2012.**  Through interviewing the neighbors and the father of the case‐patient, the investigation team found that between January 8 and 18, 2012, backyard chickens became sick and died off in the hamlet, and these poultry had not received the avian influenza vaccine. Poultry exposure history of the case‐patient was confirmed | Backyard/Neighborhood Poultry | **Mucosa (oral/nasal/conjunctival) Inhalation (respiratory)** | (RT‐PCR), and viral isolation and genome sequencing | Not stated | Not stated |
| Liao | 2013 | Seroprevalence of Antibodies to Highly Pathogenic Avian Influenza A (H5N1) Virus among Close Contacts Exposed to H5N1 Cases, China, 2005-2008 | PLoS One | China | Cross-sectional study (serologic) | 2 seropositive for H5N1 antibodies | Backyard Poultry Owners/Poultry Consumers, Close Contacts of H5N1 Cases | 4 years old, 43 years old | 1 male, 1 female | Overall, only two of the 419 close contacts had serum that met our definition as testing seropositive for HPAI H5N1 virus antibodies. The first seropositive case was the 4-year old daughter of an H5N1 case that survived. The child **slept with her mother and had unprotected direct and close physical contact with her for 5 days from the mother’s illness onset on 11 February 2006 to her hospital admission date**. During this period, the mother experienced high fever and productive cough. **Poultry were raised in the backyard of the index case’s household.** Six days before the index case’s illness onset, **13 chickens were found to be sick and later died quickly.**  The second seropositive case was the 43-year old father of an H5N1 case**. The father had close contact with his ill son** for 9 days after the son’s illness onset until he died. The father **provided unprotected bedside care when his son was sick**. **Three days before the index patient’ illness onset, chickens in the neighbor’s household began to die and all were dead within three days.** **Five chickens in the index case’s household began to die on two days after the index case’ illness onset and all were dead within two days.** | Backyard/Neighborhood Poultry, Healthcare/Bedside Care | **Mucosa (oral/nasal/conjunctival) Inhalation (respiratory)** | Hemagglutinin inhibition (HI) assay. Sera with an HI titer of ≥40 were then tested by microneutralization (MN). | An individual was defined to be seropositive for HPAI H5N1 virus antibody for the purposes of this study modified from WHO criteria: (1) For single serum, an HPAI H5N1 virus neutralizing antibody titer of ≥40 for study subjects aged 14 years old or younger, or ≥80 for those aged 15–59 years old, with an HI titer of ≥40; or (2) a four-fold or greater rise in neutralizing antibody titer against HPAI H5N1 virus in paired acute and convalescent sera, with the convalescent serum having a neutralizing antibody titer of ≥80 for adults or ≥40 for children or an HI titer of ≥40. | First seropositive case: All close contacts did not wear any protective equipment during their exposure to the index case.   Second seropositive case: The father provided unprotected bedside care when his son was sick |
| Li | 2013 | Evidence for H5 avian influenza infection in Zhejiang province, China, 2010-2012: a cross-sectional study | Journal of Thoracic Disease | China | Cross-sectional study (serologic) | 55 seropositive for H5N1 antibodies | Backyard Poultry Owners/Poultry Consumers, Commercial Poultry Facility Workers, Live Bird Market Workers | 15-30 years: 6 31-46 years: 21 47-62 years: 23 63-78 years: 5 | 33 males, 22 females | A total of **55 participants were seropositive** for influenza virus (H5N1) HI antibodies. 33 males, 22 females. 13 from live bird markets, 25 from large scale poultry companies, 17 from household members who bred backyard poultry  **Direct or close contact with poultry: 52 (94.5%) Direct or close contact with sick or dead poultry: 10 (18.2%)** Direct or close contact with poultry was significantly associated (**OR: 5.203, 95% CI: 1.526-17.736**) with an increased risk of being H5N1-seropositive. And the number of poultry bred more than 1,000 was also found to be associated with a **3.774** fold increased risk (**95% CI: 1.721-8.726**) | Backyard/Neighborhood Poultry, Live Bird Markets, Commercial Poultry/Swine/Beef Facilities | **Mucosa (oral/nasal/conjunctival) Inhalation (respiratory)** | Hemagglutination inhibition (HI) assays and microneutralization assays | According to WHO recommendations, an individual was deemed to be seropositive for H5N1 antibody if HI antibody titers of 1:160 or greater was detected. All the positive samples and 5% of the negative samples randomly selected were confirmed by micro-neutralization (MN) assay in Chinese NIC | Not stated |
| Ly | 2016 | Environmental contamination and risk factors for transmission of highly pathogenic avian influenza A(H5N1) to humans, Cambodia, 2006-2010 | BMC Infectious Diseases | Cambodia | Historical cohort study (serologic) with nested case control | 35 seropositive cases (plus 115 matched control subjects were enrolled in the case-control studies) | Backyard Poultry Owners/Poultry Consumers, Residents of Poultry-Contaminated Environments | Of 35 positive cases: median age 16 years (range: 3-77 years) | Of 35 positive cases: 19 males (54.3%), 16 females (45.7%) | Serology testing detected 35 (1.5%) subjects with positive results suggesting recent exposure to H5N1.  Among subjects of all ages, the following exposures were more frequently reported by cases positive by H5N1 serology compared to matched controls:  **Having a poultry cage or nesting area located under or right next to the house (77.8 % vs 52.9 %; p = 0.060, OR: 3.2 95%CI: 0.9−11.0) Swimming in ponds also accessed by poultry (40 % vs 30.4 %; p = 0.099, OR: 2.1 95%CI: 0.9−4.8) Swimming in ponds also accessed by wild birds (47.1 % vs 29.2 %; p = 0.157, OR: 2.2 95%CI: 0.7−6.8) Handling sick/dead wild birds (20.0 % vs. 8.7 %; p = 0.131, OR: 2.4 95%CI: 0.8−7.8) Transporting poultry to market (12.0 % vs. 2.2 %; p = 0.050, OR: 6.0 95%CI: 1.0−35.9)** Among subgroup of 22 cases aged <20 and their 77 matched control subjects: **Eating wild birds as food (50.0 % vs. 26.9 %; p = 0.054, OR: 3.5 95%CI: 0.98−12.7)** Among the 13 cases aged ≥20 years and their 38 control subjects: **Gathering and placing domestic poultry in the cages or designated poultry area was somewhat more frequent among seropositive cases (69.2 % vs. 39.5 %; p = 0.104, OR: 3.0 95%CI: 0.8−11.6)** | Backyard/Neighborhood Poultry, Live Bird Markets, Poultry-Contaminated Environments | **Mucosa (oral/nasal/conjunctival) Inhalation (respiratory) Gastrointestinal** | Microneutralization assays, Western blot assays, hemagglutination inhibition assays | Sera from 2006 were tested by microneutralization (MN) assay and when the titer was ≥1:80, the result was confirmed by a Western blot assay, as recommended by WHO.  Sera from 2007–2009 were screened using H5 hemagglutinin pseudotyped lentiviral particles. Those with a titer ≥1:20 were further tested by MN and hemagglutination inhibition (HI) assay. Antibody titers ≥1:80 in duplicate MN assays and ≥1:160 in duplicate HI test were considered positive as per WHO recommendations. | Not stated |
| Ma | 2018 | Avian Influenza A Virus Infection among Workers at Live Poultry Markets, China, 2013-2016 | Emerging Infectious Diseases | China | Prospective cohort study (serologic) | 18 seropositive for H5N1 (7/18 seroconversions) | Live Bird Market Workers | Of 7 poultry workers that seroconverted: Median age: 48 years (range: 39-60 years) | Of 7 poultry workers that seroconverted: 1 male, 6 females | Of 964 enrolled poultry workers **18** were **seropositive** for H5N1 during the study period. **7** poultry workers **seroconverted** for H5N1.  Among poultry workers, **female sex** (adjusted OR: 5.48 [95% CI: 2.38–12.62]) and **exposure to pigeons** (adjusted OR 3.13 [95% CI 1.23–8.00]) were also significant risk factors for H5N1 virus seropositivity or seroconversion.  Characteristics of poultry workers with seroconversion of influenza A (H5N1): **1 chicken/duck/goose seller 1 chicken/duck/pigeon raising 1 pigeon seller 1 chicken/goose seller 1 duck/goose seller 2 chicken slaughterers** | Live Bird Markets | **Mucosa (oral/nasal/conjunctival) Inhalation (respiratory)** | Hemagglutination inhibition (HI) assays and microneutralization assays | We defined a seropositive result as an MN titer ≥80 for all tested viruses. Seroconversion was defined as detection of a ≥4-fold rise in MN antibody titer between initial serum sample and a paired second serum sample, with the second sample achieving a titer ≥80. | Not stated |
| Mellis | 2024 | Serologic Evidence of Recent Infection with Highly Pathogenic Avian Influenza A(H5) Virus Among Dairy Workers - Michigan and Colorado, June-August 2024 | Morbidity and Mortality Weekly Report (MMWR) Supplements - Centers for Disease Control and Prevention (CDC) | United States | Cross-sectional study (serologic) | 8 seropositive for H5N1 | Dairy or Swine Workers | Not stated | Not stated | Among the 115 dairy workers, eight (7%; 95% CI = 3.6%–13.1%) had serologic evidence of infection with A(H5) virus (both neutralizing antibody titers and HI antibody titers ≥1:40).  All persons with a positive serology result were Spanish speakers, **all reported cleaning the milking parlor**, and **most (88%) reported milking cows**. | Dairy Farms | **Mucosa (oral/nasal/conjunctival) Inhalation (respiratory)** | Microneutralization (MN) assays and hemagglutinin inhibition (HI) assays | Persons with a GMT ≥1:40 on both MN and HI assays were considered to have serologic evidence of HPAI A(H5) virus infection; all other results were considered negative. | None of the workers with serologic evidence of infection used respiratory protection; three used recommended eye protection. Among the eight workers with evidence of infection, only one reported close contact with cows known to be infected.  None of the workers with HPAI A(H5) virus antibodies reported using the PPE recommended for working with HPAI A(H5)–infected animals, and use of recommended PPE was low among all workers |
| Monamele | 2019 | Evidence of exposure and human seroconversion during an outbreak of avian influenza A(H5N1) among poultry in Cameroon | Emerging Microbes & Infections | Cameroon | Cross-sectional study (serologic) | 16 seropositive (of which 2 seroconverted) for H5N1 | Commercial Poultry Facility Workers, Live Bird Market Workers | Not stated | Not stated | Despite not being able to detect any active avian influenza infections in poultry workers, longitudinal serosurvey suggested both prior exposure and seroconversion against Cameroonian strains of HPAIV A(H5N1) among **poultry farm and LBM workers exposed to diseased or dead poultry**. Of the 131 participants selected for serological analysis, 16 (12.2%) had the possible presence of antibodies against A (H5N1) isolates from Cameroon with a reciprocal HAI titre ≥ 10 on the second sampling. Three (2.3%) of these individuals had reciprocal HAI titre ≥ 20. Of the individuals positive in the second sampling, 2 (1.5%) were found to have a 4-fold increase in HAI titre between the first and second serum collections, suggesting seroconversion. | Live Bird Markets, Commercial Poultry/Swine/Beef Facilities | **Mucosa (oral/nasal/conjunctival) Inhalation (respiratory)** | Hemagglutination inhibition (HI) assays and microneutralization assays | The second set of sera were first analysed by HAI for the likelihood of patients with seroconversion. Samples with detectable antibodies in the second serological draw (HAI titre ≥ 10) were then re-tested by HAI and MN assay utilizing both the first and second serum samples. Exposure to these viruses was considered “suspected” with an HAI titre ≥ 40 in the second serum sample and/or seroconversion defined as the detection of antibodies above the thresholds (fourfold increase) defined following no detection of antibodies in the serum sample from the previous period. | Not stated |
| Morse | 2024 | Influenza A(H5N1) Virus Infection in Two Dairy Farm Workers in Michigan | New England Journal of Medicine | United States (Michigan) | Case investigation/series | 2 (both positive for H5N1) | Dairy or Swine Workers | Not stated | Not stated | Dairy worker MI-A had discomfort in the right eye 1 day after **milk** **had splashed in that eye while the worker was milking a cow at a farm that had confirmed HPAI A(H5N1) in dairy cows**.   MI-B: This worker’s duties involved **caring for ill cows, including administering oral fluid therapy (“drenching,” which typically involves direct handling of oral secretions)**. | Dairy Farms | **Mucosa (oral/nasal/conjunctival) Inhalation (respiratory)** | RT-PCR | Not stated | MI-A: The worker had not been using personal protective equipment.  MI-B: The worker used eye protection and gloves but did not use a respirator or a mask. |
| Mounts | 1999 | Case-control study of risk factors for avian influenza A (H5N1) disease, Hong Kong, 1997 | Journal of Infectious Diseases | China | Case-control | 15 hospitalized cases with confirmed H5N1, plus ≥ 2 controls for each case | Close Contacts of H5N1 Cases, Live Bird Market Visitors | Of case patients: 1–10 years: 8 (53%)  11–20 years: 3 (20%) 20–60 years: 4 (27%)  Median age 6 years (range: 1.5–60 years) | 6 males (40%), 9 females (60%) | Exposed to live poultry in market (9/14, 64%). **OR: 4.5 (95% CI: 1.2–21.7)**  9 (64%) of 14 case patients reported poultry exposure (visiting either a poultry stall or a retail market selling live poultry) in the week before illness onset.  Althouth 1 case patient had contact with another child with influenza A (H5N1), raising the possibility of human-to-human transmission, both children also lived near a poultry stall from which H5N1 virus was cultured. | Live Bird Markets | **Mucosa (oral/nasal/conjunctival) Inhalation (respiratory)** | Viral culture, microneutralization assay, Western blot | The infection status of each case subject was established in earlier investigations either by viral culture or by serologic tests showing a 4-fold rise in H5-specific antibody. Positive sera were retested by a Western blot assay. A positive H5N1 antibody test required a microneutralization antibody titer ≥80 on 2 occasions and a positive Western blot | Not stated |
| Nasreen | 2015 | Highly pathogenic Avian Influenza A(H5N1) virus infection among workers at live bird markets, Bangladesh, 2009-2010 | Emerging Infectious Diseases | Bangladesh | Prospective cohort study (serologic) | 18 seropositive for H5N1 (6 seroconversions) | Live Bird Market Workers | Median age 27 years | 18 males, 0 females | Of 404 poultry workers, **6 (2%) seroconverted**. **12 seropositive workers. 17 (94%) of 18** who were seropositive or seroconverted against H5N1 reported exposure to poultry through >1 activity. Highest RR: **feeding poultry (17, 94%), cleaning feeding trays and water containers (15, 83% and 16, 89%), not washing hands after working with sick poultry (10, 56%), and cleaning feces from pens (14, 78%);** classified as **high exposure. Simple RR: 4.8 (95% CI: 0.8–28.2)**. **Multiple RR: 7.6 (95% CI: 2.8–20.9).**  Second highest RR: **slaughtering (17, 94%), defeathering (15, 83%%), eviscerating (15, 83%), collecting or transporting feces (1, 6%), and stuffing poultry into bags (14, 78%);** classified as **medium exposure**. **Simple RR: 3.5 (95% CI: 0.8–14.7). Multiple RR: 5.1 (95% CI: 1.8–14.1).**  Lowest RR: smoking (7, 39%), medicating poultry (2, 11%), isolating sick poultry (10, 56%), and eating raw or undercooked poultry or eggs (6, 33%); classified as low exposure. **Simple RR: 1.0 (95% CI: 0.3–3.3).** Poultry workers who frequently performed **high-exposure behaviors had a 7.6 times higher risk for H5N1 virus infection** compared with poultry workers who infrequently performed high-exposure behaviors when they also infrequently performed medium-exposure behaviors. **Poultry workers who frequently performed medium-exposure behaviors had a 5.1 times higher risk of H5N1 virus infection** compared with poultry workers who infrequently performed medium-exposure behaviors when they also infrequently performed high-exposure behaviors.  Risk of infection from: Medium-exposure behaviors when frequently performing both medium- and high-exposure behaviours: **Multiple RR: 1.4 (96% CI: 0.3–6.2)**. High-exposure behaviors when frequently performing both high- and medium-exposure behaviours: **Multiple RR: 2.1 (96% CI: 0.4–12.9)**. | Live Bird Markets | **Mucosa (oral/nasal/conjunctival) Inhalation (respiratory) Gastrointestinal** | Microneutralization assay with confirmatory Western blot | A seropositive result was defined as an H5N1 virus microneutralization titer ≥40 (equivalent to WHO criteria ≥80) and confirmation by an H5-specific Western blot.   Seroconversion against H5N1 virus was defined as detection of a ≥4- fold rise in microneutralization antibody titer between the initial serum sample and a paired second serum sample, with the second sample achieving a titer ≥40 | Of 6 workers that seroconverted, none used PPE, 3/6 washed hands at the market after working with the poultry, and 6/6 changed clothes upon returning home. |
| Nguyen (Hien) | 2009 | Human infection with highly pathogenic avian influenza virus (H5N1) in northern Vietnam, 2004-2005 | Emerging Infectious Diseases | Vietnam | Case investigation/series | 29 (all confirmed H5N1 patients) | Backyard Poultry Owners/Poultry Consumers, Close Contacts of H5N1 Case | Mean: 35.1 (SD: ± 14.4) | 15 males, 14 females | High-risk exposure, no. (%) **Poultry (a history of exposure to sick or healthy poultry):** **19 (65.5%)** **Sick poultry:** **12 (41.4%)** **Family infected with H5N1 virus subtype:** **6 (20.7%)** **Sick poultry or person (a history of exposure to sick poultry or a family infected with avian influenza, H5N1): 15 (51.7%)** | Not stated | **Mucosa (oral/nasal/conjunctival) Inhalation (respiratory)** | RT-PCR | Not stated | Not stated |
| Okoye | 2013 | Serologic evidence of avian influenza virus infections among Nigerian agricultural workers | Journal of Medical Virology | Nigeria | Prospective cohort study (serologic) | 1 (seropositive for H5N1) | Commercial Poultry Facility Workers | Not stated | 1 male | The unexposed subject seropositive for antibodies against the H5N2 influenza virus was also observed to have **elevated antibodies (1:80) against the A/Chicken/Nigeria/1132123/2007(H5N1) influenza virus. A secondary interview with this subject revealed approximately 2 years ago he had helped to process 12 live broiler chickens for eating.** | Commercial Poultry/Swine/Beef Facilities | **Mucosa (oral/nasal/conjunctival) Inhalation (respiratory)** | Hemagglutination inhibition (HI) assay, microneutralization (MN) assay | A low threshold of antibody titer (≥1:10) was chosen as evidence of previous infection with a strain of avian influenza virus. | Not stated |
| Oliver | 2022 | A case of avian influenza A(H5N1) in England, January 2022 | Eurosurveillance | England | Case report | 1 (confirmed H5N1 case) | Backyard Poultry Owners/Poultry Consumers, Residents of Poultry-Contaminated Environments | Early 80s | Not stated | Exposed to infected birds and their secretions (flock of 125 Muscovy ducks in a domestic setting) Had close contact with avian influenza H5N1-infected birds in a heavily contaminated environment.   Had a high degree of close contact with a large number of infected birds and a virus-contaminated enclosed domestic environment which resulted in infection | Backyard/Neighborhood Poultry, Poultry-Contaminated Environments | **Mucosa (oral/nasal/conjunctival) Inhalation (respiratory)** | RT-PCR | Not stated | Did not use personal protective equipment (PPE) while exposed to infected birds and their secretions |
| Oner | 2006 | Avian influenza A (H5N1) infection in eastern Turkey in 2006 | New England Journal of Medicine | Turkey | Case investigation/series | 8 (confirmed H5N1 cases) | Backyard Poultry Owners/Poultry Consumers | Median age 10 years (range: 5 to 15 years) | 3 males, 5 females | Because all of these patients **shared living space** with poultry during the cold winter conditions, they had **direct contact with the secretions and feathers of diseased or dead birds** | Backyard/Neighborhood Poultry | **Mucosa (oral/nasal/conjunctival) Inhalation (respiratory)** | RT-PCR assay, ELISA, rapid influenza test | Not stated | Likely no PPE: shared living space with infected poultry |
| Potdar | 2022 | Identification of Human Case of Avian Influenza A(H5N1) Infection, India | Emerging Infectious Diseases | India | Case report | 1 (confirmed H5N1 case) | Residents of Poultry-Contaminated Environments | 11 years | 1 male | An in-depth interview with family members indicated that **the patient often frequented a family-owned poultry business** and **may have been exposed to birds with undetected infection**, although no infected domestic or wild avian sources or any environmental contamination had been reported in or around the residence of the child. | Commercial Poultry/Swine/Beef Facilities | **Mucosa (oral/nasal/conjunctival) Inhalation (respiratory)** | RT-PCR, genetic sequencing, BLAST, isolation and strain detection using MDCK cells | Not stated | Not stated |
| Rahman | 2020 | Evaluation of potential risk of transmission of avian influenza A viruses at live bird markets in response to unusual crow die-offs in Bangladesh | Influenza and other Respiratory Viruses | Bangladesh | Cross-sectional cohort | 151 workers (2 with positive PCR samples for H5N1) | Live Bird Market Workers | Not stated | Not stated | **1 H5N1-positive PCR 1 sample positive for H5N1 and H9 subtypes**  Almost all stalls slaughtered and defeathered birds. All LBMs had visible poultry feces on the ground, but no dead birds. Six LBMs reported market cleaning more than once daily, but only one used disinfectant (eg, bleach). Three LBMs reported disposal of solid waste at least twice daily. Half of the markets had open drains. About 60% of stalls experienced poultry deaths in the week before the investigations, and some workers reported discarding poultry carcasses as garbage or giving them to other workers | Live Bird Markets | **Mucosa (oral/nasal/conjunctival) Inhalation (respiratory)** | RT-PCR | Not stated | All LBMs had visible poultry feces on the ground, but no dead birds. Six LBMs reported market cleaning more than once daily, but only one used disinfectant (eg, bleach). Three LBMs reported disposal of solid waste at least twice daily. Half of the markets had open drains. About 60% of stalls experienced poultry deaths in the week before the investigations, and some workers reported discarding poultry carcasses as garbage or giving them to other workers |
| Sedyaningsih | 2007 | Epidemiology of cases of H5N1 virus infection in Indonesia, July 2005-June 2006 | Journal of Infectious Diseases | Indonesia | Case investigation/series | 54 (confirmed H5N1 cases) | Backyard Poultry Owners/Poultry Consumers, Residents of Poultry-Contaminated Environments, Commercial Poultry Facility Workers, Live Bird Market Workers | Median age: 18.5 years (range: 18 months–45 years) | 33 males, 21 females | 41 case patients (76%) had **direct or indirect contact with poultry** **(healthy appearing, sick, or dead)** during the 2 weeks preceding illness onset. Direct poultry contact was defined as touching healthy-appearing, sick, or dead poultry. Indirect poultry contact was defined as the presence of poultry within 25 m of a case patient’s home.  Among the 41 case patients who had poultry exposures, 23 had direct contact, such as **slaughtering or handling of sick birds**; the 18 other case patients with indirect contact had healthy, sick, or dead poultry in their neighborhood, although handling of such birds could not be confirmed. Six case patients (11%) with direct poultry contact had poultry-related occupations.  **21 of 54 cases of H5N1 virus infection occurred in 7 clusters**. A cluster of cases was defined as at least 2 epidemiologically linked ill persons with laboratory evidence of H5N1 virus infection among close contacts. Close contacts were defined as persons who had been within 1 m of a case patient during illness. | Backyard/Neighborhood Poultry, Live Bird Markets, Commercial Poultry/Swine/Beef Facilities, Poultry-Contaminated Environments | **Mucosa (oral/nasal/conjunctival) Inhalation (respiratory)** | RT-PCR, hemagglutination inhibition assay, microneutralization assay | 4-fold increase in H5N1 antibody titer between paired acute and convalescent serum samples (positive result). | Not stated |
| Setiawaty | 2015 | Avian Influenza A(H5N1) Virus Outbreak Investigation: Application of the FAO-OIE-WHO Four-way Linking Framework in Indonesia | Zoonoses and Public Health | Indonesia | Case report | 1 (confirmed H5N1 case) | Live Bird Market Visitors | 2.5-years | 1 male | The parents reported that **the case had visited a LBM** with his mother on 8 June, 2 days before onset of symptoms. They went to the poultry area of the LBM to buy fresh chicken meat. The mother purchased chopped chicken meat; however, the vendor also had live poultry for consumers who wanted to select a live bird to be slaughtered, defeathered and dressed at the stall. | Live Bird Markets | **Mucosa (oral/nasal/conjunctival) Inhalation (respiratory)** | RT-PCR | Not stated | Not stated |
| Shinde | 2011 | A comparison of clinical and epidemiological characteristics of fatal human infections with H5N1 and human influenza viruses in Thailand, 2004-2006 | PLoS One | Thailand | Historical cohort | 25 (confirmed H5N1 cases) | Backyard Poultry Owners/Poultry Consumers, Residents of Poultry-Contaminated Environments, Commercial Poultry Facility Workers | Median age 18 years (range: 1.6–68 years) | 16 males (64%), 9 females (36%) | **Consumed sick or dead birds: 7 (39%) Touched sick or dead birds: 16 (70%) Cared for sick or dead birds: 9 (45%) Butchered sick or dead birds: 3 (17%) Sick or dead birds in household: 18 (85%) Contacted another human H5N1 case: 3 (16%)  ≥1 direct exposures: 25 (100)  Indirect plus ≥1 direct exposures: 12 (48%) (Indirect exposures: the presence of sick or dead birds in the patient’s neighborhood)** | Backyard/Neighborhood Poultry, Commercial Poultry/Swine/Beef Facilities, Poultry-Contaminated Environments | **Mucosa (oral/nasal/conjunctival) Inhalation (respiratory) Gastrointestinal** | RT-PCR | Not stated | Not stated |
| Shu | 2006 | Lethal avian influenza A (H5N1) infection in a pregnant woman in Anhui Province, China | New England Journal of Medicine | China | Case report | 1 (plus fetus) | Backyard Poultry Owners/Poultry Consumers | Not stated | 1 female (pregnant, 4 months) | Chickens and ducks in her household had become ill and had died during October. From October 25 through October 30, the patient had been **actively involved in slaughtering and defeathering sick poultry before they were cooked for family consumption.** | Backyard/Neighborhood Poultry, Poultry Preparation Environments | **Mucosa (oral/nasal/conjunctival) Inhalation (respiratory) Gastrointestinal** | RT-PCR | Not stated | Not stated |
| To | 2016 | Ongoing transmission of avian influenza A viruses in Hong Kong despite very comprehensive poultry control measures: A prospective seroepidemiology study | Journal of Infection | China | Prospective cohort study (serologic) | 24 seropositive (7 seroconversion)  Of seropositive:  22 LPM workers (6 seroconversions)  2 SH workers (1 seroconversion) | Live Bird Market Workers, Dairy or Swine Workers | Not stated | Not stated | **Exposure to live poultry as only stated exposure** **Chicken** 2013 LPM workers: 30 (100%) 2013 SH workers: 1 (1.4%) 2014 LPM workers: 45 (100%) 2014 SH workers: 1 (3.7%) **Duck** 2013 LPM workers: 1 (3.3%) 2013 SH workers: 0 (0%) 2014 LPM workers: 11 (24.4%) 2014 SH workers: 0 (0%) **Pigeon** 2013 LPM workers: 8 (26.7%) 2013 SH workers: 0 (0%) 2014 LPM workers: 16 (35.6%) 2014 SH workers: 0 (0%) **Goose** 2013 LPM workers: 0 (0%) 2013 SH workers: 0 (0%) 2014 LPM workers: 5 (11.1%) 2014 SH workers: 0 (0%)  **Pig or cattle also potential exposure (1 SH worker seroconverted)** | Live Bird Markets, Commercial Poultry/Swine/Beef Facilities | **Mucosa (oral/nasal/conjunctival) Inhalation (respiratory)** | Haemagglutination inhibition assay | A study participant is considered to have seroconversion if there is a ≥4-fold increase in the HI titer from 2013 to 2014.  For H5N1 virus, none of the serum samples from LPM/ SH workers collected in 2013 had an HI titer of ≥40.   If an HI titer of ≥40 was used as the seropositivity cutoff, H5N1 seropositive rate was significantly higher for LPM workers than that of SH workers in 2014 (37.8% [17/45] vs 3.7% [1/27])  If a HI titer of ≥80 was used as the seropositivity cutoff, there was a trend towards higher H5N1 seropositive rate among LPM workers in 2014 than in 2013 (11.1% [5/45] vs 0% [0/30])  **Seroconversion to H5N1 for those that had paired serum samples available: 6/10 LPM workers, 1/12 SH workers** | Not stated |
| Ungchusak | 2005 | Probable person-to-person transmission of avian influenza A (H5N1) | New England Journal of Medicine | Thailand | Case investigation/series | 3 (all confirmed H5N1) family members | Backyard Poultry Owners/Poultry Consumers, Residents of Poultry-Contaminated Environments, Close Contacts of H5N1 Cases | 11 years old (daughter), 26 years old (mother), 32 years old (aunt) | 3 females | The last of the free-ranging household chickens died on August 29 or 30, after progressive illness and death among the flock during the preceding weeks. The index patient was not known to have had direct contact with the sick or dying birds, but **she played and slept in the area under the elevated house, where the chickens were also often present**. The aunt buried the last five chickens on August 29 or 30, using plastic bags on her hands for protection.  From the time the index patient became ill until the arrival of her mother at the hospital, **the aunt provided much of her care, including bedside care for 12 or 13 hours** on September 7. **The mother then provided bedside care for the next 16 to 18 hours**, and nurses later reported that she **sat on the bed, hugged and kissed her daughter, and wiped secretions from her mouth**. | Backyard/Neighborhood Poultry, Poultry-Contaminated Environments, Healthcare/Bedside Care | **Mucosa (oral/nasal/conjunctival) Inhalation (respiratory)** | RT-PCR | Convalescent-phase specimen obtained from the aunt on day 21 was positive for H5N1 antibodies (negative for index case). | The aunt buried the last five chickens on August 29 or 30, using plastic bags on her hands for protection.  Mother had direct, unprotected exposure to her critically ill daughter. |
| Uyeki | 2024 | Highly Pathogenic Avian Influenza A(H5N1) Virus Infection in a Dairy Farm Worker | New England Journal of Medicine | United States (Texas) | Case report | 1 (confirmed H5N1 case) | Dairy or Swine Workers | Not stated (adult) | Not stated | The worker reported **direct and close exposure to dairy cows that appeared to be well and with sick cows that showed the same signs of illness** as cows at other dairy farms in the same area of northern Texas with confirmed HPAI A(H5N1) virus infection (e.g., decreased milk production, reduced appetite, lethargy, fever, and dehydration). The worker reported wearing gloves when working with cows but did not use any respiratory or eye protection. | Dairy Farms | **Mucosa (oral/nasal/conjunctival) Inhalation (respiratory)** | RT-PCR, sequencing | Not stated | The worker reported wearing gloves when working with cows but did not use any respiratory or eye protection. |
| Vong | 2009 | Risk factors associated with subclinical human infection with avian influenza A (H5N1) virus--Cambodia, 2006 | Journal of Infectious Diseases | Cambodia | Cross-sectional cohort study (serologic) with nested case control | 674 villagers (7 participants seropositive for H5N1) | Backyard Poultry Owners/Poultry Consumers, Residents of Poultry-Contaminated Environments | Median age of all participants: 21.5 years (range: 4 months–89 years)  **Median age of 7 participants seropositive for H5N1: 12 years (range: 4-18 years)** | Seropositive participants: 6 males, 1 female | In matched case-control study (7 cases matched to 24 controls), H5N1-seropositive persons were more likely than control subjects to report **bathing or swimming in household ponds** (71.4% vs. 20.8%; **matched OR, 11.3 [95% CI, 1.3– 102.2]**; P=0.03).  **Gathering poultry and placing poultry in cages** was associated with H5N1 virus infection; **matched OR, 5.8 (95% CI, 0.98–34.12) (P=0.05)**  There was a trend toward statistical significance for **environmental contamination; H5N1-seropositive persons’ households had ponds as the only water source, whereas control subjects’ households did not (85.7% vs. 41.7%; P=0.08)**.   In addition, H5N1-seropositive persons were more likely than control subjects to have **cleaned and/or removed feces from poultry cages** (P=0.09). Matched OR: **5.0 (95% CI, 0.69–36.33)** Touched and/or collected eggs: matched OR: 2.5 (95% CI: 0.23–26.02) Helped prepare poultry for food: matched OR: 1.1 (95% CI: 0.20–6.34) Slaughtered and/or bled poultry: matched OR: 2.5 (95% CI: 0.31–10.8) Removed internal organs from poultry: matched OR: 1.5 (95% CI: 0.26–8.68) Cut and/or washed internal organs: matched OR: 1.5 (95% CI: 0.26–8.68) Cut and/or washed poultry meat: matched OR: 2.0 (95% CI: 0.27–14.9) Defeathered poultry that died of illness: matched OR: 3.1 (95% CI: 0.28–34.87) | Backyard/Neighborhood Poultry, Poultry-Contaminated Environments, Poultry Preparation Environments | **Mucosa (oral/nasal/conjunctival) Inhalation (respiratory) Gastrointestinal** | Microneutralization assay, modified haemagglutination inhibition assay, Western blot assay confirmation | Serum samples that had titers ≥1:80 in duplicate microneutralization assays were considered to be positive for anti-H5 neutralizing antibody. Serum samples that tested positive in the microneutralization assay were also tested by Western blot assay. Serum samples that tested positive by both microneutralization and Western blot assays were considered to be positive for anti-H5 antibodies | Used personal protective equipment when handling poultry or poultry products: 0 case patients reported using plastic bags over hands, gloves, or wearing a cotton mask while 1 control patient reported using plastic bags over hands, 1 reported using gloves, and 2 reported wearing a cotton mask.  3 case patients reported washing thier hands with soap before eating, compared to 17 controls. 2 case patients reported washing thier hands with soap after handling poultry, compared to 11 controls. |
| Wang | 2008 | Probable limited person-to-person transmission of highly pathogenic avian influenza A (H5N1) virus in China | The Lancet | China | Case investigation/series | 2 (both positive for H5N1) | Close Contacts of H5N1 Cases | Index case: 24 years Case 2: 52 years | 2 males | Case 2 did not have any known contact with ill individuals except for his son during the 2 weeks before the onset of illness. After the index case became ill, the patient had **close contact** with him five times, including **eating dinner together, providing care, and attending his funeral**. He provided **unprotected bedside hospital care** for the index case between Nov 27 and 29. The longest continuous time he spent caring for his son was 20 h. **During this period, the index case had high fever (40·0ºC), frequent coughing, extensive sputum production, and frequent episodes of watery diarrhoea. Case two had helped to change his son’s soiled clothes and bedsheets, and had cleaned the toilet that had been used to dispose of diarrhoeal stool and a spittoon that contained copious sputum.** | Healthcare/Bedside Care | **Mucosa (oral/nasal/conjunctival) Inhalation (respiratory)** | RT-PCR, viral culture | Not stated | Case 2 did not use personal protective equipment until after H5N1 had been confirmed in the index case late on Dec 1. Then wore surgical mask and long-sleeved gown Nov 29th, then gloves, gown, hair cover, N95 respirator, eye protection on Dec 2. |
| World Health Organization | 2006 | Human cases of influenza A(H5N1) infection in eastern Turkey, December 2005-January 2006 | Weekly Epidemiological Record | Turkey | Case investigation/series | 10 (all confirmed H5N1) | Backyard Poultry Owners/Poultry Consumers, Residents of Poultry-Contaminated Environments | All cases occurred among children aged 3–15 years (median, 8.5 years; mean, 8.9 years) | 5 males, 5 females | Family A kept **8 chickens and 1 turkey**. On 20 December, 1 of the household’s chickens fell ill. On 25 December 2005, **Case 1 and Case 2 slaughtered 1 of the chickens**. Case 3 was reportedly not directly involved in the process but **was present in the house**.  Family B kept **25 chickens and 2 domesticated pigeons** that were being raised as pets. On 26 December, **the chickens became ill and were slaughtered** over the next 3 days. Case 4, a 9-year-old girl, **helped her mother slaughter the sick chickens**; she developed symptoms on 29 December 2005. On 30 December, 1 of her brothers (Case 5), aged 3 years, became ill. The **boy had not been present when the chickens were slaughtered but had played with the pet pigeons**.  Family C, residing in the district capital, consisted of 8 members (6 children and their parents). The family kept **4 ducks and 4 pet pigeons**. One **duck became ill** on 4 January. On this date, Case 6, a 14-year-old girl, **helped her mother slaughter and prepare ducks for consumption**. She and her younger brother (Case 7), aged 5 years, had onset of illness on 8 January. Case 7 **did not contribute to the preparation of the ducks but he had played with the family pigeons**.  Case 8: the boy’s family had a **small flock of poultry**; since December, **widespread death** among free-ranging poultry had been reported in the immediate surroundings  Case 9: He resided in the village of Çetenli, near the district capital. The boy’s family did not raise poultry but **widespread death among free-ranging poultry had been reported in the village, including among his neighbour’s poultry flock**.  Case 10: Family kept 22 hens; these had started dying and were slaughtered by family members. The girl cared for a sick pet chicken and also helped her mother prepare the slaughtered poultry for consumption | Backyard/Neighborhood Poultry, Poultry-Contaminated Environments, Poultry Preparation Environments | **Mucosa (oral/nasal/conjunctival) Inhalation (respiratory) Gastrointestinal** | RT-PCR, serology | RT-PCR, serology | Poultry is usually kept and fed outside, but during the winter poultry is brought indoors at night to protect it from the cold. Because of the severe cold spell, many families, among them families A, B and C, sheltered their poultry indoors. This, combined with the cramped living conditions of the rural population, considerably increased the opportunity for contact between humans and poultry. |
| World Health Organization | 2008 | Human cases of avian influenza A (H5N1) in North-West Frontier Province, Pakistan, October-November 2007 | Weekly Epidemiological Record | Pakistan | Case investigation/series | 4 (confirmed H5N1 cases), 1 suspected | Close Contacts of H5N1 Cases, Commercial Poultry Facility Workers | Case 1: 25 years Case 2: 22 years Case 3: 27 years Case 4: 32 years Case 5: 33 years | 5 males | **Case 1**: a laboratory-confirmed outbreak of highly pathogenic avian influenza A(H5N1) among poultry occurred at a breeding farm. A **culling operation** was carried out. One of the 13 people performing the culling was a 25-year-old livestock production officer (Case 1). During this operation, Case 1 **handled dead, sick and healthy chickens without using personal protective equipment**. During culling, both live and dead poultry were collected and put into large bags until each bag was full; each bag was then tied and placed in a deep pit for burial. Case 1 **gathered chickens** and placed them in the bag and also held the bag open while others put chickens inside  **Case 2:** Case 2 had had **close prolonged contact** with Case 1. They ate meals together and slept 2 nights in the same bedroom at the family’s Peshawar home starting on 2 November. Case 2 had had prolonged visits on 5 November and 7 November with Case 1 during Case 1’s hospitalization. Case 2 had no history of exposure to sick or dead poultry.   **Case 3:** Case 3 had had **close prolonged contact** with both Case 2 and Case 1. He was the primary caregiver for Case 2 during his hospitalization for severe acute respiratory infection during 14–19 November. Case 3 had also shared a bedroom with Case 1 during 2–4 November and had visited Case 1 during his hospitalization. Case 3 had no history of exposure to sick or dead poultry.  **Case 5:** He was asymptomatic but clinical specimens were collected from him owing to the close and prolonged contact with his ill brothers | Commercial Poultry/Swine/Beef Facilities, Healthcare/Bedside Care | **Mucosa (oral/nasal/conjunctival) Inhalation (respiratory)** | **Case 1:** microneutralization assays demonstrated antibody titres against influenza A(H5N1) virus of 1:2560; samples from 8 December showed titres of 1:1280. Western blot assay was also positive for influenza A(H5N1) virus **Case 2:** No lab tests performed **Case 3:** The throat swab was positive for H5 by real-time reverse transcriptase–polymerase chain reaction (RT–PCR); influenza A(H5N1) virus was isolated from the specimen at a WHO influenza A/H5 reference laboratory.  **Case 4:** serology tests were positive and showed that seroconversion had occurred between the time of the first and second serum sample. The initial specimen yielded a microneutralization H5 antibody titre of 1:10; a later specimen yielded a positive microneutralization test with an antibody titre of 1:320 and a positive western blot assay. **Case 5:** Initial testing at the National Institute of Health yielded positive results for H5 RT–PCR on a throat swab collected on 29 November. When serum specimens were tested by microneutralization assay, a specimen collected on 8 December yielded an H5 antibody titre of 1:320 and a positive western blot assay | **Case 1:** microneutralization assays demonstrated antibody titres against influenza A(H5N1) virus of 1:2560; samples from 8 December showed titres of 1:1280. Western blot assay was also positive for influenza A(H5N1) virus **Case 3:** The throat swab was positive for H5 by real-time reverse transcriptase–polymerase chain reaction (RT–PCR); influenza A(H5N1) virus was isolated from the specimen at a WHO influenza A/H5 reference laboratory.  **Case 4:** serology tests were positive and showed that seroconversion had occurred between the time of the first and second serum sample. The initial specimen yielded a microneutralization H5 antibody titre of 1:10; a later specimen yielded a positive microneutralization test with an antibody titre of 1:320 and a positive western blot assay. **Case 5:** When serum specimens were tested by microneutralization assay, a specimen collected on 8 December yielded an H5 antibody titre of 1:320 and a positive western blot assay | Case 1 handled dead, sick and healthy chickens without using personal protective equipment. Close contact to sick index case (by others). |
| Yu | 2007 | Human influenza A (H5N1) cases, urban areas of People's Republic of China, 2005-2006 | Emerging Infectious Diseases | China | Case investigation/series | 6 (all H5N1 confirmed cases) | Backyard Poultry Owners/Poultry Consumers, Live Bird Market Visitors | Median age: 30 years (range 21 to 41 years) | 3 males, 3 females | **Case 1:** Factory worker. **Visited a wet market nearly every day the week before illness onset** but did not purchase poultry or poultry products. **Case 2:** **Worked at a stall that she owned at a wet market, selling groceries and eggs. Her stall was ≈20 m away from stalls selling and slaughtering live poultry.** **Case 3:** He **visited up to 9 wet markets for 10–40 min every day** during the week before illness onset. At 1 wet market, he spent most of his time in a sauce store that was ≈5 m away from stalls where **poultry were slaughtered** and sold. **Case 4: Visited a wet market every day** to buy fresh food, **including freshly slaughtered chickens**, 1 wk before illness onset.  **Case 5:** Two wk before illness onset, he traveled to his hometown in the rural area of Enshi to attend the funeral of his uncle, who died of esophageal cancer. The man stayed there for 6 d, visited his parents’ home, **where healthy backyard poultry were kept (none became sick or died), and visited a wet market**. One wk before onset, he traveled back to his workplace in Wuhan, bringing 200 eggs from his hometown. **Case 6:** Two d before illness onset, he **visited a wet market once**, but he did not purchase any poultry or poultry products. One wk before onset, his **wife visited the same market and brought a live chicken that was slaughtered at the market.** | Backyard/Neighborhood Poultry, Live Bird Markets | **Mucosa (oral/nasal/conjunctival) Inhalation (respiratory)** | Not stated. A confirmed case of influenza (H5N1) was defined according to World Health Organization case definitions. | Not stated | Not stated |
| Yu | 2006 | The first confirmed human case of avian influenza A (H5N1) in Mainland China | The Lancet | China | Case report | 2 (both positive for H5N1) | Backyard Poultry Owners/Poultry Consumers | 12 years old, 9 years old | 1 male, 1 female | Like other families in the rural area, backyard poultry-raising is commonly practised; before the outbreak, the family had **22 chickens and five ducks kept in cages in a confined area adjacent to the bathroom and toilets within the house**. Because of the National Day holidays (from Oct 1 to Oct 7), children were in the house for a longer period of time. It was noted in retrospect that a few chickens and ducks had begun to die in the village from Sept 16. Between Oct 6 and Oct 12, up to six birds in the affected household died per day; by Oct 19, only one chicken and one duck remained alive. The mother **cooked the dead and dying birds for consumption by the family** | Backyard/Neighborhood Poultry, Poultry Preparation Environments | **Mucosa (oral/nasal/conjunctival) Inhalation (respiratory) Gastrointestinal** | MN and HI. The boy’s samples, collected on days 8, 17, and 22 after the onset of the illness, showed a 4-fold or greater rise in antibody titre. Throat swabs for RT-PCR were, however, negative.  Female was negative for H5N1 (samples isolated from remaining surviving chicken in household) | The boy’s samples, collected on days 8, 17, and 22 after the onset of the illness, showed a 4-fold or greater rise in antibody titre. | Because of the National Day holidays (from Oct 1 to Oct 7), children were in the house for a longer period of time |
| Yu | 2024 | Severe Avian Influenza A H5N1 Clade 2.3.4.4b Virus Infection in a Human with Continuation of SARS-CoV-2 Viral RNAs | Transboundary and Emerging Diseases | China | Case report | 1 (confirmed H5N1 case) | Backyard Poultry Owners/Poultry Consumers | 53 years old | 1 female | The patient did not have a recent history of travelling outside her residential city, visiting live poultry markets, or contacting with individuals with similar symptoms in the 10 days prior to her illness onset. The patient raised poultry (about 20 chickens and 10 ducks) in a semi-open poultry house in her family backyard, where wild birds would frequently visit for food. **She fed and cleaned the poultry house without using any personal protective equipment. Prior to the onset of her illness, the patient administered antibiotics to the sick poultry with her bare hands.** | Backyard/Neighborhood Poultry | **Mucosa (oral/nasal/conjunctival) Inhalation (respiratory)** | RT-PCR | Not stated | Epidemiological investigation of the patient suggested that the patient might acquire infection from the poultry raised in her family backyard, as she had a **history of direct contact with the poultry without any personal protective equipment** before the onset of her illness. |
| Zhou | 2009 | Risk factors for human illness with avian influenza A (H5N1) virus infection in China | Journal of Infectious Diseases | China | Case-control | **28 cases (confirmed H5N1 cases)** and 134 controls | Backyard Poultry Owners/Poultry Consumers, Commercial Poultry Facility Workers, Live Bird Market Workers | Cases: median age 29 years (range: 6–62 years) | Cases: 13 males (46%), 15 females (54%) | **Occupational poultry exposure: OR: 13.1 (95% CI: 1.4–125.4) Raise backyard poultry: OR: 4.5 (95% CI: 1.1–17.5) Poultry present inside house: OR: 9.7 (95% CI: 1.8–53.3) Raise waterfowl: OR: 6.4 (95% CI: 1.6–26.3)** Exposures to healthy-appearing poultry **Direct contact: OR: 3.3 (95% CI: 1.0–10.4)** Exposures to sick and/or dead poultry **Direct contact: OR: 34.7 (95% CI: 4.3–276.9) Only indirect contact (within 1 m): OR: 11.3 (95% CI: 2.2–58.5)** Wet poultry market exposure **Visited wet poultry market: OR: 3.1 (1.2–7.9) Visited wet poultry market and witnessed poultry slaughtering at market: OR: 5.0 (1.7–14.9)** Frequency of visits to wet poultry market within 2 weeks before illness onset **1–5 times: OR: 2.8 (95% CI: 0.9–8.1)  6–10 times: OR: 7.6 (95% CI: 1.1–53.7) >10 times: OR: 5.8 (95% CI: 1.2–28.6)** | Backyard/Neighborhood Poultry, Live Bird Markets Commercial, Poultry/Swine/Beef Facilities | **Mucosa (oral/nasal/conjunctival) Inhalation (respiratory)** | Viral isolation, RT-PCR, serological testing (microneutralization assay, modified hemagglutinin-inhibition assay) | A serum specimen with an influenza H5N1 neutralizing antibody titer of ≥1:80 was considered to be positive, with confirmation by the hemagglutinin-inhibition assay | Backyard poultry H5 vaccination: Vaccination coverage <80%: OR: 7.1 (95% CI: 1.6–31.6) Vaccination coverage ≥80%: OR: 4.0 (95% CI: 0.9–17.9)  Domestic waterfowl H5 vaccination: Vaccination coverage <80%: OR: 8.4 (95% CI: 1.6–45.1) |
| Zhu | 2025 | Human Cases of Highly Pathogenic Avian Influenza A(H5N1) - California, September-December 2024 | Morbidity and Mortality Weekly Report (MMWR) Supplements - Centers for Disease Control and Prevention (CDC) | United States (California) | Case investigation/series | 37 (36 confirmed, 1 suspected H5N1 case) | Dairy or Swine Workers | Median age 43 years (18–64 years) | Not stated | A majority (76%) worked as **milkers or cared for sick cows**.  A majority of patients (78%) reported using personal protective equipment (PPE) at work; 25 (68%) wore gloves, 20 (54%) used eye protection (13 reported wearing goggles), 12 (32%) reported wearing boots, and six (16%) wore gowns. No patients specifically reported wearing a respirator (e.g., an N95 mask) as recommended; however, 12 (32%) reported wearing other face coverings or face masks. | Dairy Farms | **Mucosa (oral/nasal/conjunctival) Inhalation (respiratory)** | Hemagglutinin (H) and genetic sequencing | Not stated | A majority of patients (78%) reported using personal protective equipment (PPE) at work; 25 (68%) wore gloves, 20 (54%) used eye protection (13 reported wearing goggles), 12 (32%) reported wearing boots, and six (16%) wore gowns. No patients specifically reported wearing a respirator (e.g., an N95 mask) as recommended; however, 12 (32%) reported wearing other face coverings or face masks. |
| World Health Organization | 2011 | Influenza A (H5N1) virus - Egypt | World Health Organization | Egypt | WHO press release | 4 (confirmed H5N1 cases) | Not stated | 20 years old, 2 years old, 55 years old, 1 year old | 1 male, 3 females | Investigations into the source of infection indicate that the cases had **exposure to sick and/or dead poultry suspected to have avian influenza**. | Not stated | **Mucosa (oral/nasal/conjunctival) Inhalation (respiratory)** | Not stated. Says confirmed by the Egyptian Central Public Health Laboratories, a National Influenza Center of the WHO Global Influenza Surveillance Network | Not stated | Not stated |
| World Health Organization | 2010 | Influenza A (H5N1) virus - Egypt | World Health Organization | Egypt | WHO press release | 5 (confirmed H5N1 cases) | Not stated | 53 years old, 1 year old, 10 years old, 30 years old, 13 years old | 4 males, 1 female | Investigations into the source of infection indicate that the cases had **exposure to sick and/or dead poultry.** | Not stated | **Mucosa (oral/nasal/conjunctival) Inhalation (respiratory)** | Not stated. Says confirmed by the Egyptian Central Public Health Laboratories, a National Influenza Center of the WHO Global Influenza Surveillance Network | Not stated | Not stated |
| World Health Organization | 2007 | Influenza A (H5N1) virus - Egypt | World Health Organization | Egypt | WHO press release | 1 (confirmed H5N1 case) | Not stated | 4 years old | 1 male | Initial investigations into the source of his infection indicate **exposure to dead poultry**. | Not stated | **Mucosa (oral/nasal/conjunctival) Inhalation (respiratory)** | Not stated. Says confirmed by the Egyptian Central Public Health Laboratory and by the WHO H5 Reference Laboratory, US Naval Medical Research Unit No.3 | Not stated | Not stated |
| World Health Organization | 2007 | Influenza A (H5N1) virus - Egypt | World Health Organization | Egypt | WHO press release | 2 (confirmed H5N1 cases) | Backyard Poultry Owners/Poultry Consumers | 2 years old, 15 years old | 2 females | Initial investigations into the source of her infection indicate **recent contact with backyard poultry**. | Backyard/Neighborhood Poultry | **Mucosa (oral/nasal/conjunctival) Inhalation (respiratory)** | Not stated. Says confirmed by the Egyptian Central Public Health Laboratory and by the WHO H5 Reference Laboratory, US Naval Medical Research Unit No.3 | Not stated | Not stated |
| World Health Organization | 2007 | Influenza A (H5N1) virus - Egypt | World Health Organization | Egypt | WHO press release | 1 (confirmed H5N1 case) | Not stated | 10 years old | 1 female | Investigations indicate that she had recently been **exposed to sick poultry.** | Not stated | **Mucosa (oral/nasal/conjunctival) Inhalation (respiratory)** | Not stated. Says confirmed by the Egyptian Central Public Health Laboratory and by the WHO H5 Reference Laboratory, US Naval Medical Research Unit No.3 | Not stated | Not stated |
| World Health Organization | 2007 | Influenza A (H5N1) virus - Egypt | World Health Organization | Egypt | WHO press release | 1 (confirmed H5N1 case) | Backyard Poultry Owners/Poultry Consumers | 4 years old | 1 female | The girl was **exposed to sick birds at her home** one week prior to the onset of symptoms. | Backyard/Neighborhood Poultry | **Mucosa (oral/nasal/conjunctival) Inhalation (respiratory)** | Not stated. Says confirmed by the Egyptian Central Public Health Laboratory and by the WHO H5 Reference Laboratory, US Naval Medical Research Unit No.3 | Not stated | Not stated |
| World Health Organization | 2023 | Human infection caused by avian influenza A(H5) - Ecuador | World Health Organization | Ecuador | WHO press release | 1 (confirmed H5N1 case) | Backyard Poultry Owners/Poultry Consumers, Residents of Poultry-Contaminated Environments | 9 years old | 1 female | On 9 January 2023, WHO was notified of a human infection caused by an avian influenza A(H5) virus. The case, a nine-year-old girl, living in a rural area in the province of Bolívar, Ecuador, **was in contact with backyard poultry, which was acquired a week before the onset of her symptoms**. She is currently hospitalized, in isolation, and is being treated with antivirals. This is the first reported case of human infection caused by avian influenza A(H5) virus in the Latin America and the Caribbean region. Work is ongoing to further characterize the virus.  According to the epidemiological investigation in response to the outbreak, a week before the onset of her symptoms, **the family acquired poultry which died without apparent cause on 19 December 2022. In addition, the epidemiological investigations revealed that several incidents of dead backyard poultry (chickens and ducks) have been reported from the same community where the family resided.** | Backyard/Neighborhood Poultry, Poultry-Contaminated Environments | **Mucosa (oral/nasal/conjunctival) Inhalation (respiratory)** | RT-PCR | Not stated | Not stated |
| World Health Organization | 2023 | Avian Influenza A H5N1 - United Kingdom of Great Britain and Northern Ireland | World Health Organization | England | WHO press release | 2 (confirmed H5N1 cases) | Commercial Poultry Facility Workers | Not stated | Not stated | In a poultry worker at a farm in England where poultry was infected with high pathogenicity avian influenza (HPAI) A(H5N1) viruses. Another detection was reported in a second individual **performing culling operations on the farm**. | Commercial Poultry/Swine/Beef Facilities | **Mucosa (oral/nasal/conjunctival) Inhalation (respiratory)** | Testing (not specified), genetic sequencing | Not stated | One poultry culler worked on the farm in early May using personal protective equipment (PPE). |
| World Health Organization | 2006 | 2006 - Iraq | World Health Organization | Iraq | WHO press release | 2 (1 confirmed, 1 suspected H5N1 infection) | Backyard Poultry Owners/Poultry Consumers | 15 years old, 39 years old | 1 male, 1 female | The Ministry of Health in Iraq has confirmed the country’s first case of human infection with the H5N1 avian influenza virus. The case occurred in a 15-year-old girl who died on 17 January following a severe respiratory illness. Her symptoms were compatible with a diagnosis of H5N1 avian influenza.  The girl’s **39-year-old uncle, who cared for her during her illness, developed symptoms on 24 January and died of a severe respiratory disease** on 27 January.  Both patients resided in the town of Raniya near Sulaimaniyah in the northern part of the country, close to the border with Turkey. **Poultry deaths were recently reported in their neighbourhood**, but H5N1 avian influenza has not yet been confirmed in birds in any part of the country. Poultry samples have been sent for testing at an external laboratory.  **A history of exposure to diseased birds has been found for the girl. The uncle’s source of infection is under investigation.** | Backyard/Neighborhood Poultry | **Mucosa (oral/nasal/conjunctival) Inhalation (respiratory)** | Not specified. WHO confirmed. Preliminary laboratory confirmation was provided by a US Naval Medical Research Unit located in Cairo, Egypt. | Not stated | Not stated |
| World Health Organization | 2022 | Avian Influenza A (H5N1) - Spain | World Health Organization | Spain | WHO press release | 2 (confirmed H5N1 cases) | Commercial Poultry Facility Workers | 19 years old, 27 years old | 2 males | The detection of influenza A(H5N1) in these individuals was **likely due to exposure to infected poultry or contaminated environments** (following an outbreak in poultry). | Commercial Poultry/Swine/Beef Facilities | **Mucosa (oral/nasal/conjunctival) Inhalation (respiratory)** | RT-PCR | Not stated | Not stated |
| World Health Organization | 2024 | Avian Influenza A(H5N1) - United States of America | World Health Organization | United States (Texas) | WHO press release | 1 (confirmed H5N1 case) | Dairy or Swine Workers | Over 18 years old | 1 male | Reported to be while working at a commercial dairy cattle farm and had a **history of exposure to dairy cattle (cows) presumed to be infected with influenza A(H5N1) virus**. | Dairy Farms | **Mucosa (oral/nasal/conjunctival) Inhalation (respiratory)** | RT-PCR and sequencing to confirm H5N1 | Not stated | Not stated |
| World Health Organization | 2023 | Human Infection caused by Avian Influenza A (H5N1) - Chile | World Health Organization | Chile | WHO press release | 1 (confirmed H5N1 case) | Residents of Poultry-Contaminated Environments | 53 years old | 1 male | According to preliminary findings of the epidemiological investigation of this human case, the most plausible route of transmission was through **environmental exposure**, **given the large number of dead sea mammals and wild birds found in the area close to the patient´s residence**. | Poultry-Contaminated Environments | **Mucosa (oral/nasal/conjunctival) Inhalation (respiratory)** | RT-PCR and genomic sequencing to confirm H5N1 | Not stated | Not stated |
| World Health Organization | 2022 | Avian Influenza A(H5N1) - United States of America | World Health Organization | United States (Colorado) | WHO press release | 1 (confirmed H5N1 case) | Commercial Poultry Facility Workers | Not stated | 1 male | The case was **involved in disposal and culling of poultry** at a farm where influenza A **(H5N1) virus was confirmed** in the poultry. The case developed fatigue on 20 April, during participation in **slaughtering poultry** from 18 to 22 April, at a commercial poultry facility in Colorado where influenza A (H5N1) virus had been confirmed in the poultry. | Commercial Poultry/Swine/Beef Facilities | **Mucosa (oral/nasal/conjunctival) Inhalation (respiratory)** | RT-PCR and sequencing to confirm H5N1 | Not stated | Not stated |
| World Health Organization | 2006 | Influenza A (H5N1) virus - Thailand | World Health Organization | Thailand | WHO press release | 1 (confirmed H5N1 case) | Backyard Poultry Owners/Poultry Consumers | 59 years old | 1 male | Possible H5N1 infection was suspected when relatives reported the sudden death, in the days prior to symptom onset, of **several fighting cocks raised by the farmer**. Local veterinary authorities noted a number of poultry outbreaks in the area. | Backyard/Neighborhood Poultry, Hunting/Cockfighting Environments | **Mucosa (oral/nasal/conjunctival) Inhalation (respiratory)** | Viral isolation | Not stated | Not stated |
| World Health Organization | 2004 | 2004 - Thailand | World Health Organization | Thailand | WHO press release | 1 (confirmed H5N1 case) | Not stated | 29 years old | 1 male | He developed symptoms on 13 February, was admitted to hospital on 20 February, and was discharged on 7 March. He gave a **history of exposure to diseased and dead chickens**. | Not stated | **Mucosa (oral/nasal/conjunctival) Inhalation (respiratory)** | Not stated. WHO confirmed | Not stated | Not stated |
| World Health Organization | 2004 | 2004 - Thailand | World Health Organization | Thailand | WHO press release | 1 (confirmed H5N1 case) | Backyard Poultry Owners/Poultry Consumers | 47 years old | 1 female | She fell ill with fever and cough on 3 February and was diagnosed with pneumonia on 20 February. Hospital discharge followed 5 days later. She had **exposure to diseased and dead chickens at her home** in January. | Backyard/Neighborhood Poultry | **Mucosa (oral/nasal/conjunctival) Inhalation (respiratory)** | Not stated. WHO confirmed | Not stated | Not stated |
| World Health Organization | 2004 | 2004 - Thailand | World Health Organization | Thailand | WHO press release | 2 (confirmed H5N1 cases) | Not stated | 2 years old, 27 years old | 1 male, 1 female | Investigations of the two cases have identified **contact with sick or dead chickens**. | Not stated | **Mucosa (oral/nasal/conjunctival) Inhalation (respiratory)** | Not stated. WHO confirmed | Not stated | Not stated |
| World Health Organization | 2004 | 2004 - Thailand | World Health Organization | Vietnam | WHO press release | 4 (2 confirmed H5N1 cases, 2 suspected cases) | Backyard Poultry Owners/Poultry Consumers, Residents of Poultry-Contaminated Environments, Close Contacts of H5N1 Cases | 31 years old, 23 years old, 30 years old, 28 years old | 1 male, 3 females | The family members gathered in late December to prepare for the man’s wedding on 3 January. Both the man and one sister are **reported to have handled ducks while preparing a meal** on 4 January. However, the investigation failed to reveal any direct contact with poultry for the man’s other sister and his wife.  In the absence of evidence of direct exposure to poultry in these two cases, WHO epidemiologists are considering various alternative explanations. **Both sisters are known to have provided health care for their brother, prior to his hospitalization, and would thus have had opportunities for close exposure. Direct human-to-human transmission following this close exposure is thus one possible explanation.**  However, **contact with an infected bird, or some other environmental source of the virus, is another possible route of infection that has not been ruled out**. Outbreaks of H5N1 in poultry are widespread in Vietnam. Large amounts of the virus are excreted in bird droppings, and can survive for some time in the environment. | Backyard/Neighborhood Poultry, Poultry-Contaminated Environments, Poultry Preparation Environments, Healthcare/Bedside Care | **Mucosa (oral/nasal/conjunctival) Inhalation (respiratory)** | Not specified. WHO confirmed (laboratory tests have confirmed H5N1 infection in the sisters) | Not stated | Not stated |
| World Health Organization | 2025 | Avian Influenza A(H5N1) - Cambodia | World Health Organization | Cambodia | WHO press release | 11 (confirmed H5N1 cases) | Backyard Poultry Owners/Poultry Consumers | 6 cases aged 18-65 years old, 3 cases <5 years old, 2 cases older than 5 but less than 18 years old | 7 males, 4 females | All cases had **exposure – handling or culling - of sick poultry, often kept in backyards**.  **Exposure to sick poultry: 8 cases Exposure to chickens: 1 case Handling and culling chickens: 1 case Exposure to dead chickens: 1 case** | Backyard/Neighborhood Poultry | **Mucosa (oral/nasal/conjunctival) Inhalation (respiratory)** | Not specified. WHO confirmed. | Not stated | Not stated |
| World Health Organization | 2024 | Avian Influenza A(H5N1) - Cambodia | World Health Organization | Cambodia | WHO press release | 1 (confirmed H5N1 case) | Backyard Poultry Owners/Poultry Consumers | 15 years old | 1 female | According to early investigations, there were reports of dead poultry in the village about five days before the patient’s onset of illness. The patient's family was given some of these chickens for consumption and the girl was **exposed to the chicken while preparing food**. | Poultry Preparation Environments | **Mucosa (oral/nasal/conjunctival) Inhalation (respiratory)** | qRT-PCR (nasopharyngeal and oropharyngeal swab specimens), sequencing | Not stated | Not stated |
| World Health Organization | 2024 | Avian Influenza A(H5N1) - Vietnam | World Health Organization | Vietnam | WHO press release | 1 (confirmed H5N1 case) | Hunters | 21 years old | 1 male | Initial results from the case investigation revealed that during the second and third weeks of February 2024, **the case went bird hunting**. Between that time and the onset of illness, no contact with dead or sick poultry nor contact with anyone exhibiting similar symptoms was reported. | Hunting/Cockfighting Environments | **Mucosa (oral/nasal/conjunctival) Inhalation (respiratory)** | RT-PCR, genomic sequencing | Not stated | Not stated |
| World Health Organization | 2023 | Avian Influenza A(H5N1) - Cambodia | World Health Organization | Cambodia | WHO press release | 2 (confirmed H5N1 cases) | Backyard Poultry Owners/Poultry Consumers | 1 in 20-25 years age group, 1 less than 5 | 2 females | Epidemiological investigation shows **both cases had exposure to backyard birds, which were reported to be sick, with some having died, over the prior month**.  Epidemiological investigation shows both cases had exposure to backyard birds which were reported to be sick and dead, over the past month. No epidemiological linkage of these cases has yet been confirmed other than that they both resided in the same village. | Backyard/Neighborhood Poultry | **Mucosa (oral/nasal/conjunctival) Inhalation (respiratory)** | RT-qPCR, confirmation by the Institut Pasteur du Cambodge | Not stated | Not stated |
| World Health Organization | 2020 | Avian Influenza A(H5N1) - Lao People’s Democratic Republic | World Health Organization | Lao | WHO press release | 1 (confirmed H5N1 cases) | Backyard Poultry Owners/Poultry Consumers | 1-year-old | 1 female | Upon further investigation, there were **domestic poultry at the residence**. There was no travel history of the family 14 days prior to symptom onset in the case. | Backyard/Neighborhood Poultry | **Mucosa (oral/nasal/conjunctival) Inhalation (respiratory)** | RT-PCR | Not stated | Not stated |
| World Health Organization | 2012 | Avian Influenza A(H5N1) - Vietnam | World Health Organization | Vietnam | WHO press release | 1 (confirmed H5N1 case) | Not stated | 31 years old | 1 male | Epidemiological investigation indicate that the man was involved in the **slaughter and consumption of sick poultry**. | Poultry Preparation Environments | **Mucosa (oral/nasal/conjunctival) Inhalation (respiratory) Gastrointestinal** | Not stated. Laboratory tests were confirmed by the Pasteur Institute Ho Chi Minh City, a WHO National influenza Centre. | Not stated | Not stated |
| World Health Organization | 2011 | Avian Influenza A(H5N1) - Cambodia | World Health Organization | Cambodia | WHO press release | 1 (confirmed H5N1 case) | Backyard Poultry Owners/Poultry Consumers | 7 years old | 1 female | **There have been reports of poultry die off in her village and the case is reported to have had exposure to sick poultry.** | Backyard/Neighborhood Poultry | **Mucosa (oral/nasal/conjunctival) Inhalation (respiratory)** | Not stated. WHO confirmed | Not stated | Not stated |
| World Health Organization | 2010 | Avian Influenza A(H5N1) - Cambodia | World Health Organization | Cambodia | WHO press release | 1 (confirmed H5N1 case) | Backyard Poultry Owners/Poultry Consumers | 27-years old | 1 male | **The patient prepared and consumed sick poultry in the 7 days before onset of symptoms.** | Poultry Preparation Environments | **Mucosa (oral/nasal/conjunctival) Inhalation (respiratory) Gastrointestinal** | Not stated. Laboratory tests were confirmed by the National Influenza Centre, the Institute Pasteur in Cambodia. | Not stated | Not stated |
| World Health Organization | 2009 | Avian Influenza A(H5N1) - Vietnam | World Health Organization | Vietnam | WHO press release | 1 (confirmed H5N1 case) | Not stated | 3 years old | 1 male | **Investigations into the source of infection indicated a history of close contact with sick and dead poultry prior to the onset of symptoms.** | Not stated | **Mucosa (oral/nasal/conjunctival) Inhalation (respiratory)** | Not stated. WHO confirmed (National Institute of Hygiene and Epidemiology). | Not stated | Not stated |
| World Health Organization | 2009 | Avian Influenza A(H5N1) - Vietnam | World Health Organization | Vietnam | WHO press release | 1 (confirmed H5N1 case) | Not stated | 8 years old | 1 female | **The case is known to have had recent contact with sick and dead poultry prior to the onset of her illness.** | Not stated | **Mucosa (oral/nasal/conjunctival) Inhalation (respiratory)** | Not stated. WHO confirmed (National Institute of Hygiene and Epidemiology). | Not stated | Not stated |
| World Health Organization | 2008 | Avian Influenza A(H5N1) - Indonesia | World Health Organization | Indonesia | WHO press release | 2 (confirmed H5N1 cases) | Backyard Poultry Owners/Poultry Consumers, Residents of Poultry-Contaminated Environments | 38 years old, 20 years old | 2 males | The first case, a 38 year old male from Tangerang Municipality, Banten Province developed symptoms on 4 July 2008, was hospitalized on 9 July and died on 10 July. **There were free roaming poultry throughout his neighbourhood, including a commercial poultry pen owned by a neighbour.**  The second case, a 20 year old male from Tangerang District, Banten Province developed symptoms on 20 July, was hospitalized on 29 July, and died on 31 July. Reports indicate that **chickens from the case's household had died in the week preceding the onset of his symptoms and that he had slaughtered and consumed some of his stock during this period.** | Backyard/Neighborhood Poultry, Commercial Poultry/Swine/Beef Facilities, Poultry-Contaminated Environments, Poultry Preparation Environments | **Mucosa (oral/nasal/conjunctival) Inhalation (respiratory) Gastrointestinal** | Not stated. Laboratory tests were confirmed by the Ministry of Health. | Not stated | Not stated |
| World Health Organization | 2008 | Avian Influenza A(H5N1) - China | World Health Organization | China | WHO press release | 1 (confirmed H5N1 case) | Not stated | 44 years old | 1 female | **The case had contact with sick and dead poultry prior to her illness.** | Not stated | **Mucosa (oral/nasal/conjunctival) Inhalation (respiratory)** | Not stated. Laboratory tests were confirmed by the Ministry of Health. | Not stated | Not stated |
| World Health Organization | 2008 | Avian Influenza A(H5N1) - China | World Health Organization | China | WHO press release | 1 (confirmed H5N1 case) | Not stated | 41 years old | 1 male | **The case had contact with sick and dead poultry prior to his illness.** | Not stated | **Mucosa (oral/nasal/conjunctival) Inhalation (respiratory)** | Not stated. Laboratory tests were confirmed by the Ministry of Health. | Not stated | Not stated |
| World Health Organization | 2008 | Avian Influenza A(H5N1) - Vietnam | World Health Organization | Vietnam | WHO press release | 1 (confirmed H5N1 case) | Not stated | 27-years old | 1 male | **The case had contact with sick and dead poultry prior to his illness.** | Not stated | **Mucosa (oral/nasal/conjunctival) Inhalation (respiratory)** | Not stated. WHO confirmed (National Institute of Hygiene and Epidemiology). | Not stated | Not stated |
| World Health Organization | 2008 | Avian Influenza A(H5N1) - Vietnam | World Health Organization | Vietnam | WHO press release | 1 (confirmed H5N1 case) | Residents of Poultry-Contaminated Environments | 34 years-old | 1 male | **The case had contact with sick and dead poultry prior to his illness. Poultry infected with H5N1 avian influenza were identified in the case's village following his illness.** | Poultry-Contaminated Environments | **Mucosa (oral/nasal/conjunctival) Inhalation (respiratory)** | Not stated. WHO confirmed (National Institute of Hygiene and Epidemiology). | Not stated | Not stated |
| World Health Organization | 2007 | Avian Influenza A(H5N1) - Indonesia | World Health Organization | Indonesia | WHO press release | 2 (confirmed H5N1 cases) | Residents of Poultry-Contaminated Environments | 27 years old, 18 years old | 1 male, 1 female | A 27-year-old woman from South Jakarta developed symptoms on 6 January 2007 and died on 12 January 2007. **Investigations into the source of her exposure found reports of bird deaths near her home in the days prior to symptom onset.**  The 18-year-old has now also been confirmed as infected with H5N1 avian influenza. He remains in hospital in a critical condition. **Investigations into the source of his infection indicate environmental exposure.** | Poultry-Contaminated Environments | **Mucosa (oral/nasal/conjunctival) Inhalation (respiratory)** | Not stated. Laboratory tests were confirmed by the Ministry of Health. | Not stated | Not stated |
| World Health Organization | 2006 | Avian Influenza A(H5N1) - Cambodia | World Health Organization | Cambodia | WHO press release | 1 (confirmed H5N1 case) | Backyard Poultry Owners/Poultry Consumers, Residents of Poultry-Contaminated Environments | 12 years old | 1 male | A team from the Ministry of Health, WHO, and the Pasteur Institute investigated the situation in the child’s village yesterday. **Numerous chicken deaths and some duck deaths were noted to have occurred in the neighbourhood in recent weeks. The child reportedly gathered dead chickens for distribution to village families for consumption.** | Backyard/Neighborhood Poultry, Poultry-Contaminated Environments | **Mucosa (oral/nasal/conjunctival) Inhalation (respiratory)** | Not stated. Laboratory tests were confirmed by the National Influenza Centre, the Institute Pasteur in Cambodia. | Not stated | Not stated |
| World Health Organization | 2006 | Avian Influenza A(H5N1) - Cambodia | World Health Organization | Cambodia | WHO press release | 1 (confirmed H5N1 case) | Backyard Poultry Owners/Poultry Consumers | 3 years old | 1 female | A team of officials from the Ministry of Health and WHO have investigated the situation in the remote village where the child lived. **Backyard poultry began dying in the village in February, and chicken deaths have continued. The child is known to have played with chickens, including some showing signs of illness.** | Backyard/Neighborhood Poultry | **Mucosa (oral/nasal/conjunctival) Inhalation (respiratory)** | Not stated. Laboratory tests were confirmed by the National Influenza Centre, the Institute Pasteur in Cambodia. | Not stated | Not stated |
| World Health Organization | 2006 | Avian Influenza A(H5N1) - Indonesia | World Health Organization | Indonesia | WHO press release | 1 (confirmed H5N1 case) | Residents of Poultry-Contaminated Environments | 4 years old | 1 male | A joint investigation involving provincial health and agricultural authorities found that **chickens had died in the boy’s neighbourhood in the days preceding symptom onset**. | Poultry-Contaminated Environments | **Mucosa (oral/nasal/conjunctival) Inhalation (respiratory)** | Not stated. Laboratory tests were confirmed by the Ministry of Health. | Not stated | Not stated |
| World Health Organization | 2006 | Avian Influenza A(H5N1) - Indonesia | World Health Organization | Indonesia | WHO press release | 1 (confirmed H5N1 case) | Residents of Poultry-Contaminated Environments | 27-years old | 1 female | Investigations carried out by local authorities found reports of **chicken deaths in the woman’s neighbourhood** four days prior to her onset of symptoms. | Poultry-Contaminated Environments | **Mucosa (oral/nasal/conjunctival) Inhalation (respiratory)** | Not stated. Laboratory tests were confirmed by the Ministry of Health. | Not stated | Not stated |
| World Health Organization | 2006 | Avian Influenza A(H5N1) - Indonesia | World Health Organization | Indonesia | WHO press release | 2 (confirmed H5N1 cases) | Residents of Poultry-Contaminated Environments | 27-years old, 22 years old | 2 females | The first case occurred in a 22-year-old woman who developed symptoms on 25 January and died on 10 February. **Her neighbours kept chickens. Samples from these chickens and from pet birds in a market near the woman’s home are being tested by Indonesia’s animal health authorities.**  The second case occurred in a 27-year-old woman who developed symptoms on 31 January and died on 10 February. **Deaths of chickens in her neighbourhood were reported four days prior to symptom onset** | Poultry-Contaminated Environments | **Mucosa (oral/nasal/conjunctival) Inhalation (respiratory)** | Not stated. Laboratory tests were confirmed by the Ministry of Health. | Not stated | Not stated |
| World Health Organization | 2006 | Avian Influenza A(H5N1) - China | World Health Organization | China | WHO press release | 1 (confirmed H5N1 case) | Backyard Poultry Owners/Poultry Consumers | 6 years old | 1 male | Initial investigation of the newly confirmed case has identified **recent poultry deaths in the family flock as the likely source of exposure**, though no poultry outbreaks have been officially reported in the area. | Backyard/Neighborhood Poultry | **Mucosa (oral/nasal/conjunctival) Inhalation (respiratory)** | Not stated. Laboratory tests were confirmed by the Ministry of Health. | Not stated | Not stated |
| World Health Organization | 2004 | 2004 - Vietnam | World Health Organization | Thailand | WHO press release | 1 (confirmed H5N1 case) | Residents of Poultry-Contaminated Environments | 13 years old | 1 male | Preliminary investigation has linked the case to **contact with diseased chickens near his home.** | Poultry-Contaminated Environments | **Mucosa (oral/nasal/conjunctival) Inhalation (respiratory)** | Not stated. Laboratory tests were confirmed by the Ministry of Public Health. | Not stated | Not stated |
| CIDRAP - Center for Infectious Disease Research & Policy Research and Innovation Office, University of Minnesota | 2007 | WHO confirms Myanmar's first human avian flu case | CIDRAP - Center for Infectious Disease Research & Policy Research and Innovation Office, University of Minnesota | Myanmar | News release | 1 (confirmed H5N1 case) | Residents of Poultry-Contaminated Environments | 7 years old | 1 female | The girl is from Kyaing Tone Township in Shan state near the Chinese border, according to a statement from the WHO. **Her illness was detected through routine surveillance following an H5N1 outbreak in poultry in the area in mid November**, the statement said.  She came down with a fever and headache on Nov 21, was hospitalized 6 days later, and has recovered, the report said.  Officials from Myanmar's health and livestock ministries, along with local WHO representatives, are investigating the girl's illness to determine the source. The WHO said **poultry deaths were reported near the girl's home the week before she got sick**. | Poultry-Contaminated Environments | **Mucosa (oral/nasal/conjunctival) Inhalation (respiratory)** | Not stated. Specimens were sent to the National Health Laboratory in Yangon, Myanmar's capital, and the National Institute of Health in Thailand, where the samples tested positive. The positive findings were also confirmed at the WHO collaborating center at the National Institute of Infectious Diseases in Tokyo, Japan. | Not stated | Not stated |
| CIDRAP - Center for Infectious Disease Research & Policy Research and Innovation Office, University of Minnesota | 2007 | Nigeria confirms its first human case of avian flu | CIDRAP - Center for Infectious Disease Research & Policy Research and Innovation Office, University of Minnesota | Nigeria | News release | 1 (confirmed H5N1 case) | Backyard Poultry Owners/Poultry Consumers | 22 years old | 1 female | "Last night our team of 13 scientists were able to conclusively identify the case of avian influenza," Nigerian Information Minister Frank Nweke said at a press conference. He said samples from the woman would be sent to other laboratories, including the World Health Organization (WHO), for further review.  **The woman fell ill after de-feathering and disemboweling an infected chicken.** She died Jan 17 | Poultry Preparation Environments | **Mucosa (oral/nasal/conjunctival) Inhalation (respiratory)** | Not stated. Specimens were sent to Nigerian authorities for confirmation, and then WHO. | Not stated | Not stated |
| U.S. Centers for Disease Control and Prevention | 2024 | CDC Confirms Second Human H5 Bird Flu Case in Michigan; Third Case Tied to Dairy Outbreak | U.S. Centers for Disease Control and Prevention | United States (Michigan) | News release | 1 (confirmed H5N1 case) | Dairy or Swine Workers | Not stated | Not stated | As with the previous two cases (one in Texas, one in Michigan), the person is a **dairy farm worker with exposure to infected cows**, making this another instance of probable cow-to-person spread...all three sporadic cases had **direct contact with infected cows**. Risk depends on exposure, and in this case, the relevant exposure is to infected animals. | Dairy Farms | **Mucosa (oral/nasal/conjunctival) Inhalation (respiratory)** | Specimens were collected from the patient; one of which was positive for influenza A(H5) virus using the CDC test at the state health department laboratory. The specimens were forwarded to CDC for further testing. They were received on May 29, and testing results that night confirmed A(H5) virus infection. Michigan was then notified of the results.  The designation of the influenza virus neuraminidase (the N in the subtype) is pending genetic sequencing at CDC. | Not stated | Not stated |
| Wisconsin Department of Health Services | 2024 | DHS Reports Presumptive Positive Human Case of Highly Pathogenic Avian Influenza (HPAI) in Wisconsin | Wisconsin Department of Health Services | United States (Wisconsin) | News release | 1 (confirmed H5N1 case) | Commercial Poultry Facility Workers | Not stated | Not stated | The Wisconsin Department of Health Services (DHS) has detected the first presumptive positive human case of Highly Pathogenic Avian Influenza A (H5N1), also known as bird flu, in Barron County. **The human case follows an infected flock of commercial poultry identified in Barron County. The person had exposure to the infected flock.** The case was identified through testing at the Wisconsin State Lab of Hygiene (WSLH) and is pending confirmation at CDC (Centers for Disease Control and Prevention). | Commercial Poultry/Swine/Beef Facilities | **Mucosa (oral/nasal/conjunctival) Inhalation (respiratory)** | Not stated | Not stated | Not stated |
| Iowa Department of Health and Human Services | 2024 | Iowa HHS Reports First Human Case of Highly Pathogenic Avian Influenza (HPAI) in Iowa, Risk Remains Very Low to Public | Iowa Department of Health and Human Services | United States (Iowa) | News release | 1 (confirmed H5N1 case) | Commercial Poultry Facility Workers | Not stated | Not stated | The Iowa Department of Health and Human Services (Iowa HHS) is reporting the first human case of avian influenza A(H5) in the state. **The individual was exposed to infected poultry while working with a commercial flock in northwest Iowa.** | Commercial Poultry/Swine/Beef Facilities | **Mucosa (oral/nasal/conjunctival) Inhalation (respiratory)** | The case was identified through testing at the State Hygienic Laboratory and confirmed by the Centers for Disease Control and Prevention (CDC). | Not stated | Not stated |
| U.S. Centers for Disease Control and Prevention | 2024 | CDC Confirms First Severe Case of H5N1 Bird Flu in the United States | U.S. Centers for Disease Control and Prevention | United States (Louisiana) | News release | 1 (confirmed H5N1 case) | Backyard Poultry Owners/Poultry Consumers | Not stated | Not stated | While an investigation into the source of the infection in Louisiana is ongoing, it has been determined that **the patient had exposure to sick and dead birds in backyard flocks**. This is the first case of H5N1 bird flu in the U.S. that has been linked to exposure to a backyard flock. | Backyard/Neighborhood Poultry | **Mucosa (oral/nasal/conjunctival) Inhalation (respiratory)** | Not stated. Lab confirmed by CDC. Genomic sequencing underway. | Not stated | Not stated |
| Central Nevada Health District | 2025 | The Central Nevada Health District is Actively Monitoring for Spread of H5N1 in Northern Nevada | Central Nevada Health District | United States (Nevada) | News release | 1 (confirmed H5N1 case) | Dairy or Swine Workers | Not stated | Not stated | The Central Nevada Health District has confirmed the state's first and only human case of avian influenza A (H5N1) virus in an adult who was **exposed to infected dairy cattle** **while working at a dairy farm in Churchill County.** | Dairy Farms | **Mucosa (oral/nasal/conjunctival) Inhalation (respiratory)** | The case was confirmed by the Central Nevada Health District. Genetic sequencing complete by the CDC. | Not stated | Not stated |
| Ohio Department of Health | 2025 | Ohio Reports First Human Case of Bird Flu | Ohio Department of Health | United States (Ohio) | News release | 1 (probable H5N1 case) | Commercial Poultry Facility Workers | Adult (age not stated) | 1 Male | The Ohio Department of Health is reporting the state’s first probable human case of influenza A(H5), also known as Highly Pathogenic Avian Influenza (HPAI), or bird flu. An adult male Mercer County **farm worker** who was **in contact with deceased commercial poultry was infected with the virus. Was conducting depopulation activities of commercial poultry.** The poultry worker in Ohio had respiratory symptoms and is home and recovering. This person participated in culling activities on a farm with infected poultry. | Commercial Poultry/Swine/Beef Facilities | **Mucosa (oral/nasal/conjunctival) Inhalation (respiratory)** | The case was confirmed by the Ohio Department of Health. | Not stated | Not stated |
| Pan American Health Organization | 2025 | Timeline – Influenza A(H5N1) Americas Region | Pan American Health Organization | United States (Wyoming) | Report | 1 (confirmed H5N1 case) | Backyard Poultry Owners/Poultry Consumers | Older adult (age not stated) | 1 Female | The backyard flock owner in Wyoming had respiratory symptoms and is reported to have underlying health conditions that can make people more vulnerable to severe influenza illness. This person has been discharged from the hospital and is recovering. **This person had direct contact with poultry infected with avian influenza A(H5) virus that died on their property.** Initial upper respiratory specimens were negative for influenza viruses; a lower respiratory specimen collected several days later in the hospital was positive for avian influenza A(H5N1) virus. | Backyard/Neighborhood Poultry | **Mucosa (oral/nasal/conjunctival) Inhalation (respiratory)** | The case was confirmed by the CDC. Genetic sequencing. | Not stated | Not stated |
